# Natural variation in genes potentially involved in plant architecture and adaptation in switchgrass (*Panicum virgatum* L.)

**DOI:** 10.1186/s12862-018-1193-2

**Published:** 2018-06-14

**Authors:** Bochra A. Bahri, Guillaume Daverdin, Xiangyang Xu, Jan-Fang Cheng, Kerrie W. Barry, E. Charles Brummer, Katrien M. Devos

**Affiliations:** 1 Institute of Plant Breeding, Genetics and Genomics (Department of Crop and Soil Sciences), and Department of Plant Biology, University of Georgia, Athens, GA 30602 USA; 2 Laboratory of Bioaggressors and Integrated Protection in Agriculture, The National Agronomic Institute of Tunisia, University of Carthage, 43 Avenue Charles-Nicolle, 1082 Tunis, Tunisia; 3 DOE Joint Genome Institute, Walnut Creek, California, CA 94598 USA; 4 Plant Breeding Center, Plant Sciences Department, University of California, Davis, CA 95616 USA; 5 Present address: Vinson Edward Ltd, Faversham, ME13 8UP UK; 6 Present address: USDA-ARS, Wheat, Peanut and Other Field Crops Research Unit, Stillwater, OK 74075 USA

**Keywords:** Single nucleotide polymorphisms, Biomass genes, Selection, Subgenome, Population structure, Evolution

## Abstract

**Background:**

Advances in genomic technologies have expanded our ability to accurately and exhaustively detect natural genomic variants that can be applied in crop improvement and to increase our knowledge of plant evolution and adaptation. Switchgrass (*Panicum virgatum* L.), an allotetraploid (2n = 4× = 36) perennial C4 grass (*Poaceae* family) native to North America and a feedstock crop for cellulosic biofuel production, has a large potential for genetic improvement due to its high genotypic and phenotypic variation. In this study, we analyzed single nucleotide polymorphism (SNP) variation in 372 switchgrass genotypes belonging to 36 accessions for 12 genes putatively involved in biomass production to investigate signatures of selection that could have led to ecotype differentiation and to population adaptation to geographic zones.

**Results:**

A total of 11,682 SNPs were mined from ~ 15 Gb of sequence data, out of which 251 SNPs were retained after filtering. Population structure analysis largely grouped upland accessions into one subpopulation and lowland accessions into two additional subpopulations. The most frequent SNPs were in homozygous state within accessions. Sixty percent of the exonic SNPs were non-synonymous and, of these, 45% led to non-conservative amino acid changes. The non-conservative SNPs were largely in linkage disequilibrium with one haplotype being predominantly present in upland accessions while the other haplotype was commonly present in lowland accessions. Tajima’s test of neutrality indicated that *PHYB,* a gene involved in photoperiod response, was under positive selection in the switchgrass population. *PHYB* carried a SNP leading to a non-conservative amino acid change in the PAS domain, a region that acts as a sensor for light and oxygen in signal transduction.

**Conclusions:**

Several non-conservative SNPs in genes potentially involved in plant architecture and adaptation have been identified and led to population structure and genetic differentiation of ecotypes in switchgrass. We suggest here that *PHYB* is a key gene involved in switchgrass natural selection. Further analyses are needed to determine whether any of the non-conservative SNPs identified play a role in the differential adaptation of upland and lowland switchgrass.

**Electronic supplementary material:**

The online version of this article (10.1186/s12862-018-1193-2) contains supplementary material, which is available to authorized users.

## Background

A major challenge in crop improvement is to achieve food and energy security by identifying genetic polymorphisms that directly influence traits of economic importance. In switchgrass, we are particularly interested in biomass yield and composition. Next generation sequencing technologies have made accurate detection of genomic variation, including single nucleotide polymorphisms (SNPs), insertions/deletions (INDELs), copy number variants, and presence/absence variants feasible. Association of these variants with agronomic traits via association mapping and/or biparental linkage mapping has greatly facilitated crop breeding and has helped to address the increased global demand for food, feed, fiber, and fuel [1–8]. The advances in genomic technologies have also expanded our knowledge of biological processes, evolution, and adaptation.

A number of genes involved in agronomic and/or adaptive traits have been identified using quantitative trait loci (QTL) analyses and association mapping in breeding and natural populations, and the functional variants validated using transgenic experiments [9–18]. Natural variation in these genes is often shaped by selection for better agronomic performance or adaptation to specific environmental conditions, such as day length (photoperiod) and temperature. Switchgrass (*Panicum virgatum* L.), a species displaying multiple cytotypes and a wide geographic distribution, constitutes an ideal system for the study of selective pressure associated with environmental gradients, as evidence of local adaptation has been confirmed through numerous agronomic field trials and reciprocal transplant experiments [19–21].

Switchgrass is a warm season, C4 perennial native to North American tall grass prairies. It is an economically and ecologically important species that has numerous applications including as forage, for habitat restoration, and for soil and water conservation. It has been selected by the US. Department of Energy as a promising biofuel crop because of its high biomass yield, its adaptability to marginal lands, its low production costs and its low nutrient and water requirements [22, 23]. Switchgrass is a largely outcrossing polyploid species that is classified into upland and lowland ecotypes based on phenotypic and physiologic differentiations, and habitat preference. Upland ecotypes are commonly octoploid (2n = 8× = 72) and occasionally hexaploid (2n = 6× = 54) or tetraploid (2n = 4× = 36) while lowland ecotypes are largely tetraploid (2n = 4× = 36) [24, 25]. Photoperiod and temperature factors have led to physiological variations and a strong climatic adaptation along a north-south gradient, with upland accessions flowering earlier than lowland switchgrass across all latitudes [26, 27]. Upland accessions are more adapted to the northern US while lowland accessions are more adapted to southern regions with a transition zone where both ecotypes coexist. The upland ecotype is shorter than the lowland ecotype with more tillers per plant, shorter leaf blades with various amounts of pubescence, and a reduced stem diameter [28]. Different genetic markers have been associated with upland-lowland ecotype classifications including random amplified polymorphic DNA [27, 29, 30], restriction fragment length polymorphisms [24, 31], expressed sequence tag-simple sequence repeats (EST-SSRs) [32], and chloroplast markers [33–35]. The existence of genotypes with intermediate morphological form, occurrence of mixed ploidies within some lowland accessions, inconsistent ecotype classification using nuclear and cytoplasmic DNA markers, and similarity in marker orders and distribution of recombination events between upland and lowland ecotypes have suggested the possibility of significant gene flow and chromosomal exchanges between the two ecotypes [35–37].

Both phenotypic and molecular analyses have demonstrated that there is extensive genetic diversity within and among populations in this highly heterozygous species [38, 39]. There is thus significant potential for genetic improvement of this non-domesticated grass [22, 40–43]. Early switchgrass breeding programs have focused largely on improving forage quality for livestock production systems. By emphasizing use of switchgrass as an energy crop, the principal breeding objectives have switched to improving biomass yield and biomass composition, and reducing recalcitrance [44]. Phenotypic, genetic and genomic resources for switchgrass are currently available, including bacterial artificial chromosome libraries [45], expressed sequence tags [46], an exome capture array [47, 48], an assembled genome sequence of the switchgrass lowland genotype Alamo AP13 (phytozome.jgi.doe.gov), several biparental mapping populations [36, 49–51], as well as two association mapping panels (a northern and a southern US panel) [47]. Knowledge of the genomic variability for traits of interest and the population structure present in the switchgrass panels will enable efficient identification of marker-trait associations, and significantly speed up selection of alleles that enhance bioenergy feedstock production. Research investigating local adaptation and the genomic variability for adaptive traits such as pest resistance, stress tolerance, biomass yield and quality, and phenology would provide the foundation for expanding the cultivation range of switchgrass accessions through targeted improvements.

The objectives of this research were i) to analyze SNP patterns among 372 switchgrass genotypes for genes putatively involved in biomass production; ii) to investigate whether a genetic signature of selection could be identified that led to ecotype differentiation; and iii) to uncover putative relationships between genetic variation and geographic zone, and identify loci underlying local adaptation by inferring variability associated with fine-scale differentiation.

## Methods

### Sample collection and DNA extraction

The germplasm used in this study consisted of 36 switchgrass accessions representing a wide range of phenotypic variation including for biomass traits. For each accession, two to 15 individuals from the same cultivar or sampled at the same geographic location for a total of 372 genotypes were analyzed. Some switchgrass cultivars were derived from seed increases from source-identified remnant prairies with no or little breeding history and thus represented the natural genetic variation within specific regions. Twenty-one accessions were identified phenotypically and confirmed by analysis of the chloroplast trnL (UAA) intron deletion as lowland ecotypes (215 genotypes) and 15 as upland ecotypes (157 genotypes) [52]. Forty-five percent of the accessions were tetraploid, 14% were octoploid and the rest had mixed or unknown ploidy levels. The accession numbers or names, number of genotypes analyzed per accession, ecotype identifier, ploidy level, and origin of the accession including global positioning system (GPS) coordinates of the collection sites are presented in Additional file 1: Table S1.

Approximately 15 mg of young leaves were collected from each genotype and kept at -20 °C until DNA extraction. The tissue was disrupted and homogenized with the TissueLyser II (QIAGEN), and total DNA was extracted using a cetyltrimethylammonium bromide (CTAB) method [53]. DNA quality and integrity were checked on a 1% agarose gel stained with ethidium bromide. DNA concentrations were measured using a NanoDrop NP 1000 spectrophotometer (NanoDrop Technologies, Wilmington, DE).

### Primer design and PCR amplifications

A list of 17 candidate genes representing possible targets for modification of biomass production was compiled from the published literature. Because the switchgrass genome sequence was not available at that time, two to four primer pairs per gene, each spanning an approximately 1 kb genomic region, were designed against conserved regions in orthologous exons of *Oryza sativa* (rice), *Sorghum bicolor* (sorghum) and *Setaria italica* (foxtail millet) using Primer Premier 5.0 software [54]. Test Polymerase Chain Reaction (PCR) amplifications in two to four switchgrass genotypes were done in a total volume of 20 μl consisting of 50 ng genomic DNA, 0.4 μM of each primer, 0.8 U of GoTaq DNA polymerase (Promega, Madison, WI), 1.5 mM MgCl_2_, and 0.2 mM dNTPs in 1X buffer. After an initial denaturation at 94 °C for 5 min, PCR amplification was performed for 35 cycles of denaturation at 94 °C for 30 s, annealing at the primer melting temperature (Tm ^0^C) for 30 s, and primer extension at 72 °C for 30 s. The final extension was held at 72 °C for 10 min after which the samples were cooled to 10 °C. PCR products were separated on 1% (*w*/*v*) agarose gels stained with ethidium bromide and sequenced using the Sanger method. A total of 12 genes (33 primer sets) for which single fragments were obtained in the test PCR for multiple ~ 1 kb regions and for which the amplicon identity was confirmed by Sanger sequencing were selected for amplification in the complete panel of 372 switchgrass genotypes. The sequences and annealing temperatures used for these 33 primer pairs are given in Additional file 1: Table S2. The PCR conditions used for the full panel were the same as for the test amplification. Amplicons were separated and visualized on 1% agarose gels and quantified using a NanoDrop NP 1000 spectrophotometer (NanoDrop Technologies, Wilmington, DE). PCR products were pooled by genotype with equal representation of the 33 amplicons in each pool. The DNA in each pool was purified using Agencourt’s AMPure XP magnetic beads, eluted in 100 μl of TE Buffer (10 mM Tris, 0.1 mM EDTA, pH 7.6), quantified with a Qubit fluorometer, and diluted to 100 ng/μl. Additional quality control tests were performed for 28 random DNA samples on agarose gels using Joint Genome Institute (JGI) standard kits, including six mass standards with a molecular weight ranging from 3.1 ng/μl to 100 ng/μl.

### Library preparation and Illumina sequencing

Sequencing of the purified amplicons from the 372 switchgrass genotypes was conducted at the JGI. For each genotype, 5 ng of amplicon DNA was used for concatenation and Illumina library construction to reduce coverage bias of certain regions of amplicons. Briefly, amplicons from a single switchgrass genotype were pooled together, end repaired using the NEBNext End Repair Module, and ligated to form concatemers using the NEBNext Quick Ligation Module. The shearing of concatenated DNA and Illumina fragmented library construction followed the manufacturer’s protocol (Illumina, Inc.). Each pool of amplicons was ligated to a different barcoded adaptor. Barcoded libraries were pooled and sequenced (2 × 100 bp) on a single lane of an Illumina HiSeq 2000 platform. The raw Illumina reads were quality trimmed to remove low quality reads (PHRED score < 20) and short reads (< 30 bp), and separated into different bins based on barcode reads.

### Single nucleotide polymorphism calling

The cleaned Illumina reads were exported in FASTQ format, and aligned using Bowtie 2.2.3 [55] with default parameter settings to 56 contigs extracted from the switchgrass AP13 genome sequence version 0.0 (phytozome.jgi.doe.gov) based on their high homology to the amplicon sequences. For each amplicon, the first two to six AP13 hits with a minimum BLASTN score of 100 and an E value threshold of 1.0E^− 28^ were selected. The selected contigs comprised homologous, homoeologous as well as paralogous sequences (see Additional file 1: Table S3 for the reference sequences used). Mapped reads were sorted and indexed with SAMtools software version 1.2 [56]. The assembled reads were mined for SNPs using GATK version 3.4.0 and GATK-Unified Genotyper as SNP caller [57]. The filtering thresholds were set as follows: base quality score ≥ 20, read mapping quality ≥10, and unlimited read coverage. We refer to nucleotide changes as sequence variations from the AP13 reference sequences. INDELs were not included in this study, and adjacent SNPs were classified as biologically unlikely and discarded. SNPs with less than 20% of missing data, a frequency in the population between 5 and 95%, and a read depth ≥ 3 were analyzed. Genotypes with more than 30% of missing data were discarded. Within genotypes, SNPs with an allele frequency < 25% or > 75% were considered homozygous (94% of them had frequencies ≤10 or ≥ 90), while SNPs with an allele frequency between 40 and 60% were considered heterozygous.

### Data analysis

#### Overall genetic diversity and SNP analysis

The distributions of allele frequencies within genotypes (bin size 0.1) and of variants across the amplicons (bin size 50 bp) were assessed in the total SNP dataset. The frequencies of heterozygous SNPs vs. homozygous SNPs, the overall SNP density, and the overall genetic differentiation G_st_ were calculated.

The open reading frames for each gene were used either as annotated or as determined by sequence comparison with gene models from *S. italica* (Gramene: http://www.gramene.org) to estimate diversity at non-synonymous and synonymous sites in exons. For non-synonymous SNPs, the wild-type allele at a SNP locus was defined as the allele that was present in *S. italica* and hence was likely ancestral. If both the reference allele (the allele present in the AP13 reference genome) and the alternate allele differed from the allele present in *S. italica*, the allele with the highest frequency in switchgrass was considered the wild-type. Rare alleles were defined as having frequencies ≤0.25 or ≥ 0.75 in the entire panel relative to the likely ancestral nucleotide, common alleles as having frequencies > 0.25 and < 0.40 or > 0.60 and < 0.75, and balanced alleles as having frequencies ≥0.40 and ≤ 0.60. In addition, at each SNP position, frequencies of the wild-type allele in the different genetic subpopulations as defined by STRUCTURE analysis were calculated. A SNP was considered prevalent in a genetic subpopulation when it was present in only one subpopulation, or when its frequency was ≥0.75 in a single subpopulation and < 0.25 in each of the other genetic subpopulations. A SNP was considered diagnostic for a genetic subpopulation when it was present in one subpopulation at a frequency ≥ 0.50 and at frequencies < 0.05 in each of the other genetic subpopulations. Non-synonymous substitutions that led to property changes in the corresponding amino acids were classified as non-conservative SNPs according to the blast results on NCBI.

#### Population structure analysis

The SNPs were used to perform a population structure analysis using a Bayesian clustering algorithm implemented in STRUCTURE v.2.3.4 [58]. Ten runs of STRUCTURE using the admixture model, a burn-in period of 100,000 replications and a run length of 100,000 Markov Chain Monte Carlo (MCMC) iterations were carried out for a number of clusters ranging from K = 1 to K = 10. The optimum value of K was determined using the *ad hoc* criterion, based on the log probability of data [LnP(D)] [58]. At the optimal K-value, the membership coefficient from the run with the lowest likelihood value was used to determine for each genotype the proportion of the genome that belonged to each inferred population. Each individual was assigned to the subpopulation to which it had the highest membership. Genotypes with affiliation probabilities (inferred ancestry) < 70% to any single subpopulation were considered “admixed”. The overall coefficient of gene differentiation (G_st_) was calculated on the basis of Nei’s method and its significance was tested using 999 permutations and bootstraps in GenAlEx 6.501 [59]. The estimate of gene flow (N_m_) between subpopulations as defined by STRUCTURE was calculated from G_st_ as N_m_ = 0.5(1− G_st_)/G_st_. The significance of subpopulation differentiation defined by STRUCTURE was further investigated by performing a Principal Coordinates Analysis (PCoA) using GenAlEx 6.501. An Analysis of Molecular Variance (AMOVA) implemented in GenAlEx 6.501 was used to investigate the percentage of molecular variability explained by the genetic populations.

#### Phylogeographic analysis

To further investigate the differences between the switchgrass genotypes, pairwise Fst and Nei’s genetic distance matrices were calculated using GenAlEx 6.501. All the DNA sequences were concatenated into one contiguous sequence for each switchgrass genotype. Variants detected across the concatenated sequences were used to perform an Unweighted Pair Group Method with Arithmetic mean (UPGMA) tree based on the maximum composite likelihood method with a 500 replicates bootstrap test in the program Mega 6 [60]. Divergence times between subpopulations were calculated using a relative divergence time of 13 million years between switchgrass and its close relative foxtail millet (*Setaria italica*) as standard [61]. An AMOVA implemented in GenAlEx 6.501 was used to investigate the percentage of molecular variability explained by the geographic origin and latitudinal adaptation of the accessions. Genotypes were classified according to their latitude in 1 degree bins. An AMOVA by accessions was also performed; each accession corresponded to one geographic location. In addition, the correlation between genetic and geographic distance was analyzed for the entire population as well as for each subpopulation independently using a Mantel test [62] implemented in GenAlEx 6.501. To determine whether fine-scale spatial genetic structure was present within geographic regions, a local spatial autocorrelation analyses was performed in GenAlEx using the 2D-Local Spatial Analysis algorithm (2D-LSA). Each individual was tested for genetic relatedness to its *n* nearest geographic neighbors in order to identify fine-scale patches of lower genetic diversity. Significance levels were estimated through 9999 random permutations of the samples. Analyses were performed for 7 to 14 nearest neighbors (*n*) to determine the consistency of the observed patterns.

#### Genetic diversity within genes, subpopulations and subgenomes

For each contiguous DNA sequence, DnaSP 5.10 [63] was used to calculate the nucleotide diversity π, Watterson’s estimator theta (per site) θ and its standard deviation. To test the neutral mutation hypothesis, per-gene basis Tajima’s D test [64] was performed in DnaSP 5.10 in both the entire dataset and within subpopulations. In addition, the PHASE algorithm [65], as implemented in DnaSP 5.10, was used for haplotype reconstruction. The algorithm was run for 1000 Markov Chain Monte Carlo iterations with a burn-in of 1000 iterations. Comparisons between the three subpopulations defined by STRUCTURE were performed on concatenated sequences for each individual for all measures of genetic diversity. The number of effective alleles (Ne), number of haplotypes (Nh), percentage of polymorphic loci (P), Shannon’s Information Index (I), observed and expected heterozygosity (Ho, He) and fixation index (F) were assessed. One Way ANOVAs were performed under R 3.2.2 [66] to statistically test for differences between the subpopulations for Ne, I, Ho and F indexes. This was followed by a TukeyHSD test when the difference was significant (*P* ≤ 0.05). Accessions for which more than 70% of the genotypes belonged to a specific subpopulation were considered representative for that subpopulation. The molecular variances due to differences between subpopulations, within subpopulations and within individuals were calculated using an AMOVA approach in GenAlEx 6.501. This AMOVA helped us to investigate the percentage of molecular variability explained by the genetic structure, as compared to the earlier described AMOVAs which were based on geographic origin and latitude patterns. In addition, we performed a correlation analysis using the rcorr function under R 3.2.2 [66] to test whether the percentage of polymorphic loci was correlated with latitude. Comparisons between the two subgenomes were performed on the percentage of polymorphic loci for each gene and on the haplotype diversity, nucleotide diversity π and Watterson’s estimator theta (per site) θ only for those regions for which SNP information was available in both subgenomes. The chromosomal location of each contig was extracted from the AP13 reference genome assembly v3.1 in Phytozome (http://www.phytozome.net/). By taking a window of 100 bp, divergence between the subgenomes was investigated for the *Phytochrome B* (*PHYB)*, *Terminale ear* (*TE)*, *Flowering locus T* (*FLT),* and *Phosphoglyceratemutase (PGM)* genes.

## Results

### Mapping results and sequence polymorphism

**Fig. 1:**
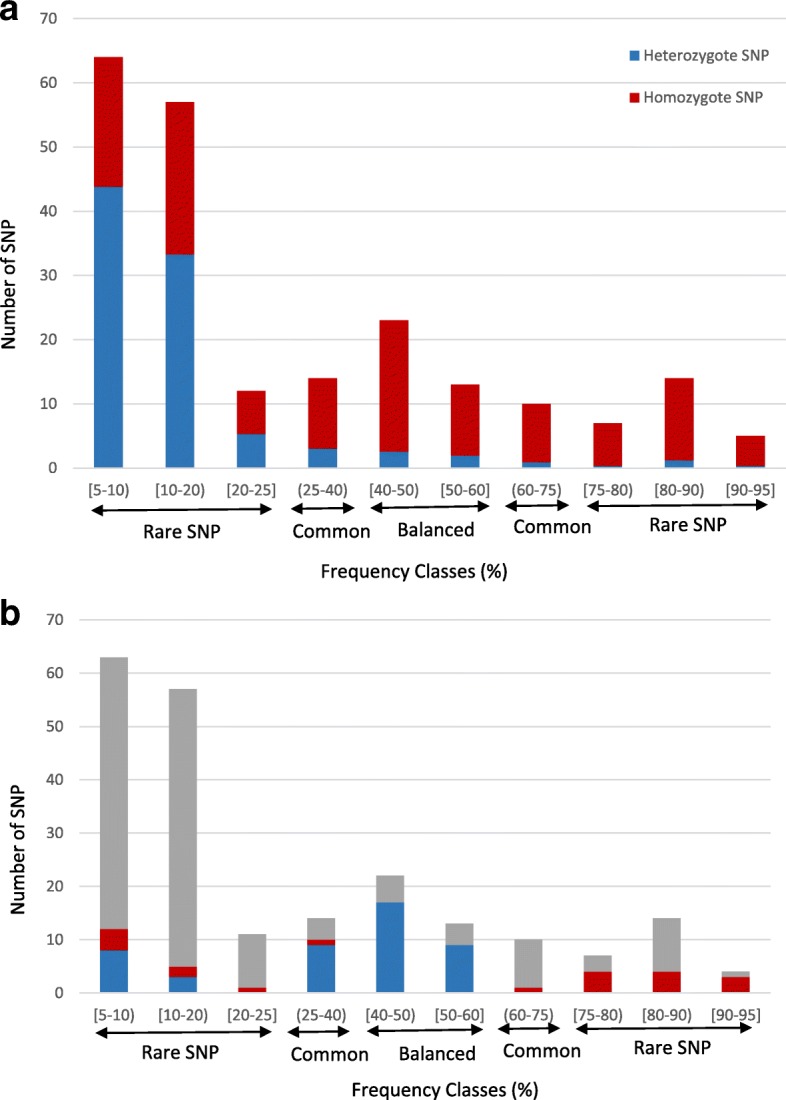
Number of SNPs in different classes of SNP frequencies and their heterozygosity level (**a**) (Heterozygous and homozygous SNPs are indicated in blue and red respectively) or their ecotype prevalence (**b**) (Blue indicates SNPs predominantly present in upland ecotypes (C1), red indicates SNPs predominantly present in lowland ecotypes (C2 and C3) and grey indicates SNPs that occur with similar frequencies in both ecotypes)

A total of 332 million (M) raw Illumina reads were obtained (average read length: ~ 90 bp) for the 33 amplicons generated from 12 genes. Of these, 166 M mapped to 30 of the 56 selected reference contigs with a minimum of 1 M reads per contig, yielding an average read depth of ~ 8.6× per base per sample. The 30 contigs covered homoeologous regions in the 12 genes (Additional file 1: Table S4). A total of 11,788 sequence variants were detected in the dataset of which 11,682 (99.1%) were nucleotide substitutions and the remaining 0.9% were INDELs. After filtering out adjacent SNPs, SNPs with minor allele frequencies (≤ 5%), and SNPs with ≥20% of missing data, 251 SNPs remained across 21 contigs that were used for further analyses (Table 1). Of the 251 SNPs, 33 and 67% were present in exons and introns, respectively. The overall SNP density was 1 SNP/127 bp. However, the majority of SNPs within a contig were spaced < 50 bp apart (Additional file 1: Figure S1). Ninety-four percent of the SNPs (236 SNPs) identified were biallelic and 6% (15 SNPs) were triallelic. Seventy-two percent of the SNPs (181 SNPs) were rare variants. Eighty-six percent of SNPs (60 SNPS) with allele frequencies in the population > 0.25 and < 0.75 were present in homozygote condition in the genotypes (Fig. 1A). In addition, 72% (26 SNPs) of the balanced SNPs (with overall allele frequencies ≥0.40 and ≤ 0.60) had significantly different allele frequencies in upland and lowland ecotypes (Fig. 1B). In *TE* and *FLT*, 83% (35 SNPs) and 100% (38 SNPs) of the SNPs were located in intron 4, and in the 5’ UTR region, respectively. Sixty percent of SNPs (49 SNPs) located in exons were non-synonymous. Of the non-synonymous SNPs, 65% (32 SNPs) encoded an amino acid that had different properties than the amino acid encoded by the reference allele and were termed non-conservative (Table 2; Additional file 1: Table S5). Overall, 78% of common SNPs (7 SNPs) and 62% of rare SNPs (22 SNPS) in exonic regions were non-conservative. In 66.6% of the cases (30 SNPS), the reference amino acid corresponded to the wild-type allele in *Setaria*. In addition, in 84% of the cases (41 SNPS), the wild-type allele was the most frequent allele.

**Table 1:** Summary statistics for the 251 SNPs analyzed in 12 biomass genes

					Polymorphism						Nucleotide diversity				Tajima’s test
								Non Syn							
Gene	Contig	Chromosome	No. of samples	No. of sites (coding length bp)	Stot	Non Cod	Syn	Con	Non Con	d	π_tot_ × 10^-3^ (SD × 10^-3^)	θw × 10^-3^ (SD × 10^-3^)	h	Hd (SD)	D
PHYC	contig03093	Chr09N	353	3224 (2279)	20	7	6	2	5	0.620	1.68 (0.04)	0.87 (0.25)	91	0.963 (0.002)	0.362
TE	contig99597	Chr05K	351	2088 (278)	21	21	0	0	0	1.006	3.43 (0.08)	1.40 (0.31)	84	0.883 (0.010)	0.138
TE	contig04674	ChrOSNa	334	2088 (278)	21	20	1	0	0	1.006	3.78 (0.09)	1.58 (0.34)	110	0.959 (0.003)	0.316
VRN3	contig07490	Chr03Na	337	2258 (1771)	12	1	4	2	5	0.531	1.30 (0.05)	0.74 (0.21)	22	0.759 (0.013)	0.723
VRN3	contigl6433	Chr03K	356	2258 (1773)	10	3	1	3	3	0.443	0.95 (0.03)	0.62 (0.19)	30	0.728 (0.016)	0.62
DW3	contig26301	Chr06K	327	2207 (2207)	5	2	0	2	1	0.227	0.56 (0.02)	0.32 (0.14)	10	0.629 (0.018)	1.267
DW3	contigll7938	Chr06N	337	2198 (2198)	4	2	0	1	1	0.182	0.55 (0.02)	0.25 (0.13)	14	0.714 (0.010)	0.793
FLD	contigl02960	Chr07K	346	2329 (1395)	3	2	1	0	0	0.129	0.10 (0.01)	0.18 (0.10)	5	0.207 (0.020)	−0.588
FLD	contig01920	Chr07N	339	2330 (1395)	1	0	0	0	1	0.043	0.15 (0.01)	0.06 (0.06)	2	0.346 (0.017)	1.253
Gl	contigl5400	Chr05K	342	3456 (1185)	20	13	2	1	4	0.608	1.71 (0.06)	0.81 (0.18)	83	0.904 (0.006)	−0.264
Gl	contig01489	ChrOSN	312	3464 (1185)	10	7	0	1	2	0.289	1.05 (0.03)	0.40 (0.13)	22	0.757 (0.013)	1.655
FLT	contig09545	Chr07K	365	1826 (195)	17	17	0	0	0	0.931	1.97 (0.05)	1.30 (0.31)	64	0.931 (0.005)	−0.103
FLT	contig08422	Chr07N	339	2181 (195)	21	21	0	0	0	0.963	2.85 (0.04)	1.34 (0.29)	62	0.899 (0.006)	0.63
PHYB	contigl3571	Chr09N	350	3198 (2758)	4	1	0	1	2	0.125	0.55 (0.01)	0.17 (0.09)	7	0.697 (0.009)	3.265**
PHYB	contig21054	Chr09K	342	3194 (2758)	7	6	1	0	0	0.219	0.49 (0.01)	0.30 (0.12)	12	0.666 (0.012)	0.771
HD1	contig03275	Chr04K	306	2220 (1104)	7	2	2	0	3	0.360	0.93 (0.44)	0.44 (0.17)	8	0.776 (0.008)	2.082
HD1	contig05584	Chr04N	363	2140 (1113)	17	17	0	0	0	0.280	1.66 (0.04)	1.11 (0.27)	30	0.901 (0.005)	−0.142
PGM	contigl7299	Chr09K	343	3120 (1634)	8	5	2	0	1	0.256	0.59 (0.36)	0.36 (0.13)	28	0.848 (0.008)	1.27
PGM	contig200892	Chr09N*	315	696 (391)	7	4	3	0	0	1.006	3.3 (0.05)	1.40 (0.53)	13	0.780 (0.007)	1.328
TB1	contig06045	Chr09K	339	2428 (1109)	10	2	5	1	2	0.412	1.42 (0.03)	0.59 (0.21)	38	0.817 (0.010)	1.625
TB1	contig76312	Chr09N	314	2597 (1115)	26	16	5	3	2	1.001	2.53 (0.07)	1.41 (0.37)	84	0.926 (0.005)	0.037
Total				51,500 (28313)	251	169	33	17	32						
Sugenome Nb			372	6709 (2884)	106	73	12	9	13		1.61(0.04)	0.65(0.31)	610	0.99(0.20)	−0.353
Sugenome Kb			372	6709 (2884)	100	67	13	6	10		0.31(0.03)	0.53(0.11)	637	0.99(0.10	−0.269

*Stot* Total No. of polymorphic sites, *Non Cod* No. of SNPs in non-coding regions, *Syn* No. of synonymous SNPs, *Con* No. of conservative amino acid changes, *Non Con* No. of non-conservative amino acid changes, *d* Percentage polymorphism (SNP density), *π*_*tot*_ Nucleotide diversity, *θw* Theta per site from Watterson estimator, *D* Tajima’s D, *h* No. of haplotypes, *Hd* Haplotype diversity, *SD* Standard deviation

^a^Chromosome and subgenome allocation were corrected according to mapping data (P Qi and KM Devos, unpublished data)

^b^Subgenome comparisons were limited to overlapping regions

**Table 2:** Characteristics of non-synonymous mutations in biomass genes and corresponding amino acid changes

						Amino Acid				Allele frequencies %			SNP characteristics
Gene	Contig	Chromosome	Amino Acid position in Setaria Reference sequence	SNP region	Setaria italica reference sequence	Ref	Alt1	Alt2	Setaria italica	Wild-type allele	Mutant 1	Mutant 2	Conservative (Con) vs. non conservative (Non Con)
GI	contig15400	Chr05K	155	Exon7	Si000107m	E	K		E	91.59	8.41		Con
GI	contig15400	Chr05K	175	Exon7	Si000107m	S	Y		S	80.32	19.68		Non Con
GI	contig15400	Chr05K	177	Exon7	Si000107m	G	D		D	32.80	67.20		Non Con
GI	contig15400	Chr05K	180	Exon7	Si000107m	G	R		G	92.79	7.21		Non Con
GI	contig15400	Chr05K	234	Exon9	Si000107m	C	F	Y	C	79.82	19.59	0.58	Non Con
GI	contig01489	Chr05N	63	Exon5	Si000107m	W	S		S	37.50	62.50		Non Con
GI	contig01489	Chr05N	185	Exon7	Si000107m	R	Q		R	65.84	34.16		Con
GI	contig01489	Chr05N	960	Exon14	Si000107m	F	L		L	35.83	64.17		Non Con
PHYB	contig13571	Chr09N	661	Exon2	Si033968m	S	C		S	63.64	36.36		Non Con
PHYB	contig13571	Chr09N	702	Exon2	Si033968m	V	I		V	79.13	20.87		Con
PHYB	contig13571	Chr09N	713	Exon2	Si033968m	Y	D		D	50.30	49.70		Non Con
HD1	contig03275	Chr04K	11	Exon1	Seita.4G122700.1	E	M		E	85.98	14.02		Non Con
HD1	contig03275	Chr04K	32	Exon1	Seita.4G122700.1	A	T		A	94.56	5.44		Non Con
HD1	contig03275	Chr04K	35	Exon1	Seita.4G122700.1	G	S		S	60.63	39.37		Non Con
PGM	contig17299	Chr09K	351	Exon6	Si034948m	A	V		A	73.21	26.79		Non Con
TB1	contig06045	Chr09K	57	Exon1	Si038692m	G	D		G	91.61	8.39		Non Con
TB1	contig06045	Chr09K	213	Exon1	Si038692m	G	D		G	89.76	10.24		Non Con
TB1	contig06045	Chr09K	337	Exon1	Si038692m	I	V		L	56.64	43.36		Con
TB1	contig76312	Chr09N	51	Exon1	Si038692m	H	Y		H	88.06	11.94		Con
TB1	contig76312	Chr09N	89	Exon1	Si038692m	A	P		A	92.42	7.58		Non Con
TB1	contig76312	Chr09N	137	Exon1	Si038692m	S	P		S	92.77	7.23		Non Con
TB1	contig76312	Chr09N	193	Exon1	Si038692m	I	V		V	60.26	39.74		Con
TB1	contig76312	Chr09N	321	Exon1	Si038692m	N	S		N	91.87	8.13		Con
PHYC	contig03093	Chr09N	422	Exon1	Si034030m	V	L		L	45.71	54.29		Con
PHYC	contig03093	Chr09N	966	Exon3	Si034030m	E	V		V	42.90	57.10		Non Con
PHYC	contig03093	Chr09N	1029	Exon3	Si034030m	P	A		P	81.03	18.97		Non Con
PHYC	contig03093	Chr09N	1031	Exon3	Si034030m	K	E		K	76.66	23.34		Con
PHYC	contig03093	Chr09N	1041	Exon3	Si034030m	K	N		K	83.44	16.56		Non Con
PHYC	contig03093	Chr09N	1069	Exon1	Si034030m	L	W		L	94.26	5.74		Non Con
PHYC	contig03093	Chr09N	1104	Exon1	Si034030m	L	H		L	67.70	32.30		Non Con
VRN3	contig07490	Chr03N	238	Exon2	Si021330m	E	D		E	89.78	10.22		Con
VRN3	contig07490	Chr03N	286	Exon3	Si021330m	L	M		L	93.11	6.89		Con
VRN3	contig07490	Chr03N	295	Exon3	Si021330m	T	A		T	90.10	9.90		Non Con
VRN3	contig07490	Chr03N	412	Exon4	Si021330m	P	R		P	91.53	8.47		Non Con
VRN3	contig07490	Chr03N	493	Exon4	Si021330m	L	S		S	84.26	15.74		Non Con
VRN3	contig07490	Chr03N	511	Exon4	Si021330m	N	K		N	93.63	6.37		Non Con
VRN3	contig07490	Chr03N	600	Exon4	Si021330m	G	V		E	88.59	11.41		Non Con
VRN3	contig16433	Chr03K	144	Exon2	Si021330m	P	A		P	90.45	9.55		Non Con
VRN3	contig16433	Chr03K	398	Exon4	Si021330m	V	I		A	93.98	6.02		Con
VRN3	contig16433	Chr03K	409	Exon4	Si021330m	S	N		S	89.42	10.58		Con
VRN3	contig16433	Chr03K	450	Exon4	Si021330m	D	G		G	43.61	56.39		Non Con
VRN3	contig16433	Chr03K	569	Exon4	Si021330m	Q	E		R	88.36	11.64		Con
VRN3	contig16433	Chr03K	682	Exon4	Si021330m	L	Q		Q	28.61	71.39		Non Con
DW3	contig26301	Chr06K	672	Exon3	Si013123m	E	G		E	94.29	5.71		Non Con
DW3	contig26301	Chr06K	751	Exon3	Si013123m	M	V		M	21.22	78.78		Con
DW3	contig117938	Chr06N	713	Exon3	Si013123m	I	M		M	87.84	12.16		Con
DW3	contig26301	Chr06N	897	Exon3	Si013123m	I	V		V	82.96	17.04		Con
DW3	contig117938	Chr06N	872	Exon3	Si013123m	T	A	S	A	78.04	21.96	0.30	Non Con
FLD	contig01920	Chr07N	511	Exon1	Si009376	S	G		G	76.70	23.30		Non Con

### Population structure and gene flow

**Fig. 2:**
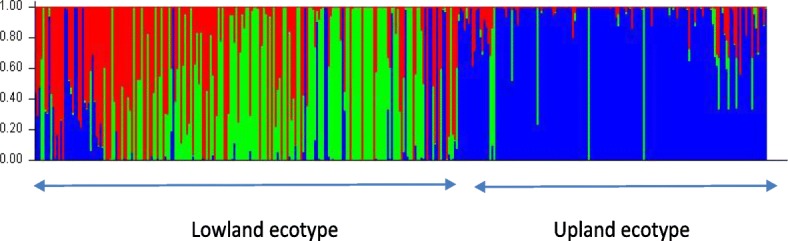
STRUCTURE output assuming K = 3 for 372 switchgrass genotypes based on 251 SNPs. Each genotype is represented by a thin vertical line divided into K colored segments representing the individual’s estimated membership probability to each of K clusters (C1, C2 and C3 are in blue, green and red, respectively). Genotypes were grouped by ecotype

Overall, the genetic differentiation among the 36 accessions of switchgrass was high and significant (G_st_ = 0.454, *P* = 0.001). The Bayesian clustering algorithm implemented in STRUCTURE v.2.3.4 combined with the LnP(D) method indicated the presence of three genetically distinct subpopulations C1, C2 and C3 (Fig.2; Additional file 1: Fig. S2). Thirteen (36%), five (14%) and five (14%) accessions were classified as representative for subpopulations C1, C2 and C3, respectively, and three (8%) were admixed (Table 3). C1 contained 158 genotypes (42% of the total sample), 87% of which were upland ecotypes. Sixty-nine percent of lowland ecotypes grouped into subpopulations C2 (81 genotypes; 22% of total sample) and C3 (71 genotypes; 19% of total sample). Our genetic results revealed that fewer than 7% of individuals fell into a subpopulation different than that expected based on their ecotype phenotype; 20 genotypes with a lowland phenotype clustered in subpopulation C1 and four genotypes with an upland phenotype clustered in C2. Overall, 9.1% of the individuals allocated to one of the three subpopulations (allocation based on highest percentage membership) were admixed (≤ 70% membership to a single subpopulation) C2-C3, 4.3% were admixed C1-C3, 0.5% were admixed C1-C2 and 2.7% were admixed C1-C2-C3 (Fig. 2; Table 3). The percentage of admixed individuals correlated with the amount of interpopulation gene flow N_m_ which was estimated at 1.150 between C2 and C3, 0.791 between C1 and C3, and 0.533 between C1 and C2. Admixed individuals belonged mostly to accessions PI 422003 (octoploid) and PI 476290 (tetraploid) (Table 3). More than 70% of genotypes within both accessions were admixed C2-C3.

**Table 3:** Comparison of genetic diversity between the three switchgrass subpopulations

					Diversity indexes**				
	N	Np	P	Representative accessions*	Ne (SE)	I (SE)	Ho (SE)	He (SE)	F (SE)
C1	158	186(11)	72.9	PI 315724, PI 337553, PI 414066, PI 414067, PI 414068, PI 421520, PI 431575, PI 476292, PI 476294, PI 476295, PI 476296, PI 642190 and PI 642191	1.221^a^ (0.017)	0.236^a^ (0.014)	0.077^a^ (0.005)	0.145^a^ (0.009)	0.402^a^ (0.019)
C2	81	142(3)	55.6	PI 414065, PI 414070, PI 421521, PI 421999 and SNF	1.210 ^a^ (0.019)	0.203 ^a^ (0.015)	0.071^a^ (0.007)	0.129 ^a^ (0.010)	0.361 ^a^ (0.023)
C3	71	200(5)	78.4	PI 315727, PI 422001, Citrus Co-FL, OSSP-FL and SWFWMD-FL	1.211 ^a^ (0.020)	0.217 ^a^ (0.014)	0.050^b^ (0.004)	0.131 ^a^ (0.010)	0.450 ^a^ (0.024)
Admix	62	233(0)	91.4	PI 315725^#^, PI 422003, PI 476290	1.304 (0.018)	0.323 (0.013)	0.078 (0.004)	0.198 (0.009)	0.520 (0.020)
Total	372	251	74.6	26	1.236 (0.009)	0.245 (0.007)	0.069 (0.002)	0.152 (0.005)	0.443 (0.011)

*N* number of genotypes, *Np* No. of polymorphic loci (number of Private Alleles), *P* percentage of polymorphic loci, *Ne* No. of Effective Alleles, *I* Shannon’s Information Index, *Ho* Observed Heterozygosity; *He* Expected Heterozygosity, *F* Fixation Index, *SE* Standard Error

* Accession for which more than 70% of the genotypes belong to a specific subpopulation

**Mean and SE over loci for each subpopulation; Means with the same letter are not significantly different based on a Tukey HSD test

^#^Accession 3 is represented by only two genotypes

**Fig. 3:**
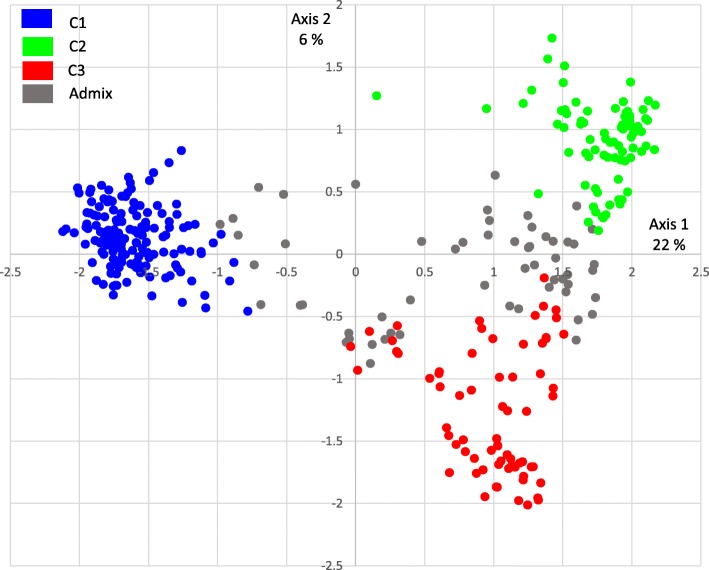
Principal coordinates analysis for 251 SNPs across the switchgrass genotypes. The genotypes are color-coded according to their affiliation to STRUCTURE subpopulations at K = 3. The cluster C1 comprises mainly upland ecotypes whereas genotypes from C2 and C3 are mostly lowland ecotypes

The principal coordinates analysis confirmed the clustering of the switchgrass genotypes into the three groups, C1, C2 and C3, identified by STRUCTURE. The first eigenvector of the PCoA explained 22% of the genetic variability and separated the C1 cluster (mostly upland genotypes) from the C2 and C3 clusters (mostly lowland genotypes). The second axis explained 6% of the genetic variability and separated C2 from C3 (Fig. 3).

### Phylogeographic analysis

Pairwise estimates of *F*_*ST*_ between subpopulations indicated that the highest degree of differentiation was between C1 and C2 (0.313), and the lowest was between C2 and C3 (0.167). Similarly, the largest Nei genetic distance (0.359) was noted between C1 and C2, while the genetic distance between C2 and C3 (0.144) was lowest (Table 4). An UPGMA tree confirmed the STRUCTURE and PCoA analyses (Additional file 1: Fig. S3).

**Table 4:** Pairwise Nei’s genetic distance (upper diagonal) and *F*_*ST*_ values (lower diagonal) among the three switchgrass subpopulations C1, C2 and C3 using 251 SNPs

	C1	C2	C3
C1	0	0.359	0.231
C2	0.313	0	0.144
C3	0.221	0.167	0

**Fig. 4:**
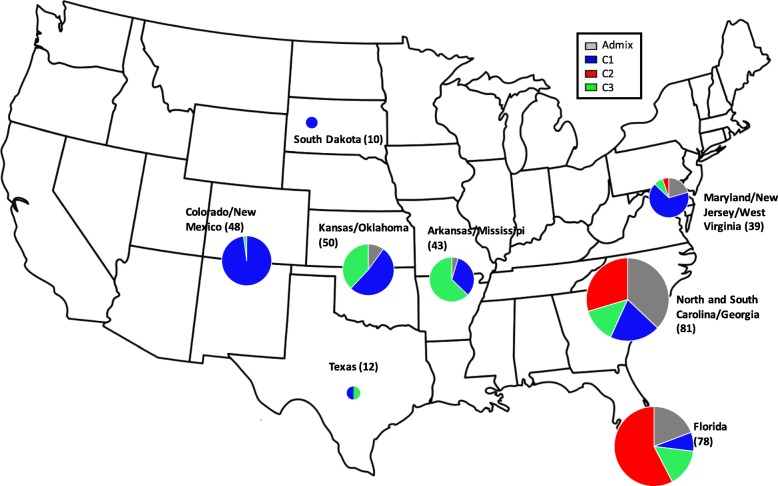
Genetic composition of switchgrass populations by geographic region. The genotypes are color-coded according to their affiliation to STRUCTURE subpopulations at K = 3 (C1: blue; C2: green; C3: red; admixed: gray) and grouped by geographic area. The circle size in each geographic area is proportional to the number of genotypes. USA Map was downloaded from https://upload.wikimedia.org/wikipedia/commons/c/ca/Blank_US_map_borders.svg

The Mantel test revealed a significant positive relationship between geographic and genetic distances (*r* = 0.170; *P* = 0.001) across the whole sampled region, indicating significant isolation-by-distance. Mantel tests conducted separately on each subpopulation also indicated significant isolation-by-distance within C1, C2 and C3 (C1: *r* = 0.171; *P* = 0.005; C2: *r* = 0.270; *P* = 0.001; C3: *r* = 0.313; *P* = 0.01). Positive F_IS_ values in each subpopulation indicated that individuals were more related than was expected under a model of random mating. This suggested that outcrossing occurred predominantly between close neighbors which, in most cases, were derived from the same accession and genetically similar. In addition, the AMOVA indicated that 15 and 28% of the genetic variation were due to differences in latitude range and accession origins, respectively (Table 5A, B). On average, we estimated a change in allele composition of nearly 1% for every 1^0^ of latitude change. C1, C2 and C3 genotypes originated predominantly from the North-Western US, Central-Western US and South-Eastern US, respectively (Fig. 4).

**Table 5:** AMOVAs calculating the percentages of molecular variance explained by latitude bins (A), accessions (B) and genetic subpopulations (C)

Source	df	SS	MS	Est. Var.	%
(A)					
Among Latitude bins	12	6207.75	517.31	8.09	15%
Among Individuals	349	29,954.89	85.83	39.32	72%
Within Individual	362	2605.00	7.20	7.20	13%
Total	723	38,767.64		54.60	100%
(B)					
Among Accessions	35	12,965.07	370.43	14.92	28%
Among Individuals	326	23,197.37	71.16	31.98	59%
Within Individual	362	2605.00	7.20	7.20	13%
Total	723	38,767.44		54.10	100%
(C)					
Among Subpopulations	2	8818.28	4409.14	22.69	37%
Among Individuals	306	22,088.76	72.19	32.61	52%
Within Individual	309	2151.00	6.96	6.96	11%
Total	617	33,058.04		62.27	100%

*df* degrees of freedom, *SS* sum of squares, *MS* mean square

In our 2D-LSA, 69 individuals were found to consistently have significantly higher genetic correlations with their 7 to 14 nearest neighbors (*P* ≤ 0.05). All of these individuals were clustered in subpopulation C1 and represented 90 to 100% of genotypes from six accessions (four prairie-remnant populations, PI 414066, PI 476292, PI 476294 and PI 476295, and two bred cultivars, PI 642190 and PI 642191). These accessions accounted for 70% of the representative accessions for C1 (Table 3) and were mainly located in the Western US but had a broad North-South range. Isolated clusters of relatives were identified in South Dakota, Colorado, Kansas, Arkansas and New Mexico. No isolated clusters of related accessions were found in subpopulations C2 and C3. The 2D-LSA also revealed significant clusters of diversification along the Atlantic Coast where most of the individuals were significantly different from their nearest neighbors, especially around North/South Carolina where genotypes from the three genetic groups and admixed individuals grow in sympatry (Fig.4; Additional file 1: Fig. S4).

### Comparison of the genetic diversity between subpopulations and subgenomes

#### Population comparison

An AMOVA across subpopulations indicated that 37% of the variation was due to differences between subpopulations and 52% was due to differences within subpopulations. Around 11% of the total genetic variance was explained by differences at the genotype level (Table 5C). No significant differences were found between switchgrass subpopulations for Ne, I and F indices (*P* > 0.071), but subpopulation C3 displayed a significantly lower Ho index than C1 and C2 (*P* = 0.003) (Table 3). The similar level of diversity in all three subpopulations was supported by the lack of a significant correlation between population genetic diversity and latitude (*r* = − 0.108, *P* = 0.371) across the switchgrass collection. A regression analysis of the percentage of polymorphic loci and latitude revealed that population diversity remained constant with increasing latitude (Additional file 1: Fig. S5). However, C1 had a larger number (13 total of which 11 were non-synonymous) of alleles that were prevalent compared to C2 (6 alleles) and C3 (4 alleles). In addition, we observed that non-synonymous SNPs that were present in relatively higher frequencies in subpopulations C2 or C3 were predominantly rare (75% of non-synonymous SNPs; 6 SNPs) and non-conservative (75% of non-synonymous SNPs; 6 SNPs). Non-synonymous SNPs that were prevalent in C1, on the other hand, were equally likely to be common or rare (54% of non-synonymous SNPs were common/balanced, 7 SNPs; 46% were rare, 6 SNPs) but were typically non-conservative (77% of non-synonymous SNPs; 10 SNPs). Our phylogenetic analysis indicated that C1, C2 and C3 diverged approximately 2.8 million years ago (Mya).

#### Subgenome comparison

**Fig. 5:**
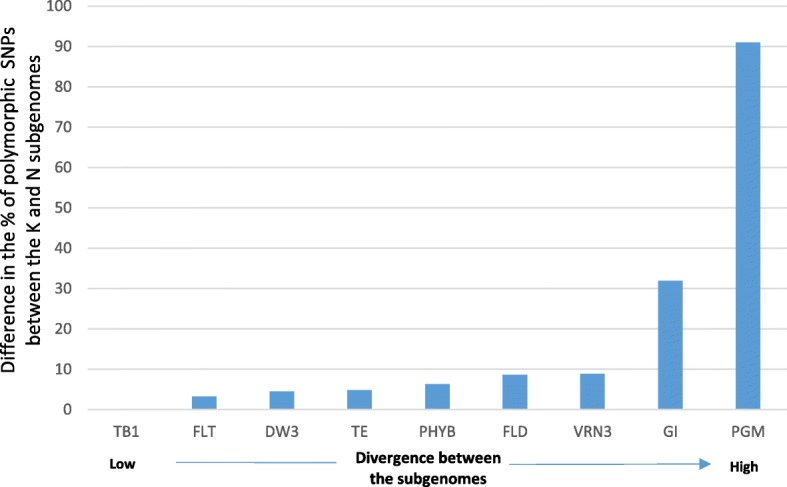
Differences in the percentage of polymorphic SNPs between switchgrass homoeologous regions for selected genes. *TB1*: *Teosinte branched 1*; *FLT*: *Flowering Locus T*; *DW3*: *Dwarf 3*; *TE*: *Terminal ear*; *PHYB*: *Phytochrome B*; *FLD*: *Flowering Locus D*; *VRN3*: *Vernalization 3*; *GI*: *Gigantea*; *PGM*: *Phosphoglycerate mutase*. *PHYC* (*Phytochrome C*), *Rht1* (Gibberellin-insensitve gene) and *HD1* (*Heading date 1*) were removed from the analysis because only genes with mapping data in > 80% of the accessions and overlapping regions between the two subgenomes were used

**Fig. 6:**
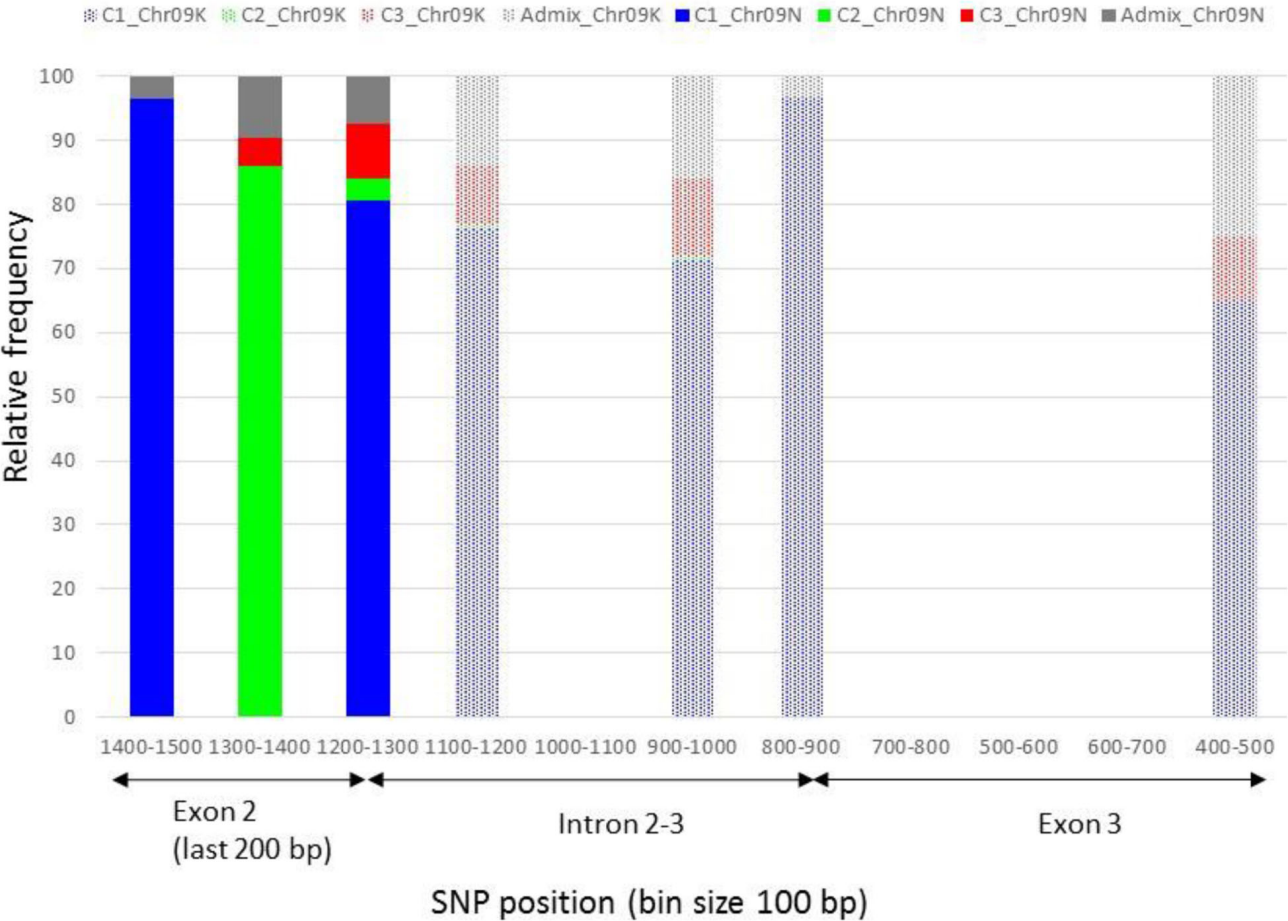
SNP divergence between switchgrass subgenomes N and K in the exon2-exon3 region of *PHYB* in chromosome 9. Relative frequencies of individuals carrying the SNP alleles are color-coded in blue, red, green or gray according to their genetic group as defined by STRUCTURE. Subgenomes N and K are represented in plain and dots, respectively. SNPs are displayed in 100 bp bin sizes

Overall, no significant differences were found between the switchgrass subgenomes in terms of genetic diversity (Table 1). When analyzing homoeologous regions in the two switchgrass subgenomes, the difference in the percentage of polymorphic SNPs present in each of the homoeologous regions was less than 10% except for *Gigantea* (*GI*) which had higher levels of variation in subgenome K and *PGM* which was more polymorphic in subgenome N (Fig. 5). However, region-specific differences in SNP frequencies were observed between the two homoeologous regions in the 5’ UTR of *FLT*, the exon 7–9 region in *PGM*, and the exon 2–3 region in *PHYB* (Fig. 6; Additional file 1: Table S6). In *FLT*, 82% of the SNPs identified in subgenome K (14 SNPs) were present in the first 1 kb of the 5’ UTR region analyzed. In contrast, 67% of the SNPs identified in subgenome N (14 SNPs) were present in the last 1 kb of the 5’UTR region analyzed. Similar observations were made in *PHYB* (Fig. 6) and *PGM* where the majority of the SNPs identified in subgenomes K and N were present in non-overlapping regions. Using the divergence time of 13 Mya between foxtail millet and switchgrass as reference [61], we estimated that subgenomes K and N diverged approximately 5.7 Mya.

### Gene and domain analysis

Ten SNPs were non-conservative and common/balanced across the panel. Four of these led to amino-acid changes in unidentified regions of *GI* (2 amino acid changes), *Vernalization 3* (*VRN3*; 1 amino acid change) and *PHYB* (1 amino acid change) (Table 2). Two SNPs, however, led to non-conservative amino acid changes in the Histidine kinase-related domain of *Phytochrome C* (*PHYC)*, one to a change in the Per-Arnt-Sim (PAS) domain of *PHYB*, one to a change in the Alkaline-phosphatase-like domain of *PGM*, one to a change in the Zinc binding domain of *Heading date 1* (*HD1)* and one to a change in the Fibronectin type III domain of *VRN3* (Table 2). The non-conservative and common SNPs in *PHYB* (Chr09N:102,643,362..102,646,879), *PHYC* (Chr09N:9,701,984..9,705,393) and *GI* (Chr05N:7,182,082..7,180,420) were largely in linkage disequilibrium. One haplotype was prevalent in upland accessions (subpopulation C1) while the other haplotype was commonly present in lowland accessions (subpopulations C2 and C3). For the non-conservative and common SNP in *HD1*, one haplotype was prevalent in subpopulation C2 (lowland accessions) while the other haplotype was commonly present in subpopulations C1 and C3 (upland and lowland accessions, respectively); for the non-conservative and common SNPs in *VRN3*, one haplotype was prevalent in subpopulation C3 (lowland accessions) while the other haplotype was commonly present in subpopulations C1 and C2 (upland and lowland accessions, respectively). However, some accessions were identified where more than 50% of the individuals had a non-conservative SNP at either the *PHYB* (accessions PI 315727 and PI 414067), *PHYC* (accessions PI 414068, PI 421520, PI 642191 and PI 337553), *GI* (accession PI 431575), *HD1* (accessions PI 422016 and SPBluff) or *VRN3* (accessions PI 642191 and Pasco Co-FL) locus that was different from that expected based on the genetic subpopulation to which these genotypes belonged (Table 2). Tajima’s test of neutrality of mutations (Table 1) revealed a significant departure from neutral expectations in the *PHYB* gene copy on subgenome 9 K that carried the two non-conservative SNPs (Tajima’s D = 3.265; *P* < 0.01). A supplementary test of neutrality for this gene with Fu and Li’s F statistic was also significant (Fu and Li’s F = 1.985; *P* < 0.05). Significant positive values of Fu and Li’s F, and Tajima’s D statistics indicated a lack of rare alleles in the *PHYB* gene. Positive values indicate balancing selection or the presence of population structure. Because it is difficult to clearly distinguish between selection and demographic patterns caused by a population bottleneck or population subdivision [67–69], especially with small datasets, per-gene basis Tajima’s D, and Fu and Li’s F tests within each subpopulation were performed. The results showed significant negative values (Tajima’s D = − 1.31, *P* < 0.05; Fu and Li’s F = − 2.91; *P* < 0.05) in *PHYB* (Chr09N) in subpopulation C2, indicating a recent selective sweep (Additional file 1: Table S7). In addition, significant positive values (Tajima’s D = 2.32, *P* < 0.05; Fu and Li’s F = 1.85; *P* < 0.05) were detected in *FLT* (Chr07N) in subpopulation C2, indicating balancing selection (Additional file 1: Table S7). Non-conservative SNPs change the polarity and/or charge properties of the encoded amino acid and, potentially, the three-dimensional structure of the corresponding protein. Protein structure modeling was performed to assess the effect of the amino acid substitution in the PHYB PAS domain using the Swiss-Pdb Viewer 4.1.0 tool [70]. This analysis showed that making the same asparagine to tyrosine substitution in 1D06, a protein with a similar PAS domain as PHYB, as is present in the PAS domain of PHYB, changed the three dimensional structure of 1D06 and, hence, possibly also its activity (Additional file 1: Fig. S6).

## Discussion

### Signature of selection in switchgrass

Local adaptation is an important process that contributes to population differentiation. Over evolutionary time, biotic and abiotic stresses may exert selection on genomic regions and favor different loci depending on the environment, leading to genotypic and phenotypic divergence among populations. We assessed the natural variation present in 51.5 kb of sequence derived from 12 genes affecting biomass traits across a set of 372 diverse accessions of *P. virgatum* and retained, after filtering, 251 SNPs for further analyses. Significant population structure was identified with the upland genotypes largely grouping into one subpopulation (C1) and the lowland genotypes grouping into two subpopulations (C2 and C3). The existence of two lowland genetic populations has previously been reported based on SSRs, SNPs identified by exome-capture, and genotyping-by-sequencing (GBS) [51, 52, 71]. The two lowland subpopulations varied in their morphology. One of the subpopulations (identified as C3 here) had a shorter plant stature and thinner stem diameter than the other (C2 here) [52]. In contrast to earlier studies who reported that differentiation of lowland accessions occurred mainly along a North-South range [51], we found that the gradient of differentiation ran largely West-East. The upland/lowland ecotype division has also previously been demonstrated by chloroplast loci [24, 31], microsatellite loci [34, 37, 72] and multilocus genotypes obtained from sequence data [51, 73]. Considering that upland and lowland accessions are adapted to different environmental conditions, it was not surprising that the switchgrass germplasm was strongly geographically structured. However, this was also the case within ecotypes. An AMOVA indicated that accession origin contributed significantly to the genetic variation. Both Mantel tests and 2D-LSA revealed that genetic differences increased linearly with geographic distance, and that nearby populations tended to be genetically more similar than expected by chance, in particular in the C1 subpopulation. Our results are consistent with previous observations that population structure within *P. virgatum* is associated not only with ecotype but also with latitude and altitude of origin [19–21, 29, 74–80]. Variation between switchgrass ecotypes in a number of phenological traits (spring emergence, flowering time, onset of winter dormancy) has been shown to be driven by the evolutionary divergence of temperature and photoperiodic responses [19, 29, 81–83]. Such patterns of divergence are commonly observed in plant systems, for example in response to winter temperatures, photoperiod, drought, nutrient availability and pest pressure [84–90].

Most of the SNPs in exons were non-synonymous and more than half of them led to non-conservative amino acid changes that, due to changes in charge and/or polarity, might modify the three-dimensional structure of the protein. SNPs differentiating the two ecotypes were mostly fixed in homozygous state in the accessions. This was unexpected as switchgrass is an outcrossing species and previous studies have revealed high levels of heterozygosity in neutral markers [46, 49]. Fixation of these SNPs suggests that they may be located in or associated with genes that play a role in adaptation. We therefore hypothesize that selection played a larger role than drift in ecotype differentiation. Some switchgrass genotypes with overall or regionally low levels of heterozygosity have also been observed in exon capture and re-sequencing data [91].

The degree of divergence between upland and lowland switchgrass ecotypes reflects the balance between selection for adaptive traits/drift and gene flow from nearby populations. Gene flow between natural switchgrass populations belonging to different ecotypes has previously been observed [37]. In our study, genetic exchanges between upland and lowland genotypes were low, most likely because of the difference in ploidy level between the two ecotypes, geographic isolation and pre-mating barriers such as differences in flowering time. As expected, gene flow was higher between the two lowland subpopulations C2 and C3. Nevertheless, our results revealed that the level of gene flow was insufficient to counterbalance genetic drift and/or selection. Natural selection has been shown to overcome ongoing gene flow from morphologically divergent populations in order to maintain phenotypic differentiation in many studies [87, 92–94].

The relative frequency of SNPs that were predominantly present in a single subpopulation indicated that the three populations had been subjected to varying degrees of selection pressure and/or genetic drift. Non-synonymous SNPs prevalent in C2-C3 (lowland) were present at a low frequency, and were mainly rare whereas non-synonymous SNPs prevalent in C1 (upland) were present at higher frequencies and were more likely to be common and non-conservative. This suggests that C1 has been subjected to higher levels of adaptive constraints and/or genetic drift than the C2 and C3 subpopulations. The degree of selection varied by gene and was likely influenced by its level of involvement in adaptation. Tajima’s test revealed a significant deviation from the null hypothesis of neutrality for the *PHYB* gene in contig13571 (Chr09N) in subpopulation C2, suggesting that this gene may have a key role in switchgrass adaptation. Although most genes are present in two homoeologous copies in the tetraploid switchgrass genome, both copies may contribute differently to environmental adaptation. The *PHYB* homoeolog on chromosome 9 K carried no non-synonymous SNPs in the region analyzed and did not appear to be under positive selection. Different evolutionary patterns between subgenomes were also seen in *PGM* and *FLT*. Regional differences in SNP prevalence in these genes could potentially lead to subfunctionalization of the homoeologous gene copies. In addition, in the case of *FLT*, Tajima’s test revealed a significant positive value in subpopulation C2 indicating balancing selection in the chromosome 7 N gene copy but not in its homoeolog on chromosome 7 K. However, for both *PHYB* and *FLT*, evidence for selection should still be interpreted with caution; the confounding effects of population demographic history can mimic the effects of positive selection [67–69] and some demographic models can lead to strong false positive signals in subpopulations [95]. The effects of evolutionary pressure may not be limited to coding regions. In *TE* and *FLT*, 83 and 100% of the SNPs (35 SNPs and 38 SNPs) were in intron 4 and in the 5’UTR region, respectively. Previous studies have shown that the upstream region of the *FLT* gene contained conserved sequences among *Brassicaceae* species that are putative *cis*-regulatory elements that are necessary for *FLT* activation by *CONSTANS* (*CO*) in *Arabidopsis thaliana* [96, 97]. Schwartz et al. [98] fine-mapped a QTL in the *FLT* promoter that contributed to the flowering response to the combined effects of photoperiod and ambient temperature in *A. thaliana*. *Terminal Ear1* (*TE1*) on the long arm of chromosome 3 in maize has been identified as a candidate underlying QTL involved in several traits distinguishing maize and teosinte such as seed number, branching and inflorescence formation [99]. White and Doebley [100] did not find evidence of past selection in a 1.4 kb region of *TE1* that encompassed exons 1 to 3, indicating that this region was probably not involved in maize evolution. *Terminal ear1* on chromosome 3 L in maize is the ortholog of the *TE* gene on switchgrass chromosome 5 analyzed here. Our SNP analysis suggests that the first 600 bp of intron 4 may be an important region involved in gene function. It has been previously shown that intronic SNPs can have functional effects on splicing especially in higher eukaryotes [101–104]. In addition, some intronic polymorphic variants are known to confer susceptibility to disease [105]. Further analyses are necessary to determine if these intronic SNPs have a direct effect on *TE* gene expression and are responsible for a phenotypic polymorphism or whether they are in linkage disequilibrium with a functional SNP.

### Evolutionary events

During repeated glaciation events that impacted tall grass prairie and savanna habitats, switchgrass was massively compressed into refuge areas [106]. Multiple evolutionary processes including genetic drift and selection have influenced the genetic structure of switchgrass that may reflect its post-glacial migratory patterns. Although similar levels of genetic diversity were found in each of the three subpopulations, gene flow analyses supported the south-to-north migration path suggested in previous phylogenetic analyses [37, 51]. In our analyses, alleles prevalent in the lowland subpopulations (C2-C3) and similar to *Setaria* were found at a two times higher frequency than ancestral alleles prevalent in C1, suggesting that the lowland ecotype has a higher number of ancestral alleles and that upland switchgrass originated from lowland switchgrass and migrated north. Furthermore, despite the closer geographic proximity of C1 populations to C2 populations, admixed individuals were eight-fold more frequent between subpopulations C1 and C3 than between C1 and C2 and showed a predominantly lowland haplotype. This suggests that the extreme southern area (Florida), represented by the C3 genetic cluster, was probably the source of the northern upland 4× and upland 8× switchgrass populations. Northward migration was a long process characterized by independent recolonization of northern latitudes from southern refugia [37, 51, 80, 106]. The major environmental factor driving natural selection of *P. virgatum* at more northern latitudes was tolerance to longer day length [35]. Consequently, upland ecotypes (C1 here) flower significantly earlier than accessions belonging to lowland subpopulations (C2 and C3 here) [52].

Only a single major lineage was identified in the upland tetraploids using SSR markers, cpDNA sequences, GBS and exon capture data [35, 51, 71]. While most tetraploid uplands are found in the Midwest region [35, 51, 71], selection for distinct adaptive traits may have allowed their west-east distribution ranging from South Dakota (PI 642191) and New Mexico (PI 642190) to Maryland (PI 476296). Local spatial autocorrelation analyses based on a one-tailed test in which each genotype was compared with its 7 to 14 nearest neighbors revealed significant *P* values for 69 individuals. All individuals that were significantly more related to their geographic closest neighbors than to random individuals belonged to subpopulation C1, indicating higher local adaptation within this genetic group. This fine-scale genetic structure revealed several hot-spots of spatial clusters of related germplasm in C1 accessions in the Western US ranging from South Dakota to New Mexico. These locally adapted populations may be the results of differential patterns of selection, gene flow and genetic drift. Subpopulation C2, located mainly in Kansas, Oklahoma and Arkansas, represents the Southern Great Plains lineage described by Zhang et al. [35] that originated from the glacial refuge on the western Gulf Coast. Subpopulation C3, on the other hand, likely derived from the Eastern Gulf Coast refuge [35]. Differentiation of the C2 and C3 subpopulations was probably driven by selection, in case of C2 for adaptation to a long growing season, high summer temperatures and aridity in the Southern great plains [19, 27, 29, 80, 106] while C3 genotypes selected in the eastern Gulf Coast were more likely characterized by a humid-adapted pattern [35]. While Zhang et al. [35] identified four lowland genetic pools from the Eastern Gulf Coast using SSR markers (‘lowland 4x A’ – 4 accessions, ‘B’ – 1 accession, ‘C’ – 4 accessions and ‘E’ – 1 accession), we only identified a single population. The two accessions from ‘lowland 4x C’ both belonged to subpopulation C3 in our study. The three, one and one accessions analyzed from ‘lowland 4x A’, ‘B’ and ‘E’, respectively, were all classified as admixed in our study. This discrepancy is not due to the nature of the markers used for the diversity analysis. A highly similar population structure with three subpopulations was obtained when the analysis was conducted on the same genotypes analyzed here with 35 SSR markers that identified 365 alleles [52]. More likely, this discrepancy is caused by differences in the composition of the switchgrass panel analyzed and in the population structure interpretations (criteria for the identification of K and the assignment of each individual to a subpopulation). Our 2D-LSA results also revealed a non-random distribution of the spatial clusters of diversification. The eastern USA, especially the Carolinas, emerged as the hot spot of genetic diversity with genotypes from all three genetic subpopulations and most of the admixed individuals. This primary center of diversity along the Atlantic Seaboard has been previously suggested by Zhang et al. [35] based on the presence of 8× individuals with a clear lowland phenotype. Further studies will be necessary to improve our understanding of the forces acting during evolutionary transitions in *P. virgatum* and to reconstruct the patterns of past migrations following glaciation events.

Evolution following migration often begins with divergent selection for locally adapted traits [107]. Mutations in genes underlying traits under divergent selection are expected to be fixed faster than neutral mutations which tend to spread more slowly through large populations [108–110]. Hence, candidate genes potentially under selection provide a better estimate of the upper limit of the divergence time between genetic groups than neutral markers. Our study suggests that upland ecotypes (subpopulation C1) diverged from lowland ecotypes (subpopulations C2 and C3) approximately 2.8 Mya. This estimate, as expected, is somewhat older than the estimated earliest taxonomic divergence between upland and lowland ecotypes based on polymorphisms within the Acetyl-CoA carboxylase locus (1.5–1 Mya, [111]), and within the chloroplast genome (1.3 Mya, [35]). The divergence occurred a sufficiently long time ago for drift to result in divergence even at neutral markers and to create a population structure. In addition, divergent phenotypic selection may drive genetic differentiation at neutral loci if the selection pressure is sufficiently high to reduce the fitness of maladapted migrants [112, 113]. Using the subgenome-specific SNPs, we estimated that the switchgrass subgenomes K and N diverged, at the earliest, 5.7 Mya. Both subgenomes are expected to have evolved at a similar rate since no difference in overall genetic variability was observed.

## Conclusions

SNP variation was assessed in 372 switchgrass genotypes for 12 genes putatively involved in biomass production. Population structure analysis largely grouped upland accessions into one subpopulation and lowland accessions into two additional subpopulations that differed by their local adaptation pattern. Of the 12 genes, *Phytochrome B*, a gene involved in photoperiod response, was shown to be under positive selection in switchgrass subpopulation C2. *Phytochrome B* carried a non-conservative amino acid substitution in the PAD domain, which acts as a sensor for light and oxygen in signal transduction. Further analyses are needed to determine whether this SNP plays a role in the differential adaptation of switchgrass ecotypes.

## Additional file

Additional file 1: **Table S1.** List of switchgrass accessions used in the study with their ID and name, number of genotypes, ecotype identification, ploidy level, state of origin, and GPS coordinates [114–116]. **Table S2.** Sequences and annealing temperatures of the 33 primer pairs used for PCR amplification of the selected 12 genes. Conserved regions in orthologous exons in *Oryza sativa* (rice), *Sorghum bicolor* (sorghum), *Zea mays* (maize) and *Setaria italica* (foxtail millet) were used for primer design. **Table S3.** Sequences of 56 regions of AP13 extracted from the Phytozome database (http://www.phytozome.net/), and used as reference for read mapping and SNP identification. **Table S4.** Number of amplicon reads mapped to each of the 56 reference switchgrass contigs. **Table S5.** Summary statistics for the non-synonymous SNPs analyzed in 12 biomass genes. **Table S6.** Genic regions for which the SNP distribution is different in the K and N subgenomes. The percentage of SNPs and the region in which they are located are given for each subgenome. **Table S7.** Tajima’s, and Fu and Li’s tests on a per gene basis within each subpopulation**. Figure S1.** Distance between SNPs. **Figure S2.** Log probability of data as a function of K. STRUCTURE was run for K ranging from 1 to 10, and 10 repetitions were performed with 100,000 burn-ins and 100,000 runs. K = 3 clusters were retained as the most likely number of genetic clusters in the switchgrass panel analyzed. **Figure S3.** UPGMA tree performed on the 251 SNPs across the 372 genotypes with a 500 replicates bootstrap test using Mega 6 [60] based on the maximum composite likelihood method. C1, C2 and C3 clusters are colored in blue, green and red respectively; admixed individuals are in gray. **Figure S4.** Local Indicator of Spatial Autocorrelation Analysis (2D-LSA) on 372 genotypes. Individuals that are consistently significantly more related to their 7 to 14 nearest neighbors than to random individuals are represented as plain blue dots. The number of genotypes is given in parenthesis. Accessions with significant *P* values for more than 90% of the genotypes are listed; their subpopulation and number of genotypes are indicated. USA Map source: https://upload.wikimedia.org/wikipedia/commons/c/ca/Blank_US_map_borders.svg. **Figure S5.** Regression analysis of the percentage of polymorphic loci and latitude bins across the switchgrass accessions. **Figure S6.** Protein structure modeling of an amino acid substitution in the PAS domain of 1D06, a protein with similar PAS domain as PHYB (A) Original structure of protein 1D06; (B) modified structure after two amino-acid changes in the PAS domain (in yellow): one conservative substitution (Val - > Ile; in green) and one non-conservative substitution (Asp - > Tyr; in pink). Swiss-Pdb Viewer 4.1.0 [70] was used to visualize the crystal structure. (PDF 1034 kb)

## Abbreviations

2D-LSA: 2D-Local Spatial Analysis algorithm
AMOVA: Analysis of Molecular Variance
EST-SSRs: Expressed sequence tag-simple sequence repeats
FLT: Flowering Locus T
GBS: Genotyping-by-sequencing
GI: Gigantea
GPS: Global positioning system
HD1: Heading date 1
INDELs: Insertions/deletions
JGI: Joint Genome Institute
M: million
Mya: million of years ago
PAS: Per-Arnt-Sim
PCoA: Principal Coordinates Analysis
PCR: Polymerase chain reaction
PGM: Phosphoglyceratemutase
PHYB: Phytochrome B
PHYC: Phytochrome C
QTL: Quantitative trait loci
Rht1: Gibberellin-insensitve gene
SNP: Single nucleotide polymorphism
TE: Terminal ear
UPGMA: Unweighted pair group method with arithmetic mean
VRN3: Vernalization 3
