# Supplementary material for: Natural variation in genes potentially involved in plant architecture and adaptation in switchgrass (Panicum virgatum L.)
Source: BMC Evol Biol. 2018 Jun 14;18:91. doi: 10.1186/s12862-018-1193-2 (PMC6000970; doi:10.1186/s12862-018-1193-2)

Additional file 1 for:

Natural variation in genes potentially involved in plant architecture and adaptation in switchgrass (*Panicum virgatum* L.)

Bochra A. Bahri[1,2*], Guillaume Daverdin[1,‡], Xiangyang Xu[1,‡], Jan-Fang Cheng[3], Kerrie W. Barry[3], E. Charles Brummer[4], Katrien M. Devos[1]

[1] Institute of Plant Breeding, Genetics and Genomics (Dept. of Crop and Soil Sciences), and Dept. of Plant Biology, University of Georgia, Athens, GA 30602, USA

[2] Laboratory of Bioaggressors and Integrated Protection in Agriculture, The National Agronomic Institute of Tunisia, University of Carthage, 43 Avenue Charles-Nicolle, Tunis 1082, Tunisia

[3] DOE Joint Genome Institute, Walnut Creek, California, CA 94598, USA

[4]Plant Breeding Center, Plant Sciences Department, University of California, Davis CA 95616, USA

[‡] Current address: GD: Vinson Edward Ltd, Faversham, ME13 8UP, UK; XX: USDA-ARS, Wheat, Peanut and Other Field Crops Research Unit, Stillwater, OK 74075, USA.

*Corresponding author, email bbahri@uga.edu ; bochraaminabahri@gmail.com

Table S1. List of switchgrass accessions used in the study with their ID and name, number of genotypes, ecotype identification, ploidy level, state of origin, and GPS coordinates

ID	Name	# of genotype	Ecotype[1]	Ploidy level[2]	Population type[3]	Location	Latitude	Longitude	Source
1	PI 315723	7	L	4x	Wild	Hoffman, NC	35,04	-79,56	GRIN
2	PI 315724	8	U	4x/6x/8x	Wild	Ellsworth, KS	38,73	-98,23	GRIN
3	PI 315725	2	U	4x	Wild	Coffeeville, MS	33,98	-89,68	GRIN
4	PI 315727	7	L	4x	Wild	Apex, NC	35,75	-78,84	GRIN
5	PI 315728	8	L	4x	Wild	Scotland County, NC (donated by Maryland)	34,79	-79,55	GRIN
6	PI 337553	7	U	8x	Wild	Rafaela Experiment Station, Argentina	-31,18	-61,55	GRIN
7	PI 414065	12	L	4x	Wild	Pangburn, AR	35,42	-91,84	GRIN
8	PI 414066	8	U	8x	Wild	Grenville, NM	36,74	-103,46	GRIN
9	PI 414067	9	U	8x	Wild	Soil Conservation Service, NC	35,84	-78,63	GRIN
10	PI 414068	9	U		Wild	Soil Conservation Service, KS	38,83	-97,62	GRIN
11	PI 414070	12	L	4x	Wild	Soil Conservation Service, KS	38,83	-97,62	GRIN
12	PI 421138	13	U	8x	SIC	Moore County, NC	35,34	-79,36	GRIN
13	PI 421520	13	U	4x/8x	SIC	Kay County, OK	36,8	-97,29	GRIN
14	PI 421521	8	L	4x	SIC	Wetumka, OK (developed in KS)	35,24	-96,24	GRIN
15	PI 421999	15	L	4x	Wild	Pangburn, AR	35,42	-91,84	GRIN
16	PI 422001	12	L	4x	Wild	Stuart, Martin County, FL	27,2	-80,25	GRIN
17	PI 422003	11	L	8x	Wild	FL	28,57	-82,38	GRIN
18	PI 422006	12	L	4x/8x	SIC	George West, TX	29,29	-98,73	GRIN
19	PI 422016	11	L	4x	Wild	FL	28,57	-82,38	GRIN
20	PI 431575	8	U	4x/8x	SIC	Raleigh County, WV (from KY)	37,79	-81,19	GRIN
21	PI 476290	14	L	4x	Wild	Wilmington, NC	33,92	-78,13	GRIN
22	PI 476291	8	L	4x	Wild	MD	39,04	-76,91	GRIN
23	PI 476292	14	U	4x/8x	Wild	Franklin County, AR	36,23	-91,75	GRIN
24	PI 476293	8	L	4x	Wild	Heislerville, NJ	39,25	-74,99	GRIN
25	PI 476294	12	U	4x/6x	Wild	Eads, CO	38,46	-102,65	GRIN
26	PI 476295	14	U		Wild	Colorado Springs, CO	38,79	-104,83	GRIN
27	PI 476296	15	U	4x	Wild	MD	39,04	-76,91	GRIN
28	PI 642190	14	U	4x	Bred	NM	34,78	-106,69	GRIN
29	PI 642191	10	U	4x	Bred	SD	44,08	-103,17	GRIN
30	Citrus Co-FL	7	L			Citrus County, FL	28,75	-82,53	Collected from public land[4]
31	HSP-FL	14	L		Wild	Hillsborough River State Park, FL	28,06	-82,3	Collected from public land
32	OSSP-FL	12	L	4x/8x	Wild	Oscar Scherer State Park, FL	27,18	-82,49	Collected from public land
33	Pasco Co-FL	7	L	4x/8x	Wild	Pasco County, FL	28,36	-82,21	Collected from public land
34	SNF	11	L		Wild	Sumter National Forest, SC	34,22	-82,16	Collected from public land
35	SPBluff	12	L	4x/8x	Wild	Sprewell Bluff, GA	32,91	-84,33	Collected from public land
36	SWFWMD-FL	4	L			Southwest Florida Water Management District, FL	28,58	-82,17	Collected from public land
AP13	AP13	1	L						
DS419	DS419	1	L						Christian Tobias, USDA-ARS
DS441	DS441	1	L						Christian Tobias, USDA-ARS
VS16	VS16	1	U						
	Total	372							

[1] Ecotype classification is based on morphology of plants grown until flowering in the greenhouse and presence/absence of the chloroplast trnL (UAA) intron deletion [35]; L and U refer to lowland and upland ecotype, respectively.
[2] Ploidy level is based on flow cytometry data from [114-116, 32, 35]. Some accessions have mixed ploidy levels
[3] Wild: prairie-remnant population; SIC: source-identified cultivar derived from a random seed increase without breeding within a prairie-remnant population; Bred: cultivar produced after one or more cycles of selection and breeding
[4] Appropriate permissions (permit number 10240710 from Florida Department of Environmental Protection) were obtained to make collections on public lands.

References (if not listed below please refer to the references in the main document):

114. Casler MD, Vogel KP, Harrison M. Switchgrass germplasm resources. Crop Sci. 2016;55(6):2463–78.

115. Missaoui AM: Molecular phylogenetic analysis, genetic mapping, and improvement of switchgrass (*Panicum virgatum* L.) for bioenergy and bioremediation to excess phosphorus in the soil. PhD dissertation. University of Georgia; 2003.
116. Triplett JK, Wang Y, Zhong J, Kellogg EA. Five nuclear loci resolve the polyploid history of switchgrass (*Panicum virgatum* L.) and relatives. PLoS One. 2012;7(6):e38702.

Table S2. Sequences and annealing temperatures of the 33 primer pairs used for PCR amplification of the selected 12 genes. Conserved regions in orthologous exons in *Oryza sativa* (rice), *Sorghum bicolor* (sorghum), *Zea mays* (maize) and *Setaria italica* (foxtail millet) were used for primer design

Gene code	Gene name	Primer name	Orientation	Primer sequence	Annealing Temperature	Region	GeneBank reference/species of origin
PhyB	Phytochrome B	PhyBF1	FORWARD	GTGTTCGAGCAATCGGGC	Tm59^{0}C	675-692	XM002467928.1/Sorghum
		PhyBR1	REVERSE	CTAATGATGACTGCCATAACAAGTGAT		1580-1606	
		PhyBF2	FORWARD	TTGTTATGGCAGTCATCATTAGC	Tm57^{0}C	1607-1585	
		PhyBR2	REVERSE	CTCTTAAAGCTCGTGAGAGTAGCTT		2616-2640	
		PhyBF3	FORWARD	GCTACTCTCACGAGCTTTAAGAGG	Tm59^{0}C	2616-2641	
		PhyBR3	REVERSE	ACCACCTTTTGTCCTGTGACAT		3474-3495	
		PhyBF4	FORWARD	GTCACAGGACAAAAGGTGGTC	Tm57^{0}C	3476-3496	
		PhyBR4	REVERSE	ACCCGTCCTCAATACTTTGG		4123-4142	
GI	Gigantea	GIF0SG	FORWARD	GGCCTATGTTGAGTACTTTGG	Tm55^{0}C	504-524	BK006299.1/Zea
		GIR0SG	REVERSE	TTGCTGTCTCATAACGAGCTA		1208-1228	
		GIF0	FORWARD	GGAGAATTGAAGCCTCCAAC	TD53-50^{0}C	1078-1097	
		GIR1b	REVERSE	TGCAGATGCATCCAATTCTT		1859-1880	
		GIF2	FORWARD	CCTGAAATTGTTGTAGCTGCTCC	Tm59^{0}C	1860-1882	
		GIR2	REVERSE	TTACTGGTGTAAGGGTCAGGTGAG		2786-2808	
		CG16	FORWARD	GAACAAGAATGGTTCAACCTCAC	Tm55^{0}C	2902-2921	
		CG16	REVERSE	GGCCATCAGCAACTGATAAAC		3481-3501	
FLD	Flowering Locus D	FLDF1b	FORWARD	ATAGTGATCAATCGGGAGCCTAC	TD62-55^{0}C	316-338	XM002448276.1/Sorghum
		FLDR1b	REVERSE	TGTCCCCTTCATAGACTTGCC		1348-1368	
		FLDF2c	FORWARD	CAGGTGGTTGTCAATGGAGG	TD62-55^{0}C	1330-1349	
		FLDR2	REVERSE	CCTTTCCTGCCAACTAATTTCAC		2362-2384	
FLT	Flowering Locus T	FLTF2b	FORWARD	CCGCAGACATATTCTTGTTATGATA	TD62-55^{0}C	591-615	EU241915.1/Zea
		FLTR2	REVERSE	AAGCAAATTAGTGGTACAGCTGTG		1473-1496	
		FLT3N	FORWARD	GTCGGCATGCACTGAAATA	TD62-55^{0}C	1449-1467	
		FLT3N	REVERSE	CCAGAGTGTAAAGGTTCCTCAT		2455-2476	
Hd1	Heading date	Hd1F2b	FORWARD	ACATCCAAAGCCAACCACAAG	Tm56^{0}C	970-990	EU302134.1/Zea
		Hd1R2b	REVERSE	GATGGAATCGGTGTAAGCACTG		2122-2143	
		Hd1F3	FORWARD	CAGTGCTTACACAGATTCCATCA	Tm57^{0}C	2122-2154	
		Hd1R3	REVERSE	AGAACATCTGGTCCACTTCGA		3079-3099	
TE	Terminal ear	TE1F2	FORWARD	ATCAGGAACATACCCAACAAGTACAG	Tm59^{0}C	1567-1592	AY741534.1/Sorghum
		TE1R2	REVERSE	TTCTCAACTTGCTTCGTGCAA		2632-2652	
		TE1F3	FORWARD	CACGAAGCAAGTTGAGAAAAGACCTATC	Tm63^{0}C	2635-2662	
		TE1R3	REVERSE	AGAACGCCACCGGCAGGTA		3246-3264	
PhytC	Phytochrome C	CG6	FORWARD	GAAAGGCAGGAAGCTGTGG	TD62-56^{0}C	1169-1188	XM002466396/Sorghum
		CG6	REVERSE	CCGTTCTCATCTGCATCGAC		1939-1958	
		CG7	FORWARD	TCAAGGCATTCTTGGAGGTG	TD62-56^{0}C	1936-1955	
		CG7	REVERSE	AGCTCACTGGGGTTCTTCAC		2659-2678	
		CG8	FORWARD	TCGGGACCTTTCAGACAAAG	TD62-56^{0}C	2428-2447	
		CG8	REVERSE	GCAACAAGTTGCCTCTGTTC		3194-3213	
		CG9	FORWARD	TCGCCAAGAATTAAGGAACC	TD60-54^{0}C	2993-3012	
		CG9	REVERSE	ACCGTTTGCTGCTGAGTTG		3692-3711	
Rht1	Gibberellin-insensitve (GAI) gene	Rht1.1	FORWARD	GCGCTCGGGTACAAGGTG	Tm61^{0}C	130-147	XM002466549.1/Sorghum
		Rht1.1	REVERSE	GAGGTAGGGGCAGGACTCGT		951-970	
		Rht1.2NF/R5	FORWARD	TACCTCAAGTTCGCCCACT	Tm57^{0}C	964-982	
		Rht1.2NF/R5	REVERSE	CTCACTGCAATTCAGCGTTC		1974-1994	
PGM	Phosphoglycerate mutase	PGM1	Forward	TGAATTATTGAGACTGGGAGA	TD57-50^{0}C	7808-7828	XM002467877/Sorghum
		PGM1	Reverse	ATGCAGTGAGACAGTCAAGTT		8823-8843	
		PGM2	Forward	AACTTGACTGTCTCACTGCATTT	TD62-55^{0}C	8823-8845	
		PGM2	Reverse	TTTGGCAGCTGCTCCTG		9862-9878	
		PGM3	Forward	CATCCGTGAGGACGTGGA	Tm57^{0}C	9807-9824	
		PGM3	Reverse	GTTGTCGCACCGTCACGTA		10961-10979	
Tb1	Teosinte branched 1	Tb1F1	FORWARD	CACGGTCTTTGGTTTCCTGA	TD53-50^{0}C	1779-1798	AF131655/Sorghum
		Tb1R1	REVERSE	ATTGGTCCGGCTTTGGG		2432-2448	
		Tb1F2	FORWARD	CATGGACTTACCGCTTTACCAA	Tm57^{0}C	2387-2408	
		Tb1R2	REVERSE	CCATTGTTCTTGGCAGTAGTAGTTG		3423-3399	
		Tb1F3	FORWARD	GCAACTACTACTGCCAAGAACAATG	TD53-50^{0}C	3398-3423	
		Tb1R3	REVERSE	TCATTCTTTCATCGAACAACTGAA		4100-4123	
		Tb1F4	FORWARD	CCTAGCCTTCGTAATTGACT	TD62-55^{0}C	3892-3910	
		Tb1R4	REVERSE	CACTTTAACCTGATTATGATGTT		4860 - 4882	
Dw3	Dwarf	Dw3.2F	FORWARD	AGAAGCAGCGCATCGCCAT	TD62-55^{0}C	4458-4476	XM002445621/Sorghum
		Dw3.2R	REVERSE	GGTCCCCCGAGAAGCCCT		5536-5553	
		Dw3.3bF	FORWARD	CACCGTGCTGCAGAAGATGTTC	Tm64^{0}C	5509-5530	
		Dw3.3bR	REVERSE	CGAGTGCGACCCCTGCTC		6650-6667	
Vrn3	Vernalization 3	Vrn3.1bF	FORWARD	AGAACATTGATTCAAAGAAAGTC	TD62-55^{0}C	449-481	XM002443233/Sorghum
		Vrn3.1bR	REVERSE	AATCTTCAATCTCATCTTTACACA		1665-1688	
		Vrn3.2F	FORWARD	GGTACTGGAATAGCAGAGAA	TD62-55^{0}C	1341-1322	
		Vrn3.2R	REVERSE	AGCTTAGTACAGAAACCAGTTC		2271-2292	

Table S3. Sequences of 56 regions of AP13 extracted from the Phytozome database (http://www.phytozome.net/), and used as reference for read mapping and SNP identification.

>Hd1_*sg0contig03275_4535-6757
AGAACATCTGATCCACTTCGGTGTCCATATCAGATCTTTTGGCGAAGCGGCCCTTGATCC
TTGGCCGTGCTTCTGCATATGTCTTCCTTGTTGCATAACGTATGGTCTTCTCAAACTTTC
TATTCTTCTTCTTCTCCTTGTACCTTAGGACCCTGGCCTCTCTGTCCATTGGGCTAAAGT
GAAGTGGCATCTGAAGTGAAGGACCTGAGAAGAGACTGATGGCTCCAGCAGGTGTCAGGA
CGCTGGAGTTTGCCATGTCTGTGGCCATGTTGTCTGGTACTATACCCACCTCCATTGATG
AGAAAGATATCTGAGATGGAATCGGCAAATGGTCACTGAAATATGCAAATCATAAGGGGA
TTGCAGGTGTCATAGACCCTGCAATGATATATAGATGGGTTTAATTTAAGTGGTTAATTC
TACACATGGAAAAAAATGCTTATTTACTGTTGCAAAATAAGAAACAAAATGTAGTGTAAC
TTTATTAGCAACGAAATTGGACAACTTAACTGTTATCTGAATAGATAGTTGGGTCCCTCC
CATTTCTTTGGTGTATTGATTGATTTTCATAAAAAGTGTGGAAAAATAGCTTTCCAATTA
TTTTTATATGAAATAAGATCTCCATCCATCGGTGATTAATATATTGAATCCACTTAGAGA
TAACATGAGTGGTCTATGTTACAAAGTGCAGGAATCTGTCAATAAGTATTCCTAAGGATC
CTTTTCTTATGACTGGCTTTTAGAGCAGTGGAGTCGTAGTATACATAAGCACATTTTTGC
TACTTCCCTTATCATCAGCCTAAGAATTGTCTGCCTACAATTAGAAAATAGTAGGAAAGG
AAACGAAAATGGAATAAAGTGAGAAATACCACCAGAGAACTAAAAGTTAGTTAATGCTTC
ATCTATTACTAAAATCTTTTTAAACCATCCACCAGTTCTTCTGAAACCTACCTTTTGTTG
AGATCAATATGATATACCCTTTTTCTAGATAATTATTTGGATATGGTCTTAAGTGGAAAG
TAACATGCTAACCCAAATTTGCAGGATCCACCCTAGAAAAAAAAACTTGGCATGTGACAG
CATAGTAGTAATAGATGAACTCACGCTGTTGCTGATGGAATCGGTGTAAGCACTGACCCC
GGCAGTTATGGAGGCAGCCTGCTCGGCCCCTACAACTCCATAGCCACTCTGCTGCTGCTC
TTTTACCATAGCAACTTGTGAAGGTACCACACACTCTCCAGCCTCCTTATCTCCATATTC
TTTTTGCATCAGCTGCTGCTGCTGCCGTTCTTGCATCCCATACTGCTCTGTGTTGTTGAT
GTGGTTGTCGCAGTATGAATTGTACCCAACAAGATCAAAGTATTCTTCCACTTCTGCAAA
GTACATGTTGTTGCTACTGATGTTGTTCCTGCACTGATGATGTTGTCTTtcttgtcgtca
gaatccttgctgagcagcagccaagAGTCCACCTCCTCTTCCTTGTCACCGATGGCCCCG
GTGGTGGCTGCTGCCTCGGCAAGCACAGAAGCAGCCGGAATGGCTGCAGCGGGGAGTGGC
ACCACCGGCACGCGTTGGTGCCTCCCGGCGAGCGGGTTTGCAGAGTGCACCTTGGCGTCA
CAGGCGGCACACAGCGCTGCCGCGTCGGCGCGGCACACCAGCACGGCGGGGGCACGCTCG
CATGCTTCGCAAACGCGCACACGCTCATGGCGTGAGGCCACACGGTTTGCGGCGTGTACC
CTCGTGTCGCACGATGCGCAGAGGTACGCAGCGTCAGCGCGGCAGTACACCACGCCCGGC
GCCGCGCGGCACCCGTCGCATGGCTTGGGCCATGGGCAACTCCCTTCGCCTCCTCCAACC
TCCTTCTCGAGGGCGTTTCTGCCAAAATTATAATTCATGAACGGAGAGATCTTGGCACTT
GCACCACATAGACAATCTAAGAGGCTCAGTCAGAGCAAGCTAGTAGTAGGATAGTCTAGA
TAGCAGTATACTGCTACCCTATACGTGGGTGTATATGTCTCCTCCCGTTCCTCTGCTTTC
CATGGCTCCTATGCCTTGTTGCAGTGACTGTGCGAGTATGTTGGGTTGGGGGAGTCACCA
ATGTCACGGGTTCAGTGTCGAGTACTAATAGCAGGGTGTGAGGAGAATCCTGGGAGTGAC
ACCTGGCTTCATTCTTGGGGCTGAGCATCGCTGTGTGCCTATCTTGTGGTTGGCTTTGTA
TGT
>Hd1_*sg0contig05584_7137-9276
AGAACATCTGGTCCACTTCGATTTCCATATCAGATCTTCTGGCGAAGCGGCCCTTGATCC

TTGGCCGTGCTTCTGCATATGTCTTCCTTGTTGCATAACGTATGGTCTTCTCAAACTTTC
TATTCTTCTTCTTCTCCTTGTACCTTAGGACCCTGGCCTCTCTGTCCATTGGGCTGAAGT
GAAGTGGCATCTGAAGTGAAGGACCTGAGAAGAGACTGATGGCTCCAGCATGTGTCAGGA
CGCTGGAGTTTGCCATGTCTGTGGCCATGTTGTCTGGTACTATACCCACCTCCATAGGCT
CCATTGATGAGAAAGATATCTGAGATGGAATCGGTAAATGGTCACTGAAACATGTGAATC
ATAAGGGGATTGCAGGTGTCATAGACCCTGCAATGATATATAGACGGGTTTAATTTAAGG
GGTTAATTCTACGTATGGAAAAAAAAGGTGATGTTATTTACTGTTGCAAAATAAGAGACA
AAATATAGTGTAGCTTTGTTAGCGACGAAATTGGACTACTTAACTGTTATCTGAATAGAT
AGTTGGGTCCCTCCCATTTCTTTTGTGTATTGATAGATTTTCAGAAAAAGTGTGGAAAAA
TAGCTTCGAATTATTTGTATATGAAATGAGGTCTCCATCAGTGATTAATGTATAGAATCC
ACTTAGGGATGACGTGAGTGGTCTATGTTACAAACTGCAGGAACGTATCAATATGTATTC
CTAGTATACGATAAGCACATTTTTGCTACTTCCCTTATCATCAGCCTAAGAATTGTCTGC
CTACAATTAGAAAATAGTAGAAAAGTAAACGAAATGGAAGAAAGTGAGAAATACCGCCAG
AGAACTAAAACTCCTTTTTCAGCAAGAAACCTACCTTTTGTTGTGATCAATTGATATGGC
CCTTTTTTCTAGATAAATGCTTTTATTTGGATATGGTCTTAACTGGAAAGTAACATGCTA
ACCCAAATTTGTAGGATCCACCCAAGAAAAAAAAACACATGACATGTGACAGCATAGTAA
TAGATGAACTCACGCTGTTGCTGATGGAATCGGTGTAAGCACTGACCACGGCAGTTATGG
AGGCAGCCTGCTCTGCCCCTACAACTCCATAGCCACTCTGCTGCTGCTCTTTTGCCATAG
CAACTTGTGAAGGTACCACACACTCTCCAGCCTCCTTATCTCCATATTCTTTTTGCATCA
GCTGCTGCTGCCGTTCTTGCATTCCGTACTGCTCTGTACTGTTGATGTGGTTGTCGCAGT
ATGAATTGTACCCAACAAGATCAAAGTATTCTTCCACTTCTGCAAAGTACAtgttgttgc
tactgatgttgtttctggtgctgatgtttttgttgttgtcgtcgtCAGAATCCTTGCTGA
GCAGCAGCCAAGAGTCCACCTCCTCTTCCTTGTCACCGATGGCCACGGGGGTGGCTGCTG
CCTCGGCAAGCACAGAAGCAGCCGGAATGGCTGCAGTGGGGAGTGGCACCACGGGCACGC
GCTGGTGCCTCCCAGCGAGCGGGTTTGCAGAGTGCACCTTCGCGTCGCAGGTGGCACACA
GCGCTGCTGCATCGGCGCGGCACACCAGCACGGCGGGGGCACACTCGCATGCCTCGCAGA
CGCGCACACGCTCATGGCGCGAGGCCACGCGGTTCGCGGCGTGCACCCTCGTGTCGCACG
ATGCGCAGAGGTACGCAGCGTCGGCGCGGCAGTACACCACGCTCGGCGCCGCGCGGCACC
CGTCGCACGGCCTGGGCCATGGGCAACTCCCTTCTCCTCCTCCTCCAACCTCCTTCTCGA
CGGCGTTGCCGTCAAAATTATAATTCATGAACGGAGAGATCTTGGCGCATGCACCACATA
GGCAATCTAAGGGGCTCAGAGCAAGCTATTAGTAGGAGTCTAGATAGTATTACTGCTACT
ATACGTGGGTGTATATGTATTCTCCCGTTCCTCTGCCTTTCATGGCTCCTATGCCTTCTT
GCAGTGACTGAGTATGTTGGGTTGGGGGAGTCACCAATGTCACAGGTTCAGCGTAGAGTA
CTAATAGCAGGGTGTGAGGAGAATCCTGGGAGTGACACCTGGCTTCATTCTCGGGGCTGA
GCGTCGCTGTGTGCCTATTCTTGTGGTTGGCTTTGTATGC
>Ma3_*sg0contig13571_3958-761
ACCCATCCTCAATACTTTGAAGACTGGCGTCCTTTACAATCTTGGACATCTGTTTCTCAC
AAGCAGAGCTAGTTTCAAGGAACTGCCTCTGGTCATCATTTAAATCAGTCATCTGCAACA
GAGAGTTGGTAAATCGGATACCACTAAGAGGATTCTTTATCTCCTGGCAAATATAGGCCG
ACTCTTTCATCCTTGCATAACACTTCTTTTCTTGTTGCCTCTGAATCTCAAGGGCTTGCT
GTAATTCGGCACTTGCAATCTGCAAGAAACAAAAGGCCCCAATGGACTTACCATCCATTT
TGCTCCTTGTGTTGGCGGTCAATAAGGACTGCACATACTTCCCATTCTTATCGAAAAATG
AAAAGGGGAACTTTTCATAATCCTGTCCCCCTATAGCATTGTGAAGGACAACCATGAACT
TTGTCAATGCATCTGGGCCCTTAAGTTGACAAATGTTTCCAAATACCTCTCCAATAAGCA

GCTTACCAATTACTTCACTTCTCGACCATCCTGTAAGTTTTTCCATAGCTGTGTTCCATT
CTGAACAACAAGTATTCTCATCTGATGCAAAAATTGGGGGTATTAGAGGATTTGGATTGT
GTACAATAGCCTTATAATCCCCTTGTATGTTGACAAATTTATCCATGGCCACCTTTTCTC
CTGTGACATCTTGTCCAACAAAACAGACACCAACAATATTTTTTGTGTAATCTCTACTGG
AACAAGCATTGACAATAACAAATATTGGTCCCTTCGATTGCTCTGGCCCAAATGTCTTCA
ACTTTATCTCCACATTTTTGTCTTCCTCACCTGTACGAGCAGCAAGTTCATAAATCAGGG
CTCACCTCTGGTACAGGTTTGATGCACATACTTTTCACATTTGAGCATGGCTGTCTACTA
GTTCTAAGCACTGGTTCTAAGATGCTTCGATGAAAATGGTTGAGAAGAATTAATAGTAGT
TTCTAGCTGGCCCCCATTTTCTATACTTATTCATGCTATGCACAAAAAATGAAGTAAGCG
TATGAAACTGAGTGCGTCTATTTTCAAGGAGTATATATATCATAGATTTCAATACTTGAT
ACAATAAATATGTATACATCATATTTATACACTTCTATGATTGTATATACATTTACAATT
CTTTTTTCCTTGACTGCAGTGGGTGATTCCAGCAGACATAATACAAAAGAAAGAGCGGTT
ATATAAGAAAGAGAAATGATATATACCTCTTAAAGCTCGTGACAGTAGCTTTTCAACTAT
CTCCTCAGATTCCTTGAAGATAAGATAATTTACTAGCGATTTTCCCATTGCCTCCTCAAC
TGAAAGGCCTGTCAACTCAGCAATCTTTGCATTCCAACCATTTATACATCCATCAGTATC
TACTGCAAATATAGGGACTGTTGCTGTCTCAATCAAGCGAACCATCTCTCTTGCTACGGA
GCTGAGCTCATTTATACCCCGCAATTCTAGCTCCCCAAGCTGAACTTGTCCATTGACAAT
GGCTTTTGAGTTACTAGTGCCCTCTGCAACATCTCTGAAGGAGTCGCGCAATATGAGTTG
CAAGGAATGTATTGCATCCATTTCTGCATTCTCCCATGGTAGGCTTCTGCTTTTAACTAC
TTCAAGAAATGCCTTGAATGATGATCGTGGGTGCATCCTCTGACCATCATCCTTATCCTC
TGGGTGATGCTTCGCACCACCCCATTTGATTTCTTTAGCGGTGTGTGACCGGAACCAAAA
CAAGTAATCACTTGGCGTAATATAGGCTACTGCCATGCCACATACAGCATCCCCTAATGC
AGCAGCACCATGGTAGCCTGCATCAGCCAGGCTATCTGTGCTGAGCCCAGTTGAGTCCCC
ATGACACACTGTCAACCACTCGATGATATCTTTAATTTGGGACTCAGTGGGGGTGACACC
CAATGGATAGTACTTCCCATGATAATAGAGTGCAGCGCCATCACACTTCACAAGATCCAT
GATGCTAGGGCTCTGTGTGACAATGCCAGTTGGTGAATCTCGCAGTAACATGTCACACAA
CAGAGTCTGTGTTCGCAAAATGTGCTTCTCCGACAGCTGATGTGCAAGCTGCAACTCCAT
ATTGAGCTGCAGCCCAAAAGCCTGCATGAGAAACTCACAAGCATACCTCAATGGAAAAGG
GATAAACCGTGGTGATGTATGGTGGCACACTACCAACCCCCACAACTTCATAGCCGACGA
GATGCCACCGCGTGCTGTTTGCTCATCATCACCACCACTGCTGATGATGACCGCCATAAC
AAGCGATGCTATGGAACCCATGTTTGCCATGTACTGCGCATGACACCCGTGCGGGGCGCG
CAGTGTAGAGCCAACCAAACACAGTGGCTGTGACAGCCCAGGATCTTGTATGACTCTCAC
CGGCGTGGCATGACAATCGGCAATCATTCTCACCCGGTTCTGCCGGAACAGGAAGCGGGA
TGCCTGGGGGATATCTGTGGCGGGATAATGCAACCCGAGGTAGGGCTCAAGGTTATTGAG
CCGGCACTCAGCAACAACTTCCCCGTGCTCGTCTTCATGGAACCTGTACACCATGACACG
GTCGTAACCCGTGAGCTCGCGGACGTGTTCCACGACTGTGTCGCAGAGCAGCTTGACATC
ACCGCCGGGCAGCGCCTGGAGGCGGGAGATGGCGCGGACAGCGAGCTTCTGGGACTGGAC
TGCACCGGCGATGGAGAGCGCGGGGTCCTCGGTGCGGGCGGGCTCGAGGTCGATGACGAC
GCCGACGTCGATGCGGTGGAGGATGGCGTAGAACGGCTTGGAAGAGACCCTGGAGTGGAT
CCATAGCGGGTTTAGCAGCGAGATCTCGCGCGCGGCGAAGGCGCGCTCCAGGAGGACTGC
GGACGAGGGGGAGAAGAGGAGGCGCGCGTCGGCACCCAGGGAGACAGGGGGCGGCGCCGC
GGAGTCGAGCGAGGGGACGGAGTGGTGCGGCGACAGGTCGAGCAGGTCGGCGGCGTTCTC
GGAGAAGGCGAGGAGGCGGAAGGAGGAGTCGTCGGCGACGGCGAGCGTGCAGCCGAAGGG
CTGGATGTGGCCGCCGCGCTGGATGCGGGAGAGGTAGGCGGCGATCTGCTGCTCGGAGGA

CGGGGTGGGCAGCGAATGCGCTCGCAGCGACTGGGAGTAGTCGAAGCTGCGGCCCGAGGC
GCCCGACTGCTCGAACAC
>Ma3_*sg0contig21054_5003-1810
ACCCGTCCTCAATACTTTGAAGACCGGCATCCTTTACAATCTTGGACATCTGTTTCTCAC
AAGCGGAGCTAGTTTCAAGGAACTGCCTCTGGTCATCATTTAAATCAGTCATCTGCAACA
GAGAGTTGGTAAATTGGATGCCACTAAGAGGATTCTTTATCTCCTGGCAAATATAGGCCA
ATTCTTTCATCATTGCATAACACTTCTTTTCTTGTTGTCTGTGAATCTCAAAGGCTTGCT
GTACTTCGGCACTTGCAATCTGCAAGAAACAAAAGGCCCCAATGGACTTACCATCCATAC
TGCTCCTTTTGTTGGCGGTCAATAAGGCATGCACATACTTTCCATTCTTATCGGAAAATG
AAAAGGGGAACTTCTCATAATCCTGTCCCCCTATAGCATTGTGAAGGACGACCATGAACT
TTGTCAATGCATCTGGGCCCTTAAGTTGACAAATATTTCCAAATACCTCTCCAATAAGCA
GCTTACCAATTACTTCATTTCTCGACCATCCTGTAAGTCTTTCCATAGCCGTGTTCCATT
CTGAACAAGAAGTATTCTCATCTGATGCAAAAATTGGGGGTATTAGAGGATTTGGATTGT
GTACAATAGCCTTATAATCCCCTTGTATGTTGACAAATTTATCCATGACCACCTTTTGCC
CTGTGACATCTTGTCCAACAAAACAGACACCAACAATATTTTTTGTGTAATCTCTACTGG
AACAAGCATTGACAATAACAAATATCGGTCCCTTCGATTGCTCTGGCCCAAATGTCTTCA
ACTTTATCTCCACATTTTTGTCTTCCTCACCTGTACGAGCAGCAAGTTCATAAATCGGGG
CTCATCTCTGGTACAAGTTTGACGCACTTACTTTTCACATTTGAGCATAGTTGTCAACTA
GTTCTAAGCACTAGTTTTAAGATGCTTCGATGAAAATGGTTGAGAAGATTTAATGGTAGT
TTCTAACAGGCCCCCATTTTCTATACTTATTCATGCTATGCACAAAAAACGAAGTAAGCC
TATGAAACCGAGTACATCTATTTTCAAGGAGTATATATATCATATATTTCGATACTTGAT
CCAATAAATATGTATACATCGTATTTATACACTTCTGTGATTTTATATACATCAATAAAT
ACAATTCTTTTCTTTGACTGCAGTGCAATTATAGCAGACATGATACAAAAGAAAGAGCGA
TTATATAAGAAAGACAAATGAGATATACCTCTTAAGGCTCGTGACAGTAGCCTTTTAACT
ATCTCCTCTGATTCCTGGAAGATAAGATCATTTATTAGTGATTTTCCCATTGCCTCCTCG
ACTGAAAGGCCTGTCAACTCAGCAACCTTTGCATTCCAACCATTTATACATCCATCAGTA
TCTACTGCAAATATAGGGACTGTTGCTGTCTCAATCAAGCGAACCATCTCTCTTGCTACG
GAACTAAGCTCATTTATACCCCGCAATTCTAGCTCCCGAAGCTGAACTTGTCCATCGACA
ATGGCTTTTGAGTTACTAGTGCCCTCTGCAACATCTCTGAAGGAGTCGCGCAATATGAGC
TGCAAGGAATGTATTGCATCCATTTCTGCATTCTCCCATGGTAGGCTTCTGCTTTTAACT
ACCTCAAGAAATGCCTTGAATGATGATCGTGGGTGCATCCTCTGACCATCATCCTTATCC
TCTGGGTGATGCTTCGCACCACCCCATTTGATTTCTTTAGCAGTGTGTGACCGGAACCAA
AACAAGTAGTCACTTGGCGTAATATAGGCTACTGCCATGCCACATACAGCATCCCCTAAT
GCAGCAGCACCATGGTAGCCTGCATCAGCCAGGCTATCTGTGCTGAGCCCTGTTGAGTCC
CCATGACACACTGTCAACCACTCGATGATATCTTTAATTTGGGACTCAGTAGGGGTGACA
CCCAATGGATAGTACTTTCCATGATAATAGAGTGCAGCGCCATCACACTTCACAAGATCC
ATGATGCTAGGGCTCTGTGTGACAATGCCAGTTGGTGAATCTCGCAGTAACATGTCACAC
AACAGAGTCTGTGTTCGCAAAATGTGCTTCTCTGACAGCTGGTGCGCAAGCTGCAACTCC
ATATTGAGCTGCAGCCCAAAAGCCTGCATGAGAAACTCACAAGCATACCTCAATGGAAAA
GGGATAAACCGTGGTGATGTATGGTGGCACACAACCAACCCCCACAACTTCATTGCCGAC
GAGATGCCGCCGCGTGCTGTTTGCTCATCGTCGCCACCACTGCTGATGATGACCGCCATA
ACAAGCGATGCTATGGAACCCATGTTCGCCATGTACTGTGCATGGCACCCGTGTGGGGCG
CGCAGTGTAGAGCCAACCAAACACAGTGGCTGTGACAGCCCAGGATCTTGTATGACTCTC
ACCGGCGTGGCATGACAATCGGCAATCATTCTCACCCGGTTCTGTCGGAAAAGGAAGCGG

GACGCCTGGGGGATATCTGTGGCGGGATAATGCAACCCGAGGTAGGGCTCAAGGTTATCG
CGCCGGCACTCAGCAACAACTTCCCCGTGCTCGTCTTCATGGAACCTGTACACCATGACA
CGGTCGTAACCCGTGAGCTCGCGGACGTGTTCCACGACTGTGTCGCAGAGCAGCTTGACA
TCCCCGCCGGGTAGCGCCTGGAGGCGGGAGATGGCGCGGACAGCAAGCTTCTGGGACTGG
ACTGCACCGGCGATGGAGAGCGCGGGGTCCTCAGTGCGGGCGGGCTCGAGGTCGATGACG
ACGCCGACGTCGATGCGGTGTAGGATGGCGTAGAACGGCTTGGAAGAGACCCTGGAGTGG
ATCCATAGCGGGTTTAGCAGCGAGATCTCGCGCGCGGCGAAGGCACGCTCCAGGAGGACT
GCTGACGAGGGGGAGAAGAGGAGGCGCGCGTCGGCACCCAGGGAGACAGGGGGCGGCGCC
GCGGAGTCGAGCGAGGGGACGGAGTGGTGCGGCGACAGGTCGAGCAGGTCGGCGGCGTTC
TCGGAGAAGGCGAGGAGGCGGAAAGAGGAGTCGTCGGCGACGGCGAGCGTGCAGCCGAAG
GGCTGGATGTGGCCGCCGCGCTGGATGCGGGAGAGGTAGGCGGCGATCTGCTGCTCGGAG
GACGGGGTGGGCGGCGCGCGCAGCGACTGGGAGTAGTCGAAGCTGCGACCCGAGGCGCCC
GACTGCTCGAACAC
>Ma3_sg0contig00216_6133-7266r
TATAAGTTGCAATGAGTGAATAGCATCCATCTCGTAGTCATTCCATGGCAAACTCTTCAT
CTTGACAACCTCTAGGAATGCCTTGAAGGACAACCTTGGGTGCATCCTTCTGTTGTCGTC
CTTGTCAGATGGATCATGCTTTGCAcCTCCCCATTTGATTTCAGCAGCTGTATGTGACCT
GAACCAGAAAAGAATATCCTTGGAAGTGATCTTAGCCACTGCCATTCCACAAATCATGTC
ACCAAGGGAGGCAGCTCCTGGATATCCAGCATCCTGGAGGCTGTCAGTACTCAAGCCAGT
GGAATCCCTATGAACTTCTGAAAGCCAGAAGGCAATATCACGTATCTGAGACTCAGTTGG
AGCTGTTTGAAGACGCCATACTTTGCCCCCATACaAAAGAGCAGCACCATCACATCTAAC
TAGGTCCATAATATTTGGACTCCCAGATATGATACTCAAGGGAGATGCTTCCCTGAATAG
CATGTCAGAGAGCATTGTTTGCATTCGTAGAATGCTTTTCTCTTGTATCTGCTTCTCCAA
TTCGAACTCCTTGTTTACATGGACAGCAAACACTTGTGCTAAGAATTCACAGGCATACCT
CAGTGGAAACGGGACATATCTGGGGCTCTCATGATGACAAACAAGGAGACCCCACAGCCT
CTTCTTCTTCTGCTGCTGTGATTGTTGTTCAGGATTGGGTTCATCATCCTCTTCATTTTC
ATTGACCACAACAGCCATGACAAGGGATGCAATTGAGTTCATGTTCTCCATATACTGAAG
GTGACAACTGTGTGGTGCTCTAAGAGTTGAACCACACAAGCTAATATCAATGGAGAGTGC
TTCATCTTCAATAATTTTCACAGATCTTGCACGACAATCACAAATCATCCGTACTTTGTT
CTTCATGAAAAGGAACCTTGCAGCTTGAGGAATATCAGTGGCCGGATAGTGAAGGCCAAG
ATAAGGCTCAATACCAGGTTTTGTGATCTCCGCAAAGACCTCCCCATGCTCATCTTCATG
GAACTTGTAAGCCATCACCCTGTCATAACCTGTAAGGTCGAAGATTTCCTTAACCACAGT
ATTGCATAAGGCCTCCATGCTCCCACCTGGTAGTGACTGGATTTTGGAGATTGC
>Ma3_sg0contig06603_6712-7842
TATAAGTTGCAATGAGTGAATAGCATCCATCTCGTAGTCATTCCATGGCAAACTCTTCAT
CTTGACAACCTCAAGGAATGCCTTGAAGGACAACCTTGGGTGCATCCTTCTGTTGTCGTC
CTTGTCAGATGGATCATGCTTTGCACCTCCCCATTTGATTTCAGCAGCTGTATGTGACCT
GAACCAGAAAAGAATATCCTTGGAAGTGATCTTAGCCACTGCCATTCCACAAATCATGTC
ACCAAGGGAGGCAGCTCCTGGATATCCAGCATCCTGGAGGCTGTCAGTACTCAAGCCAGT
GGAATCCCTATGAACTTCTGAAAGCCAAAAGGCAATATCACGTATCTGAGACTCAGTTGG
AGCCGTTTGAAGACGCCATACTTTGTCCCCATACAAAAGAGCAGCACCATCACATCTAAC
TAGGTCCATGATATTTGGACTCCCAGATATGATACTCAAGGGAGATGCTTCCCTGAATAG
CATGTCAGAGAGCATTGTTTGCATACGTAGAATGCTTTTCTCTTGTATCTGCTTCTCCAA
TTCGAACTCCTTGTTTACATGGACAGCAAACACTTGTGCTAAGAATTCACAGGCATACCT

CAGTGGAAACGGGACATATCTGGGGCTCTCATGATGACAAACAAGGAGACCCCACAGCTT
CTTCTGCTGCTGCTGTGATTGTTGTTCAGAATTGGGTTCATCATCCTCTTCATTTTCATT
GACCACAACAGCCATGACAAGGGATGCAATTGAGTTCATGTTCTCCATATATTGAAGGTG
ACAACTGTGTGGTGCTCTAAGAGTTGAACCACACAAGCTAATATCAATGGAGAGTGCTTC
ATCTTCAATAATTTTCACAGATCTTGCACGACAATCACAGATCATTCGTACTTTGTTCTT
CATGAAAAGGAACCTTGCAGCTTGAGGAATATCAGTGGCTGGATAGTGAAGGCCAAGATA
AGGCTCAATACCAGGTTTTGTAATCTCCGCAAAGACCTCCCCATGCTCATCTTCATGGAA
CTTGTAAGCCATCACCCTGTCGTAACCTGTAAGGTCGAAGATTTCCTTAACCACGGTATT
GCATAAGGCCTCCATGCTCCCACCTGGTAGTGACTGGATTTTGGAGATTGC
>Ma3_sg0contig03093_10032-9091
GGCTGCTGGTCACTCCCAGTATCCCCATCCTCCTCTGCATCCTCGTTTATAGTTATTGAC
ATCACAAGCGACGCAACAGAGCCCATGTTTGCCATGTATTGCGTATGGCAACCATGGGGA
GCCCGAAGCGTGGAACCACATAGGCTGAGAGGCTGTGCTAGGCTATCATCCTGAATGATC
TTAACTGGAGTGGCAGAGCAATCGTATATCATCCGCACTTTGTTCTTCATAAACATAAAT
CTGGACGCCTGCGGGATGTCCGTGGCTGGGTAGTGCAGGCCAAGATATGGTTCTAGATCA
GATCTCCTGCACTCGGCGATAACCTCGCCATGCTCATCCTCATGGAACTTGTACGCCATG
ACCCTGTCATAGCCCGTGAGCTCGCTCACCTCACGGACAAGCACATCGCACAGCAGCGAA
AGGTTCCCGCTGGGCAGCGACTGCAGCCTGGAGATAGCCTTGGCGGCGAGCTTGTACGAC
TTGAGCGATCCCGCGGCGGTGACTGGCACCTCGGCGGGATTGACCGGCTCGAGATCGATG
ACGAGGCCAACGTCGATGCGGTGCATAATGGCGTAGAAGGGCTTCCCCGACGTCCTGGCA
TGCACGAGGATGGGGTTGAGCAGGTTGACCTCCCCGAAGGAGGCAGCCTTGTGCAGCGCG
ACGGAGCTCTGCGAGCGGAAGAGCATGCGCACGTCGGCGCCGATGGCGAGCGCATCCCGC
TGGTCGATGGTGGGGACTGCGTGCGGCGTGAGGTCGAGCATCTCGGGCGCGTTCCCGCTG
TAGGCGAGCAGCGCGAAGGTGTCCGGGTGGACGGCGAGCAGGCAGCCGAAGGGCTGGATG
AAGCGGCCCCGCTGCATGTTCTGGAGGTAGGTGGAGACGGTGCTGGTGCTGGTGCTGGCG
GACGGGCGGTTGGCCGCGCTCACCGAGGAGGAGTAGTCGAAG
>Ma3_sg0contig00846_10377-11312
GGCTGCTGGTCACTCCCAGTATCCCCATCCTCCTCATCATCCTCGTTTATAGTTACTGAC
AACACAAGTGACGCAACAGAGCCCATGTTTGCCATGTATTGCGCATGGCAACCATGGGGA
GCCCGAAGCGTGGAACCACATAGGCTGAGAGGCTGTGCTAGGCTATCATCCTGAATGATC
TTAACTGGAGTGGCAGAGCAATCGCATATCATCCGCACCTTGTTCTTCATAAACAGAAAC
CTGGACGCCTGCGGGATGTCAGTGGCTGGGTAGTGCACGCCAAGATATGGTTCTAGATCA
GATCTCCTGCTCTCAGCGATAACCTCACCATGCTCATCCTCATGGAACTTGTACGCCATG
ACCCTGTCATAGCCCGTGAGCTCGCTCACCTCACGGACAAGCACATTGCACAGCAGCGAG
AGGTTCCCGCTGGGCAGCGACTGCAGCCTGGAGATAGCCTTGGCGGCGAGCTTGTAGGAC
TTGAGCGCTCCCGCGGCGGTGACTGGCACGTCGGCGGGGTTGACCGGCTCGAGATCGATG
ACGAGGCCGACGTCGATGCGGTGCATAATGGCGTAGAAGGGcTTCCCCGACGTCCTGGCG
TGCACGAGGATGGGGTTGAGCAGGTTGACCTCCCCGAAGATGGCGGCCTTGTGCAGCGCG
ACGGAGCTCTGCGTGCGGAAGAGCGTGCGCACGTCGGTGCCGAGGGCGAGCGCGTCCCGC
TGGTCGATGGTGGGGACCGCGTGCGGCGTGAGGTCGAGCATCTCGGGCGCGTTCTCGCTG
TAGGCGAGCAGCGCGAAGGTGTCCGGGTGGACGGCGAGCAGGCAGCCGAAGGGCTGGATG
AAGCGGCCCCGCTGCAAGTTCTGGAGGTAGGCGGAGACGgTGCTGGTGCTGGCGGACGGG
CGGTTGGCCGCGCTCACCGAGGAGGAGTAGTCGAAG
>PAL_sg0contig282191_1-1143

gcagctccttcatgaagcacgccaaggaggtgaacgccatggacccgctcctcaagccca
agcaggaccggTacGCGCtCCGCACGTCGCCGCAGTGGCTGGGCCCCCAGATCGAGGTCA
TCCGCGCCGCCACCAaGTCCATCGAGCGCGAGGTCAaCtCGGTCAACGaCAaCCCcGTCA
TCGACGTCCACCGCGGCAAGGCGCTCCACGGCGgCAACTtCCAGGGCACCCCCATCGGCG
TGTCCATGGACAACGCCCGCCTCGCCATCGCCAACATCGGCAAGCTCATGTTCGCCCAGT
TCTCCGAGCTGGTgAACGAGTTCTACAACAACGGGCTgaCCTCCAACCTGGCCGGCAGCC
GcAACCCCAGCCTGGACTACGGCTTCAAGGGCACCGAGATCGCCATGGCGTCCTACTGCT
CCGAGCTCCAGTACCTGGCCAACCCgATCACCAACCACGTGCAGAGCGCGgAGCAGCACA
ACCAGGACGTCAACTCCCTCGGCCTCGTCTCCtCCAGGAAGACCGCCGAGGctGTGgACA
TCCTCAAGCTCATGTcgTCCACCTACATCGTCGCgCtCTGCCAGGCCGTCGACCTGCGCC
ACCTCGAGgAGAACATCAAGACCTCCGTCAaGAACACCGTCACCCAGGTCGCCAAGAAGG
TGCTCACCATGGACCCCACCGGCGAtcTCTCCGCCGCGCGCTTCAGCGAGAAGGACCTCC
TCACCGCCATCGACCGCGAGGCCGTCTTCACCTACGCCGAGGACGCCGCCAGCGCCAGCC
TCCCGCTCATGCAGAAGCTGCGCGCCGTGCTGGTGGACCACGCCCTCAGCAGCGGCGACG
CCGAGCGCGAGCCCTCGGTGTTCTCCAAGATCACCAAGTTCGAGGAGGAGCTGCGCGCCG
TGCTGCCACGGGAGGTGGAGGCCGCCCGCGTAGCCGTCGCCGAGGGCGCCGCGCCCCTGC
CCAACCGGATCAAGGACAGCAGGTCCTTCCCGCTCTACCGCTTCGTCCGCGAGGAGCTCG
GCTGCGTGTTCCTCACCGGCGAGAAGCTCAAGTCCCCCGGCGAGGAGTGCAACAAGGTCT
TCGTCGGCATCAGCGAGGGCAAGCTCGTCGACCCCATGCTCGAGTGCCTCAAGGAGTGGG
ACG
>PGM_sg0contig17299_6038-9157
GTTGTCGCACCGTCACGTAGGAGCTGTTCCACAGGGCGGGCGCGGCGCGGATCGgAGGCT
CTTGACAAGAAAATCAATGGAGCTTTACACATGTGCACCTCTGGAACTGCCACTTCGGTT
CGCGCCGAGACCCTTCCAGAGCAAAGAAAGCCGGCCCGGGCCCCGGCCACCAGCTCAAAA
ACCCCGCGAGCGATCGAGCCAGCAGCCCAGCACCGAGCAGACGCCAGAGCCCGGACAGAA
CTTTCAGCTCACCCATCAGCCGTGTCACCTGATCGCTTCTGGAAGGCCGACCGCGTCGGG
CATGGCGACGAGCCCGCCGGCGgAGGGGAAGCCGTGGGCGCTGGCGGCGCACCCGCGGCT
GGCCAAGGGGACGGTGGTGGGCGTGGTGGTGCTGGACGGCTGGGGCGAGGCGCCGCCCGA
CCCCTTCAACTGCATCCACGCCGCCGACACGCCCACGCTCGACGCCCTCAAGAAGGTACG
CTCACTCAGGCTGGGGCTGTCGGCGTTGCCGCcGGATTGCGTGTTCGCTGATGAACTCTG
CCTGCGTCGTGCGCGATGCCATGTGCCAGGCAGCTCCGGCGAGGTGGAGGCTCATCAAGG
CGCACGGCACGGCGGTGGGCCTGCCGACGGATGACGACATGGGCAAtAGCGAGGTCGGCC
ACAACGCGCTCGGGGCGGGCCAGATATACGCCCAGGGGTAAACGCTCTCTGCTCTCCGGT
TTCGTTTCGTTCTACTTGCGATCACCGGCCGGCGGCCGCCATATGGCCGGCCATTCGAGA
AGCAACCGgCCGGCGATCAGCTGACGTCGGCCCACGACGGCGCAGGGCGAAGCTGGTGGA
TCTGGCGCTCGCCTCCGGGAAGATATACGACGGGGAGGGCTTCAAGTACATCCGGCAGTC
CTTCGACGACGGCACCCTGCACCTCATCGGCCTGCTCAGCGACGGCGGCGTGCACTCGAG
GTTCGACCAGCTGCAGGTTACGTTGCTTGCTGCTACCGCTCGCTCCTGCTGACAGGTTCC
AGAGATCGACGTCGCCAAACTGGCTGGTCGCGACAGACAGCTGACGGTCACCGTCTGCCG
CCGTGTTTTTtCTTTtCCTTTTTtGGCAGCTGCTCCTGAAAGGGGCCAGCGAGCACGGGG
CGAAGAGGATACGCGTCCACGTCCTCACGGATGGCCGCGACGTGCTGGACGGCAGCAGCG
TCAGGTTCGTGGAGATGCTGGAGGAGGACCTCGGGAGGCTGCGGGACAAGGGCGTTGACG
CGAGGGTCGCGTCTGGCGGAGGTAGGATGTACGTCACAATGGATCGTTACGAGGTAATGC
TACCACTATTTGTCAAGGTGTTTcGATTTGGATCGTTGGGTTGCTAGCTACTAGGAGTAA

AACATCCTGAATTTTGTGAACCATGTATCCAATTTTGTGAGAATTTTAGTTTTCGTAATA
ATCGTGACAGGATTGAATTCGAATTCACCTAGAAGAAACAATCTGCAGAATATGAGAACA
GCTGCTACATTGTATTTGCCAGAAATCAGTTCTGAAAAACGAGTGCAGCTCAGCTACTCT
GCATTTCTTGATTCGTAACACCACTGAAGTgACCTGTCTTCTTTCGCAGAATGACTGGCA
GGTCGTGAAGCGGGGCTGGGATGCGCATGTCCTTGGCGAAGCCCCGCACAAGTTCAAGAA
CGCTCTTGAGGCTGTGAAAAAGCTCAGGGAGGATCCAAAGGCCAACGACCAGTATCTACC
CCCCTTCGTTATAGTTGATGAAAACGGAAAACCTGTCGGCCCGATACAGGACGGAGATGC
TGTCGTGACGTTCAATTTCAGAGCTGACCGGATGGTGATGCTTGCAAAGGCATTGGAGTA
TGAGGACTTTGGCAAATTTGATCGAGTTAGGGTCCCCAAGATACGTTATGCTGGAaTGCT
TCAGTACGACGGTGAGCTAAAACTTCCGAGCCATTACCTTGTTGCTCCCCCAGAGATAGA
GAGGACATCTGGAGAATACTTAGCCCGTAATGGCGTTCGCACCTACGCTTGCAGGTAATT
tGTTGTGGTTTATCTTGTGAGAAATCTTTGATCTGAAAATCTGAATGTTACATTACTTGT
GCATTGATTGCTCATATCTTTTCTTTGTGAAAAAAAAaTGCAGTGAGACAGTCAAGTTTG
GTCATGTCACCTTTTTCTGGAATGGAAATCGATCTGGTTACTTCAACCCAAGTTTGGAGA
AATATGAAGAAATTCCTAGCGACATCGGCATCCCCTTCAATGTTCAGCCGAAAATGAAGG
CCTTGGAAATTGCACACAAGGCAAGGGATGCCATCCTGAGCCGAAAATTTGATCAGGTAG
TTCTTGGGTTAATATCTTAAAAAGAATAGCTTGGAACACTGAACACAAATGTAACTTATA
GTTATAAGCCAGGGAGTTCAAACACTAATAAGGACATCTTGTAGGTGAGGGTCAACATTG
CAAATGGAGATATGGTTGGCCATACAGGAGACATTGAGGCAACTATAGTTGGATGCAAGG
CAGCCGATGAGGCTGTTAAGGTACATGCGGACTATCATCTTTGCAATCACATACCTATCT
TCCTGAGATTCCATAGTGAATGCTGTCCTGTTGTGAATTTGGTGCTTACGATAACTGCAA
TGCGAATTCTATCTCGTAGATCATCCTCGATGCAATTGAGCAAGTGGGTGGCATCTTTGT
ACTCACCGCCGACCATGGCAACGCCGAGGACATGGCGAAGAGAGATAAATCTGGAAAACC
ACTCCTTGACAAGGACGGGAAGGTCCAGACCCTTACTTCACACACGCTGAATCCGGTAGG
TGCCCGGCACAAGTCTCAGGATGCGTTAATCCATATATGTCCCGGCCTGCCTGTGTGGCC
TTAACTGAGAGCCTGAAACCCTCTGCAGGTTCCAATCGCCATAGGAGGACCTGGGCTTGC
CCCTGGGGTCAGGTTCCGGACGGACCTCCCAAACGCCGGCCTCGCTAATGTTGCGGCGAC
CGTCATGAACCTTCATGGGTTCCAGGCCCCTGATCACTATGAACCAACACTCATCGAGGT
CGTCGACAAGTGATGGTCCCAGTCTTTTTCAGCTATAGCTCTCCCAGTCTCAATAATTCA
>PGM_sg0contig200892_1560-2255r
ggttggccatacaggagacatttgaggctaactagttagttggatgcaaggcagccgatg
aggcttgtaaaggtcatgcgtactatcatctttgcaatcacatacctatcttcctgagat
tccatagtgaatgctgtcctgttgtagacttgtagatcatcctagtctttcactcctgcg
cataaaattttccagttcccacttttgaatttggtgcttacgataActgCAatgcgaatt
ctatctcgtagATCATCCTCGATGCAATTGAGCAAGTgGGTGGCATCTTCGTACTCAcCG
CCGACCATGGCAACGCCGAGGACATGGCGAAGAGAGATAAATCTGGAAAACCACTCCTTG
ACAAGGACgGGAAGGTCCAGACCCTTACTTCACACACGCTGAATCCGGTAGGTGCACGGC
ATTGATCCATATATGTCCCGGCCTGCTGGTGTGGCCTTAACTGAGAGCCTGAAACCCTGT
GCAGGTTCCAATCGCCATAGGAGGACCTGGGCTTGCCCCTGGGGTCAGGTTCCGGACGGA
CCTCCCAAACGCCGGCCTCGCTAATGTTGCGGCGACCGTCATGAACCTTCATGGATTCCA
GGCCCCTGATCACTATGAGCCAACACTCATCGAGGTCGTCGATAAGTGATGGTCCCAGTC
TTTTGCAGCTATAGCTCTCCCAGTCTCAATAATTCA
>PGM_sg0contig181405_20-2260
tgaaccgtgatccaatattgtgagaattttagttaccatgataattgtgataggactgag

atttcacctagaagaaacaatctgcagaatacgagaaccgctgctgcaTTGtATTGGCca
GAAAtCAGTtCTGAAAAaTGagtGCaGCTCAGCTActCTGCATTtGTtGATtCGtAcACc
GcTGAAgTAGCcTGTcTtcTTtcGcAGAaTGACTGGCAAGTcGtGAagCGGggTTGGgAT
GCGCATgtCCTTGGCGAAGCCCCacACAAGTtCAAgAGCGCTCTTGAGGCTGTGAAAAAG
CTCAGGgAGgATCCAAaGGCCAACGACCAGtATCTACCCCCcTTCGTTATAGTTGATGAA
aGCGGAAAACCGGTcGGCCCGATACAGgACGGGGATGCTGTCGTGACGTTCAATTTCAGA
GCTGACCGGATGGTGATGCTTGCAAAGGCATTGGAGTACGAGAACTTTGACAAAATTGAT
CGAGTTAGGTTCCCCAAGATACGCTATGCTGGTATGCTTCAGTACGAcGGCGAGCtAAAA
CTTCCGAGCCATTACCTTGTTGCTCCCCCAGAGATAGAGAGGACATCCGGAGAATACTTA
GCCCGTAATGgCGTTCGCACCTACGCTTGCAGGTAAATTTCTGGCCTTGTTTAGTTGTCT
CGTGTAAAGTTTTTGAAAAGAAATCTTTCTACATTTGAAGTACTAAACATAGACTAATCA
CAAAATTAATTACAAAACTCGTCTTTAATCACGAGACGAATCTAACGAGACGAATCTAAT
GAGCCTAATTAATCCGTCATTAAAGATTGTACTGTAGCATTACTGTAGTAATTTAGCATC
TAATTACGGTCTAATTAGGTTCATTAGATTCGTCTCGCGATTTACAGACAAACTATGCAA
TACGTTTTTtATTTtATCTAGATTTAAGTTTCCATGCAGGTGTCAGAAAAAaTATTTAGA
ATTTTGAATTATGCAACTAAACAAAGGCTCTGTGGTTTTTCTTGTGAGAAGTCTTTAATC
TGAAGGTCTGAATGTTACACTACTTGTGCATTGCTTGCTCATGTCTTTTCTATGTGAAAA
AAAATGCAGTGAGACAGTCAAGTTTGATCATGTCACCTTTTTCTGGAACAGAAATCGATC
TGGTTACTTCAACCCAAGTTTGGAGAAATATGAAGAAATTCCTAGCGACATAGGCATCCC
CTTCAATGTTCAGCCGAAAATGAAGGCCTTGGAAATTGCACAGAAGGCAAGGGATGCCAT
CCTGAGCCGAAAATTTGATCAGGTAGTtCTTGGGTTAATATCTTCAAAGAAAAGAGCTTG
GAACACTGAACACAAATGTAACTTATAGTTAAGCCAGGGAGTTCAAACACTAATAAGGAC
ATCTTGTAGGTGAGGGTCAACATTGCAAATGGAGATATGGTTGGCCATACAGGAGACATT
GAGGCAACTATAGTTGGATGCAAGGCAGCCGACGAGGCTGTTAAGGTACATGCGCATCAT
CTTTGCAATCCCCTAATCAATCTTACTGAGATTCCATAGTGATTGCTATCCTGTTGTAGA
TCATCCTCGATGCAATTGAGCAAGTGGGTGGCATCTTCGCACTCACCGCCGACCACGACA
ACGCCGAGGACATGGCGAAGAGAGATAAATCTGGAAAACCACTCCGTGACAAGGACGGGA
AGGTCCAGACCCTTAATTCACACACGCTGAATCCGTTAAGTGCACGGCACACGTCTCGGG
ATGCGTTAATCCATATATATGTCCCGGCCTGTCGGTGTGGTCTTAGGCTGTGTTTATTTG
GTGAAAAAATTTGGGGTTTTGGTACTGTAGCATATTTCGTTGTTATTTGACAAATAATAT
TTAATTATGGAATAATTAGGCTTAAAAGATTCAGCTCGTGATAATCAATTAGACTATATA
ATTAGTTATTTTTTtCAACtGCATTCATGTATGTGTACAAAAATTCGATGTGACGGATAC
TTTAGAAAAaTTTTTGGGAACTAAacACCCCcTTGACTGAGAGCCTGAAACCATCTGCAG
GTTCCAATCGCCATAGGAGGACCTGGGCTTGCCCCTGGGGTCAGGTTCCGgACGGACCTC
CCAAaCGCCGGCCTCGCTAATGTTGCGGCGACCGTCATGAACCTTCATGGCTTCCAGGCC
CcTGATCACTATGAGCCAaCACTCATCGaggtcgtcgacaagtgattgtcccaatctttt
gcaggtagctcccagtctcaa
>PGM_sg0contig191126_72-2290
tGAACCGTGTATCcAATATTGTGAGAATTTTaGTtACCATGATAATtGTGATAGgACTgA
GATTTCACCTAGAAGAAaCAATCTGCAGAATACgAGAaCCGCTGCTGCATTGTATtGgCC
AGAAaTCAGTtCTGAAAAATGAGTGCAGCTCAGCTACTCTGCATTtGTtGATTCGTAcAC
cGCTGAAGTAaCCtGTCTTtCTTtCGCAGAaTGACTGGCAGGTCGTGAAGCGGGgTTGGGA
TGCGCATGTCCTTGGCGAAGCCCCACACAAGTTCAAGAGCGCTCTTGAGGCTGTGAAAAA
ACTCAGGGAGGATCCAAAGGCCAACGACCAGTATCTACCCcAcTTCGTTATAGTTGATGA

AAGCGGAAAACCGGTCGGCCCGATACAGGACGGGGATGCTGTCGTGACGTTCAATTTCAG
AGCTGACCGGATGGTGATGCTTGCAAAGGCATTGGAATACGAGAACTTTGACAAATTTGA
TCGGAGTTAGGTTCCCCAAGATACGCTATGCTGGTATGCTTCAGTACGACGGCGAGCTAA
AACTTCCGAGCCATTACCTTGTTGCTCCCCCAGAGATAGAGAGGACATCTGGAGAATACT
TAGCCCGTAATGGCGTTCGCACCTACGCTTGCAGGTAAATTTCTGTCTTGTTTAGTTGCC
TCGTGTAAAGTTTTTGAAAAAAAAATCTTTCTACTTTTGAAGTACTAAACATAGACTAAT
CACAAAATTAATTACAGAACTCGTCTTTAAATCGCGAGACGAATCTAATGAGCCTAATTA
ATCTATCATTAGAGATTATTTACTGTAGCATTACTGTAGCAATTTAGCGTATAATTAGAT
TCATTAGATTCATCTCGCGATTTACAGACAAACTGTGCAATGCGTTTTTTATTTTATCTA
GATTTAATTCTCCATGCAGGTGCCGGAAAAAaTATTTAGATTTTTGAATTATGCAACTAA
AAAGGGCTCTGTGGTTTTTCTTGTGAGAAGTCTTTGATCTGAAGGTCTGAATGTTACATT
ACTTGTGCATTGCTTGCTCATGTCTTTTCTATGTGAAAAAAAAaTTGCAGTGAGACAGTC
AAGTTTGGTCATGTCACCTTTTTcTTGGAACGGAAATCGATCTGGTTACTTCAACCCAAG
TTTGGAGAAATATGAAGAAATTCCTAGCGACATAGGCATCCCCTTCAATGTTCAGCCGAA
AATGAAGGCCTTGGAAATTGCACAGAAGGCAAGGGATGCCATCCTGAGCCGAAAATTTGA
TCAGGTAGTTCTTGGGTTAATATCTTCAAAGAAAAGAGCTTGGAACACTGAACACAAATG
TAACTTATAGTTAAGCCAGGGAGTTCAAACACTAATAAGGACATCTTGTAGGTGAGGGTC
AACATTGCAAATGAAGATATGGTTGGCCACACAGGAGACATTGAGGCAACTATAGTTGGA
TGCAAGGCAGCCGACGAGGCTGTTAAGGTACATGCGCATCATCTTTGCAATCCCCTAATC
TATCTTACTGAGATTCCATAGTGATTGCTATCCTGTTGTAGATCATCCTCGATGCAATTG
AGCAAGTGGGTGGCATCTTCGCACTCACCGCCGACCACGGCAACGCCGAGGACATGGCGA
AGAGAGATAAATCTGGAAAACCACTCCGTGACAAGGACGGGAAGGTCCAGACCCTTAATT
CACACACGCTGAATCCGGTAAGTGCACGGCACACGTCTCGGGATGCGTTAATCCATATAT
ATGTCCCGGTCTGTCGGTGTGGTCTTAGGGTGTGTTTATTtGGTGAAAAAaTTGGGTTTT
TGGTACTGTAGCATATTTCGTTGTTATTTGACAAATAATATTTAATTATGGACTAATTAG
GTTTAAAAGATTCAACTCGTGATAATTAATTAGACTATATAATTAGTTATTTTTtCAACT
GCATTCATGTATGTGTACAAAAATTCGATGTGACGGATACTTTAGAAAAAaTTTtGGGaa
CTAAaCACCCCcTTGACTGaGCCTGAAaCCaTCTGCAGGTTCCAaTCGCCATAGGAGgAC
CTGGGCTTGCCCCTGGGGTCaGGTTCCGGACGGACCTCCCAAaCGCcGGCCTCGCTAAtG
TTGCGGCgACCGTCATGAACCTTCATGGCTtCcAGgCCCCTGATCACTATgAGCCAAcAC
TCaTCGaGgtCGTCGaCAAgTGaTtGTCCCAatCTTttgcaggtacctcccagtctcaa
>PGM_sg0contig320997_sg0contig338773_1-293_1-1319r
agctggcggcgcacccgcggctgccaaggggaCGGTGGTGGCCGTGGTGGTGCTgGACGG
CTGGGCgaggcgCCGcCCGACCCCTTCtaCTGCATCCACGCCGCCGACAcGCCCACGCTC
GACGcCCTCAAGAAGGTACGCGGCCCACTCAGCCTGGGGCTGTTGCCGCCGAGTTGCGTG
CCGCGCGCGATCGCGGGCGTTCGCTGATGAACTCTGCTCGCGTACCATGCCAGGCGGCTC
CGGCGAGGTGGAGGCTCATCAAGGCGCACGGCACGGCGGTGGGCCTGCCGACGGACGACa
ACATGGGGAACAGCGAGGTCGGCCACAACGCGCTCGGGGCGGGCCAGATATACGCCCAGG
GGTAAACGCTCTCTGCTAGCTCTCCGGTTTCGTTCAACTTTCGATCAGCTGACGTCGGTC
GACCACGACGGCGCAGGGCGAAGCTGGTGGATCTGGCGCTCGCCTCCGGGAAGATATAtG
ACGGGGAGGGCTTCAAGTACATCCGGCAGTCTTTCGACGACGGCACCCTGCACCtCATCG
GCCTGCTCAGCGACGGCGGCGTGCACTCGAGGTTCGACCAGCTGcCGGTTTCGTTGCtGC
TACCGCTTGCTCCTGCTGGCAGGTTCCAACTTCCAAACGTCCCCAAACTGGTCGCGACAC
ACAGCTGACGGTTACCGTCTGCCGCCTTCTTTCTTGGCAGCTGCTCCTGAAAGGGGCTAG

CGAGCACGGGGCGAAGAGGATACGCGTCCACGTCCTCACGGATGGCCGCGACGTGCTGGA
CGGCAGCAGCGTCAGGTTCGTGGAGATGCTAGAGGAGGACCTCGCGAGGCTGCGGGAGAA
GGGCGTTGACGCGAGGGTCGCGTCTGGCGGAGGCAGGATGTACGtCACAATGGATCGCTA
TGAGGTAGTACTGCTACCACTATTTGTCAATGTGTTTCGATTTGGATCGTTGGGTTGTCA
GCTAGTAGGAGTAAAACGGTGGCTTGTTGCCACTCTGAGCATAGAGAAGAACGTTCGATC
AGTTGCTGAACCGTGTATCCAATATTGTGAGAATTTTAGTTACCATGATAATTGTGATAG
GACTGAGATTTCACCTAgAAGAAACAATCTGCAGaATACGAGAACCGCTGCTGCATTGTA
TTGGCCAGAAATCAGTTCTGAAAAATGAGTGCAGCTCAGCTACTCTGCAtTTGTTGATTC
GTACACCGCTGaAGTAACCTGTCtTCtTTCGCAGaATGACTGGCAGGTCGTGAAGCGGGG
TTGGGATGCGCATGTCCTTGGCGAAGcCCCAcacaagttcaagagcgctcttgaggctgT
GaAAAAACTCAGGGAGGATCCAAAGGCCAACGACCAGTATCTAcCCCacTTCGTTATAGT
TGATGAAAGCGGAAAACCGGTCGGCCCGATACAGGACGGGGATGCTGTCGTGACGTTCAA
TTTCAGAGCTGACCGGATGGTGATGCTTGCAAAGGCATTGGaATACGAGAACTTTGACaA
AtTTGATCgGAGTTAGGTTcCCCAAGATACGCTATGCTGGTATGCTTCAGTACGACGGCG
AGCTAAAACTTCCGAGCCAtTACCTTGTTGCtCCCCCAgagatagagaggac
>PGM_sg0contig338921_1-488r
GTTGTCGCACCGTCACGTAGGAGCTGTTCCACAAGGCGGGCTCTTCACAAGAAAATCAAT
GGAGCTTTACACCTGTGCACCTCTGGAACTGCCACTTCGGTTCGCGCCGAGACCCTTCCA
GAGCAAAGaAAGCCGCGGCCGGGCCACGGCCACCACCTCCAAAACCCGaCGAGCGATCGG
CCAGCAGCACCGAGCAGACGCCAGAGCCCGGACGGAACCtTTCAGCTCACCCATCAGCCG
TGTCACCAGCTGATCTCGCTTCTGGAAGGCCGACCGTACCGCATCGCATGGCGACGAGcC
CGcCGGCgGAGGGGAAGcCGTGGGAGCTGGCGGCGCACCCGcGGCTGCCCAAgGGGaCgG
TGGTGGCCGTGGtgGTGCTGGACGGCTgGGGCgaGGCgcCGcCCgAccCCTTcAACTgcA
TCCAcGCCGcCgAcACgcCCAcGCTCGACGCtCTCAAGaaggtacgcggcccactcagcc
tggggctg
>PGM_sg0contig341962_1-438r
GTTGTCGCACCGTCACGTAGGAGTTGTTCCACAAGGCGGGCTCTTCACaAAAAAATCAAT
GGAGCTTTACACCTGTGCACCTCTGGAACTGCCACTTCGGTTCGCGCCGAGACCCTTGAG
CAAAGAAAGCCGCGGCCGGGCCACGGCCACCACCTCCAAAACCCGaCGAGCGATCGGCCA
GCAGCACCGAGCAGACGCCAGAGCCCGGACGGAACCtTTCAGCTCACCCATCAGCCGTGT
CACCAGCTGATCTCGCtTCTGGaAgGCCgacCGTAcCGCATCGCATgGCGaCGAGCCCGC
gACgGaggggaanccgtgggagctggcggcgcacccgcggctgcccaagggggacgtggt
ggccgtggtggtgctggacggctgggggcaggcgccgccgcccgaccccttnactgcatc
cacgccgccgacacgccc
>PhytC1_*sg0contig00846_6259-9792
ACCGTTTGCTGCTGAGTTGGGCAACCGGGAATTCTACCAGGACGATGAACGACGAGCTGT
CGGCTTCCCGGAGGTACTGTACTGTGCCGCTCATCATCTTCACCAGCTTCTGGCATATGT
ACAGGCCAAGGCCCTCCCTAGACACCTCTGGGCTGTGCCGGAACATCTCCTGTATCAGGG
CCTCTGGGACGCCCGGTGCTGGATGGACGATCCTGCATTTCACTCAGAAAGTTCAGCTGT
TATCCATGGGGGTATGGGCTTCAGATTCCCCACAAGGTGACGGCGTCATACCACAGTCAC
CGTGTATTCACGACGGCAATGTTTATAGGAATGGATAGTATGGAAAGGAGTCACTTTTAC
ACAGGTGTCATTTGTATTGTTTCAAACTGCAGACTAGTTTTATTAAGAAAAACTGCAGCG
TAGCCATATGCTGGAGGTGACATGTATCACCTGCATCTTGACTGATGCAGTGATAAGATC
CAAAAGATGTGGAAAGTAAGATGCTCAGATATCGTTGCTTGTCAACATAGTCATTTTAGT

TCGTGGGTTACAAATAGCACAACTAGGACTTTAGGAGCAGTGCCAAATTTATTAACAAGA
GCAACCAGCTTAATGAGACAAATGTGTAGTTGTGCTATAGTAATGAAAACTGATGCAAAA
TCCAGAGGTTCGCTTATTCGTGAGGTCACCTTTCAGTTCAAGAACAGGAATCAAAAGCCA
CCTTTCAGTTCAAGAACAAGAATCATAAGATGATTGGTGTGTTACCTGAACTCTAAATGA
GCAATCTGCATGCCAGACCCAATATTTTCCTTCTTGGGAATGACTTGGAGCACAATAGGC
CCTTCGGCTGGTTGGGTAAATTGAAGAGTGCATGTCAGATAGTCTGCTAGGACCTGCTGA
AGCCTTAAATTGTCTCCATAGAGGTACATGCATGATACTTCCACAGGCCAATCACGTTCA
ACAGAAATCCGTTTTTCCTtGCCCAGAGACATGCCTTGCATTAGGACCGTATTCAGAGCT
TCCTCAAGTTTGAATTCTACCATGTTCATGTCCATATAGCTGTAACAGCAAGCATTGGTT
ATCAATAAAAAaTAATTACAAGTGAACAGGGAAGCCTTGTTTTATGCATACATTTTTTAG
CACTTCAAACATTATACATTGATTAAAAATTTCATGTCTCGGCAATTCTAACGACCATTT
TATCTTACTATTATTAATTGGAGGCTCATTTCAAGCCTCTACGTGAAGCCACCTAGAATC
CTATGTGGACACTCTAAAAAAAaTAGAGAAATCCTAGAAATTCTCACAAAAaTCAGAAAC
ATCTGACTATCAATCCAACAACTCTAATCATAATAGTCATTGGATCTGTTATCTTTCCTA
ATAAATTACCCACGTCTATCATTATGAAAATAGATTAAAGGTAACCCCTTAAACATGTAT
CTAAATTACCCACCTCTACCATTAAAAAaTTTAAAGTAACCCCCTAATCTTCGGGCAAAC
TACCCACCTATGCCATTATGAAAACCATAATACTCATTGGATCTATTATCTTTCCTAATA
AATTACCCACCTCTACCATTAAAAAAaTCTAAAGTAACCAACCAATCTTCGTGCACCACC
TATGCCATTATAAAAATAATACAAATAACACCCTAATTTTGCATATAAATTATTCAATTA
TGTAATTATTAAAAATAAAAGTAACCCCTAAATCTAAATATTAAAGTGCACCAATATAAA
ATTCTATTTCTATCATTATTAATATATTATTTATGATGTTATTTACATAAAAATATCACC
ATGTATGCTTGTATGATGGAAGAGATAAGAATACCAATTCACAAACAAATAGTTCAATTA
AATATATATCAAACACACATGGAGCTGTGATGCAAAATGAAATCTATTGCAACTAGATAA
TGATAAATACGTATTTtAGAGTATGTTTTAGAATATTTAATAGATGAATGAGGGTGAGAT
AGGTAATTATAATTATTAGCTATAAGCCTTATTTTATAATAATTTTAATTATCTCGTGCG
GGAGCACGGCCATGGGTTGATAGGCTAGTTCAAATACAAATAGCAAAGTATGTTTCCATG
TGTATGTTGTATGATGCTCCATATATTCATTCTGGCATTATAGGAAATATGGAAACAATT
AAACTAAGAGTACTATGCAGTACTATGGATGATTTGGACAAGGCACTTACCACTGTTCAA
TGCTTTCAAGATCAGTATCGTGTAAAATCTTTTTCAGCTGATCCTGACAGAGAACATTAG
ATGCAACAAGTTGCCTCTGTTCCTCTGTCAACTCAGAATGTTCCAATAAATTATGCGTAA
ATTGCATGCCATTGAGTGGGTTCCTTAATTCTTGGCGAATGTAGGTTAATTCCTTAAAGC
TTTTTGTAGCAGCTTGTTCAGACATTTTCTGAACCTGGAGAGCATGCTGAAGCTCTGGGC
TGGCCACATGCAGAAAGCAAAGAGCACCAGTGATCTTGCCCTCAGCATTTGTCCTCTTCT
TAGCTGTGAGCAGGGATTCCACATACTTGCCATATGTATCGAAGAAACCAAAAAGGAGTT
TCCCAGGATCCTGACCAGAAATCACTGTGTTCATCAGTATGCTAAGTTTTGTTAGAGTAG
CATGATCTTTCACCCTACAGCCATACTCATGAAGGGTGAAAATCTCCCCAATTAACAACT
TGTCTATTGCATCTTCCCTTTTCATACCAGTAATCTTCTGCATGGCTTCATTCCACTCTA
AGCAGGAACCAAGATCATTGATCATAAATATGGGAGGGATAAGCTCACTGGGGTTCTTCA
CTATGGCAACATAGTCTCCTTGTATTCTAGTATACTTATCCATTATCATCTTCTGCCCAG
TCAAATCCTGAGCTACAAAGCAAACTCCAACAACTTTGTCTGAAAGGTCCCGACTGCAGC
AGGAGTTGACCATCAAAATTATAGGGCCATTGCATTCCTGTTGATTGAATGTTTTAAGCT
TGATCTCCAGATTTTGCTCTTCGATTCCTGAAATACAAGAAAGGAGGCACTAAGATGTCA
TACAGAACAATGGACAATTCAGACAATGCTGGAGAGAACAGAAGATCACTTGAAGGCATA
GTGACAAACCTGGTAAAGCTGAGTCCAAAATCTGTTTAACCACTTCAACAGAATCAGACA

TGACAAGATCTACGAGGGGCCTCCCTATGGCTTCCATAACAGGTAATCCAGTGAGTTCTG
CGACTTTATTATTCCACCCATTTATGTTACCGTAAATGTCAACAGCAAAGATAGGGGCAG
TTGCCGTCTCAATTAAGCGGACCATCTCGTTTGTAACTGTTCTTAGCTCAAGTAGCCCCT
GTATCTTCTTTGTATCCTCAGATGGAGCTTTTACAATGGACCTTACATTGTTTTTGTTGG
CATCTTCATCTTGCAGGGAGCCGCGTAATATTAACTGCAAAGAATGGATCGCGTCCATTT
CAACATCCTCCCAGGGAACACTTCTCCATTTAACCACCTCCAAGAAGGCCTTGA
>PhytC1_*sg0contig03093_13282-10617
ACCGCTTGCTGCTGAGTTGGGCGACAGGGAATTCTACCAGGACGATGAACGACGAGCTGT
CGGCTTCCCGGAGGTACTGTACTGTGCCGCTCATCGTCTTCACCAGCTTCTGGCTAATGT
ACAGACCAAGGCCCTCCCTAGACACCTCTGAGCTGTGCCGGAACATCTCCTGTATCAGGG
CCTCTGGGACGCCCGGTGCTGGATGGACGATCCTGCATTCACTCAGAAAGTTCAACAGCT
GCTATCCAGGGGGGGGGGGGGTATGGGCTTCACGTTCCCCACAAGGTGACAGGCGTTATA
CCACCATGATGTTTATAGGAATGGATAGTATGGAAAGGAATCACTTTACACATGTGTCAT
TTGCACTGTTTCAAACTGCAGAGTAGTTTATTTATTAAAAAAACTGCAGCGTAGCCATAT
GCTGGAGGGTGACATGTATCACCTGCATCTTGATTGATGCAGTGATAAGATCCAAAAGAT
GTGGAAAGTAAGACGCTCAGATGTGAATTTGCTAACATTAGGGCTTATCTGTTGCTTGTC
AACATAGTCATTTTAGTTCGCGGGTTACAAATAGCACAATTAGGAGCGGTGCCAAATTTA
TTTACAAGAGCATCCAGCTTGATGAGACAAATGTGTAGTTGTGCTATAGTAGTGAAAACT
GATGCAAAATCCAGAGGTTTGCTTATTTGTGAGGTCACCTTTCAGTTCCAGAACAAGAAT
CAAAAGCCACATTTCAGTTCAAGAACAAGAATTATAAGATGATTGGTGTGTTACCTGAAC
TCTAAATGTGCAATCTGCATGCCAGACCCAATATTTTCCTTCTTGGGAATGACTTGGAGC
ACAATAGGTCCTTTGGCTGGTTGGGTAAATTGAAGAGTGCATGCCAGATAGTCTGCTAGG
ACCTGCTGAAGCCTTAAATTGTCTCCATAGAGGTACATGCTTGATACTTCCACAGGCCAA
TCACGTTCAATAGAAATCCATTTTTCCTTGCCCAGAGACATGCCTTGCATTAGGACCGTA
TTCAGAGCCTCCTCAAGTTTGAATTCTTCCATGTTCATCTCCATATAGCTGTAACAGCAA
GCATTTGTTATCAATAAAAAATGATTACAAGTGAACAGGGAAGCCTTGTTTTATGCATAC
ATCTTTTAGCATTTCAAACATTATACATTGATTAAAAATTTCATGTCTCAGCAATTCTGA
CCACCATTTAAATACAAATAGCAAAGTATGTTTTTCATGTGTATGCTGTATGATGCTCCA
TATATACATTCTAGCATTATAGAAAATATGGAAACAATTAAACTAAGAGTACTATGGAGG
ATTTGGACAATTTGGTACTTACCACTGTTCAATGCTTTCAAGATCAGTGTCGTGTAAAAT
CTTTTTCAGCTGATCCTGACAGAGAACATTAGATGCAACAAGCCGCCTCTGTTCCTCTGT
CAACTCAGAAGGTTCCAATAAACTATGTGTAAATTGCATGCCATTGAGTGGGTTTCTTAA
TTCTTGGCGAATGTAGGTTAATTCCTTAAAGCTGTTTGCAGCAGCTTGTTCAGACATTTT
CTGCACCTTGAGAGCATGCTGAAGCTCTGGGCTGGCCACATGCAGAAAGCAAAGAGCACC
GGTGATCTTGCCCTCAACATTTGTCCTCTTATTTGCTGTGAGCAGGGATTCCACATACTT
GCCATATGTATCGAAGAAACCAAAAAGGAGTTTCCCAGGATCCTGACCAGAAATCACTGT
GTTCATCAGTATGCTAAGTTTTGTTAGAGTAGCATGATCTTTCACCCTACAGCCATAATC
ATGAAGGGTGAAAACCTCCCCAATTAACAACTTATCTATTGCATCTTCCCTTTTCATACC
GGTAATCCTCTGCATAGCTTCATTCCACTCTAAGCAGGAACCAAGATCATTGATCATAAA
TATGGGAGGGATAAGCTCACTGGGGTTCTTCACTATGGTAACATAGTCTCCTTGTATCCT
AGTATACTTATCCATTATCATCTTCTGCCCAGTCAAATCTTGAGCTACAAAGCAAACTCC
AACAACTTTGTCTGAAAGGTCCCGATTGCAGCAGGAGTTGACCATCAAAATTACGGGGCC
ATTGCATTCCTGTTGATTGAATGTTTTAAGCTTGATCTCCAGATTTTGCTCTTCGATTCC
TGAAATACAAGAAAGGAGGCACTAAGATGTCATACAGAACAATGGACAATTCAGACAATG

CTGGAGAGAACAGAAGATCATTTGAAGGCATAGTGACAAACCTGGTAAAGCTGAGTCCAA
AATCAGTTTAACCACTTCAACAGAATCAGACATGACAAGATCTACAAGGGGCCTCCCTAT
GGCTTCCATAACAGGTAATCCAGTGAGTTCTGCGACTTTATTATTCCACCCATTTATGTT
ACCGTCAATGTCAACAGCAAAGATAGGGGCAGTTGCTGTCTCAATTAAGCGGACCATCTC
GTTTGTAACTGTTCTTAGCTCAAGTAGCCCCTGTATATTCTTTGTATCCTCAGATGGAGC
TTCTACAATGGACATTACATTGTTTTTGTTGGCATCTTTATCTTGCAGGGAGCCGCGTAA
TATTAACTGCAAAGAATGGATAGCGTCCATTTCAACATCCTCCCAGGGAACACTTCTCCA
TTTAACCACCTCCAAGAATGCCTTGA
>PhytC1_sg0contig00216_7872-8174r
AAAACCTCACCAAGGAGCATCTTATCAATCACCTCATCTCTGTGCCACCCGGTAAGCTTG
GTCATGGCTGCATTCCACTCAGAGCACCACCCAAATTGGTCAGCACCAAATATGGGAGGA
ATGAGTGGGTTTGGGTTTTGAACGATTGCCTTGTAGTCCCCTTCCACACGAGTAAATTTG
TCCATGACCAACTTATGAACAGTCATATCCTGGGCTACAAAGCACACCCCAACAACATGG
TCATGAAGGTCACGACTGGCACAGGCATTTACAACCAAGATAACAGGGCCATCATCCCTC
TTG
>PhytC1_sg0contig06603_5819-6121
AAAACTTCACCAAGGAGCATCTTATCAATCACCTCATCTCTGTGCCACCCGGTAAGCTTG
GTCATGGCTGCATTCCACTCAGAGCACCACCCAAATTGGTCAGCACCAAATATGGGAGGG
ATGAGTGGGTTCGGGTTATGAACGATTGCCTTGTAGTCCCCTTCAACACGAGTAAATTTG
TCCATGACCAACTTATGAACAGTCATATCCTGGGCTACAAAGCACACCCCAACAACATGG
TCATGAAGGTCACGACTGGCACAGGCATTTACAACCAAGATAACAGGGCCATCATCCCTC
TTG
>PhytC2_*sg0contig00846_9819-10376
CCATTCTCATCTCCATCGGTCGTTTTATGCTTACGTCCACCCCACTTGATCTCCTTTGCT
GTGTGGGCTCGGAACCAGAAGATAAAATCTTTGGAAGAGATCTTTATGGCTGCCATGCCA
CACACAACTTCACGAAGTGCAGCAGCACCAGGATAACCCGCTTCCACTAAGCTGTCGGTA
CTCAGCCCAGTTGAACCATCATGGTTCTCCTGAAGCCACGTGACAATGCTCCTTATCTCT
GCCTCCGAGGGTGTTGATCCGAGCACCCAAAGCTGGTTTTGGTAGTACAGTGCAGTTCCA
TCACACTTTACTAGATCCATTACATTAGGCGACTGGGTAAATATCGCAACAGGAGCTTCT
CGCAGAAGCATATCACACAGAAGGGTTTGCGTTCGGAGGATGTGCCTCTCCTTTGCCTGA
GCAGCCAATTCTACCTCCTTGTTGAGCTGTATGCCAAACAGTTGCAAGAGAAACTCGCAA
GCGTACCTTAGTGGGTAAGGAACGAACCTCGGACTCGTGTGATGGCAAACCACCAGCCCC
CACAGCTTCCTTCCTTTG
>PhytC2_*sg0contig03093_10590-10033
CCGTTCTCATCTGCATCGACCGTTTCATGCTTACTTCCACCCCACTTGATCTCCTTTGCT
GTGTGGGCTCGGAACCAGAAGATGAAATCTTTGGAAGAGATCTTTATGGCTGCCATGCCA
CACACAACCTCACGAAGTGCAGCAGCACCAGGATAACCCGCTTCCACTAAGCTGTCGGTA
CTCAGCCCAGTTGAACCATCATGGTTCTCCTGAAGCCACGTGACAATGCTCTTTATCTCT
GCCTTGGAGGGTGTTGATCCGAGCACCCAAAGCTGGTTTTGGTAGTACAGTGCCGCTCCA
TCACACTTTACTAGATCCATTACATTAGGCAACTGGGTAAATATCCCAACAGGAGCATCC
CGCACAAGCATATCACACAGAAGTGTTTGCGTTCGGAGGATGTGCCTCTCCTTTGCCTGA
GCAGCCAATTCTACCTCCTTGTTGAGCTGTATGCCAAACACTTGCAAGAGAAACTCGCAA
GCGTACCTTAGTGGGAAAGGAACGAACCTCGGAGTCATGTGATGGCAGACCACCAGCCCC
CACAGCTTCCTGCCTTTC

>Rht1_*sg0contig15005_4739-6608
CTCACTGCAATTCAGCGTTCGTTGACGTCTAGCTTCAGCTGTGCCGGCGAGCTGGGTGGA
GGGGGTGCGCCATGCCCGGGGACGTGCATCCTGATCAGCGTCCAAAACCGCACCCGCCCG
ATCACGGCGCGGCGACGCGCCATGCCGAGGTGGCGATGAGCGGGCGCGTGTGCCACCCCA
GGGTCAGGCACCCGTCCTTCTCCTCCACCCTGTACCCGTCGCCGCCGGCGAACAGCGCCA
GTAGCGTGCTCGCCTGCTTGTAGGCATTGGAGCCCAGGTGGACGGGCTCGAACCCGGCGC
CGCCCAGGCGGTTGCGCCACTGCCCCAGCGTCTCGTGCCGCTCGGTGCGCTCCGCACCCT
CGCACGCCACGACGTTACAGATCTGCCGGCCGAGGTACACCTCGGACATGACCTGGTCCG
TgccgccggccgcggccggggcggcATCGGCGGATTGGCCGGAGCCGGCGCCCTCGAGGG
AATCGAACATGGTGGAGTAGTAGTGCAGCGACTCCGTGAAGCGGTCCAGGAATGAGACGG
AGTTGTGGTTGGCCTCCTGCTCGACCACGGTCACGATCCTCGGCCGCACTGCGCGCACCG
TGCCCAGGACCTTCTCCAGGGCGCCGGGCTGCGCCAGCAGCCGATGCAGCTCGAACACCG
AGTTGACGGCGATCACCTCGGGCTCGTCATCCGTATCCTCGCCGTCCGGTTGCAGCATGA
ACGGCTCAAGGTCAGCGAGCGTGGCGGCGACGAGGCCGCGGTACTGGAAGTCGACGCGGA
TTGTGTGCGCGAACTGGGCAAGCTTCCAACCAACCTGCTGCAAAGCGTCGGTCTCGTCCG
GCTGCGGCGGGCCGACGCCGGTGAGGCGGAACGATGGGGGGCCGCCAGGACGGAGGGCAA
GAGCCTGGAGAAGAGCCGGCCACTGCATCCCCTGCTTGATGCCGAAATCGACGACATGGA
CGCGGCGGCAGCCGGCGAACGCCTCCAGGATGGCCTGGTTCGCGGTGAAGTGGGCGAACT
TGAGGTAGGGGCAGGACTCGTAGAAGTGCGCGTGGAGGAGGTCGGCGAAGGCAGCGTCGA
GGAGGGAGCTGTCCGGGGCAGGGCGGAAGCGGTACACGCGGCGAGCGAGCGCCTCGCCGA
AGTAGGCGGCGACCTTGCGCATGGCGCCGCCCTGCGACGATGCCAGCATGGGGATTTGCT
TGACCAGCGCCTCCGCGGCGGTGAAGTTCTCCTGCTGCACGGCCTCCGCGCAGGCCAGCA
GCGCGTGCACGAGCCGGATCCCGGCCTCCTGCGTGTCCACCACCACCACCGGCACCGCGG
GCGCGTTGGCCGCCGCGGACGCTTGCGTCGCCGGCGGGGCCGCCTCGACCACGGAGCTCC
TGGTGCGGCCGCCGTCCAGGGAAGACGACGAGGAAGAGGAAGACGACGTGCTGCCGCCGC
CAGTTCGCATCCGCTTGGGCTCCCGCGTCGAGTCCGTTGACGGGTCCGCCGACGCCGCCA
CCGGCGAGGGGATCGGCTTCAGAGCGTACGTGCTGCTGGACGAGTCGACGGCGGGCGGGA
GATCAAAGTACCCTCCACcggcggcggcgccacccgtgacggtggacgaggtggacgcga
gccgcggagccggcggcgctgtggggagcggcggcggcggcgCGTTGAGCTCGGACAGCA
TGCTCTCGACCCAGGACGACAGGTCGGAGGGGTTGTAGTGCACTGTGTCCGTGGCGAGGT
GCGAGACAAACCCGTCATCGACGGCTGTGGCGGCGCCGCCCACGCCGCCCATTCCCATGG
CCATCTCGAGCTGCTCCAGCTTCTGCGCGACGTCCGCCATGTCGGACGAGCGCACCTTGT
ACCCGAGCGC
>Rht1_*sg0contig35590_2125-3984r
CTCACTGCAATTCAGCGTTCGTTGACGTCTAGCTTCAGCTGTGCCGGCGAGCTGGGTgGA
GGGGGTGCGCCATGCCCGGGGACGTGCATCCTGATCAGCGTCCAAGACCGCACCCGCCCG
ATCACGGCGCGGCGAGGCGCCATGCCGAGGTAGCGATGAGCGGGCGCGTGTGCCACCCCA
GGGTCAGGCACCCGTCCTTCTCCTCCACCCTGTATCCGTCGCCGCCGGCGAACAGCGCCA
GCAGCGTGCTCGCCTGCTTGTAGGCATTGGAGCCCAGGTGGACGGGCTCGAACCCTGCGC
GGCCCAGGCGGTTGCGCCACTGCCCCAGCGTCTCGTGGCGCTCCGTGCGCTCCGCGCCCT
CGCACGCCACGACGTTGCAGATCTGCCGGCCAAGGTACACCTCGGACATGACCTGGTCCG
TGCCGCCGGCCGCGGCCGGAGCGGCATCGGCGGATTGGCCGGCGCCGGCGCCCTCGAGGG
AATCGAACATGGTGGAGTAGTAGTGCAGCGACTCCGTGAAGCGGTCCAGGAATGAGCCGG
AGTTGTGATTGGCCTCCTGCTCGACCACGGTCACGATCCTCgGCCGCACCGCGCGCACCG

TGCCCAGGACCTTCTCCAGGGCGCCGGGCTGCGCCAGCAGCCGATGCAGCTCGAACACCG
AGTTCACGGCGATCACCTCAGGCTCGTCATCCGTGTCCTCGCCGTCCGGTTGCAGCATGA
ACGGCTCAAGGTCAGCAAGCGTGGCGGCGACGAGGCCGCGGTACTGGAAGTCGACGCGGA
TTGTGTGCGCGAACTGGGCAAGCTTCCAACCAACCTGCTGCAAGGCGTCGGTCTCGTCCG
GCTGCGGCGGGCCGACGCCGGTGAGGCGGAACGACGGTGGGCCGCCAGGACGGAGGGCGA
GGGCCTGGAGAAGAGCCGGCCACTGCATCCCCTGCTTGATGCCGAAATCGACGACATGGA
CGCGGCGGCAGCCGGCGAACGCCTCGAGGATGGCCTGGTTCGCGGTGAAGTGGGCGAACT
TGAGGTAGGGGCAGGACTCGTAGAAGTGCGCGTGGAGGAGGTCGGCGAAGGCGGCGTCGA
GGAGGGAGCTGTCCGGGGCAGGGCGGAAGCGGTACACGCGGCGAGCGAGCGCCTCGCCGA
AGTAGGCGGCGACCTTGCGCATGGCGCCGCCCTGCGACGATGCCAGCATGGGGATTTGCT
TGACCAGCGCCTCCGCCGCGGTGAAGTTCTCCTGCTGCACGGCCTCCGCGCAGGCCAGCA
GCGCGTGCACGAGCCGGATCCCGGCCTCCTGCGTGTCCACCACCACCACCGGCACCGCGG
GCGCGTTGGCCGCCGCGGACGCTTGCGTCGCCGGCGGGGCCGCCTCGACCGCGGAGCTCC
TGGTGCGGCCGCCGTCCAGGGATGACGACGAcgAAGAGGAAGACGACGTGCTGCCGCCGC
CAGTCCGCATCCGCTTGGGCTCCCGCGTCGAGTCCGTTGACGGGTCCGCCGACGCCGCCA
CCGGCGAGGGGATCGGCTTGAGCGCGTACGTGCTGCTGGACGAGTCGACGGCGGGCGGGA
GATCAAAGTACCCTCCACCGGCGGTGGCGCCACCCGTGACGGTGGACGAGGTGGACGCGA
GCCGCGGAGCCGGCGGGAGCggGGCgGGGGCGCGTTGAGCTCGGACAGCATGTTCTCGAC
CCAGGACGACAGGTCGGAGGGGTTGTAGTGCACTGTGTCCGTGaCGAGGTGCGAGACGAA
CCCGTCATCGGCGGCGGCGCCGGCGCCGCCCACGCCGCCCATCCCCATGGCCATCTCGAG
CTGCTCCAGCTTCTGCGCGACGTCCGCCATGTCCGACGAGCGCACCTTGTACCCGAGCGC
>Tb1_*sg0contig76312_641-42329nogap
CAGGTCCTTGTATTCTCCCTCACCTCACCTCACCTCATCCACACGAACAGGCAGTATCCT
TCTCCCCCATCCTCTCTTTATAAGATGGCACAGCCCTCTCAGGTAGGTATGCACATGAGC
CCATGCCGCCTCCTCCAGACCTCCTCTCTCTCATTCTCTGTCTGCACTTGCATAGAGAGA
TGATCTGATCACCTAGCCCTCTCTCTTCTCTCTCTCTCTCTCACCTCTGTCTCAAGCACA
AGCCTAGACCTTATGCTGTTATGCATGGCCGGACACATCTGATCATAGGCCACACTCCTC
CTGCCGCTCTCTCACATTCACACACAGAGATCGATCGACACACACTGCTCTTAGTGCCAG
GACCTAGCGGGGAGGGGAGAGGCATCAGGGGGCCTTGGAGTCCCATCAGTAAAGCACATG
TTTCCTTTCTGTGATTCCTCAAGCCCCATGGACTTACCGCTTTACCAACAGCTGCAGCTC
AGCCCGCCTTCCCCAAAGCCGGACCAATCCAGCAGCTTCTTCTACTACCCATGCTCCCCT
CCCTTCGCCGCCGCCGCCGACGCCAGCTTCCACCTCAGCTACCAGCTCGGTAGCGCCGCC
GCCGCAGCGGCCACCCCGCCACAAGCCGTGATCGACTCGCCAGAGCTGCCGCTGCAGCCG
CCGCTGATGGAGCAGGCGCCGGCGCCGGCAACAGAGCAGGACGCCGCCTGCGCCGATGCC
CAAGGCGCCGGCATCAGCCTCGACAGGGCCTCCCCGGCCGCGAGGAAAGACCGGCACAGC
AAGATATGCACCGCCGGCGGGATGAGGGACCGCCGGATGCGGCTCTCCCTCGACGTCGCC
CGCAAGTTCTTCGCGCTCCAGGACATGCTCGGCTTCGACAAGGCCAGCAAGACGGTGCAA
TGGCTCCTCAACACGTCCAAGGCCGCCATCCAGGAGATCATGACCGACGACGCGTCGTCC
GAGTGCGTGGAGGACGGCTCCAGCAGCCTCTCCATCGACGGCAAGCCCAACCCGGCGGAG
CTGGGACTGGGAGCAGGAGATCAGCAGCCGAAGGGTAACGGCCGCAGCGAGGGGAAGAAG
CCGGCCAAGCCAAGAAAGGCGGCGACCGCCCCAAAGCCGCCAAGAAAGTCAGGCAATGCA
CACCCGGTCCCCGACAAGGAGACGAGGGCCAAGGCGAGGGAGAGGGCGAGGGAGCGGACC
AAGGAGAAGCACCGGATGCGGTGGGTGAAGCTTGCATCAGCAATTGACGTGGAGGCGGCG
GCTGCCTCGGTGGCGAGGGACAGGCCGAGCTCGATGTCGAGCAATTTGAACCACCACTCA

TCGTCGAACATGGCGCGTGCTGCTGCTGAATTGGAGGAGAGGTGCTCATCAACCCTCAAC
CTCAACAATGGAGGAAGGATGCAAGAAATCACAGGGGCGAGCGACGTGATCGTAGCCTTC
GGCAATGGAGGATACGGCGCCGGCAACTACTACTGCCAAGAACAATGGGACCTTGGTGGA
GTCGTCTTTCAGCAGAACTCGCGCTTCTACTGAACACGGGCACTAGGTACTAGACTACTC
TTCCAACTTACATATATCCTTTGCCGGCCCCTCCCATGAACTTCCCAATAATTTCTCTTC
CTAGCCTTCGTAATTGACTGGTGACGTTGAACTTGCATTGGTTTGGTCATCGCATGATGT
ATTATATATAGCTAGCATGAACTGTGTTGATTCATCAATGGAATCTAATCTATGTTGATT
CTGTGAATTCCAAAGTACTGAATACCTCTGATGAAGGAGATCAAATAGTATCTGCTGATG
GAACTTCATTATTTGGCAAGCAAGTATAATTCAGCTGTTGCATGAAAGAATGAAGAAAGC
AGCGCTAGCTGAAGTCTGAAGCCTATAGCATGCTGAAAGATAGACCTGCTGGGTAGAAGG
CCCTGAAGGTCAATTTCTCCACCATGATGCATGTTAAAATCTGCAGCTAAGTTACTTAAA
AAATCGGCAGCTAATAATGGCTTTATAGT-CTGATAGTTCATGGATGTTCATAAAATGAT
TGCTTTTACCTCCCACTAATTTCTTTACGAATTGCAAATCTGATTGGATCTTCTTGAGAA
TACTCCACAGTACTTAGGTCCCTCAAGTTAGATTGACATCATATACTCTTCTGCTCGAGT
TTTTCGGAGCCTTATGTATTTTTATATGCGACACACGCTAAAAAGCTATTTGTAAATGCG
AGCTCATTCTTTTTGAAAAGAAAATGCTAGCTCATATTCTAATTACATTAACCATATACC
TCCACATTGAAAATATCATGCATGGTTTCAGTTCGTATACCAAATTACGAATTGCTAGTT
GACTGGATATCATGCAGATAAGTATATATACTATAGCCGACCTCCATGCATACTTAATTA
TTACACATTTTCTGTAGCATATATAGAGTATATGCTGAAAGTTATAGCTATCTATTAAGG
TTCAGTAGGCCACATCAATGTCCTCAGTTCAAATCTGGCCTTTATAGAGAATCCAGTCAT
CTTAAGTAACACCCATTACTAGCACAGTCGACAGAAACCATAGGTCCAGTGCGACAACAT
CATAATCAGGTTAAAGTG
>Tb1_*sg0contig06045_3865-6556nogap
CAGGTCCTTGTATTCTCCCTCACCTCAACTCACCTCATGCACACGAACAGGCAGAGTATC
CCCCCCCCCCCCCCACCCATCCTCTCTATAAGATGGCACAGCCCTCTCAGGTAGGCTCA
CCTCTGTCTCAAGCACAGGCCTAGACCTCTTGCATGGCCGGACACCATAGGCCACACTCC
TCCTCCTGCCGCTCTCTCTCACGCTCACGCTCACAGAGATCGACACACACTGCTCTTAGT
GGCAGGACCTAGCGGGGAGGGGAGAGGCATCAGGGGGCCTTGGAGTCCCATCAGTAAAGC
ACATGTTTCCTTTCTGTGATTCCTCAAGCCCCATGGACTTACCGCTTTACCAACAGCTGC
AGCTCAGCCCGCCTTCCCCAAAGCCGGGCCAATCCAGCACCAGCAGCTTCTTCTACTACC
CATGCTCCCCTCCCTTCGCCGCCGCCGACGCCAGCTTCCACCTCAGCTACCTGCTCGGTA
GCGCCGCCGCCGCAGGGGCCACCCCCGTGATCGACTCGCCGTCGCCGGAGCTGCCGCTGA
TGGAGCAGGCGCCGGCGCCGGCAACAGAGCTGGACGCCTCCGCCTGCGCCAATGCCCAAG
GCGCCGGCGTCAGCCTCGACAGGGCCTCCGCCGCCGCCGCGAGGAAAGACCGGCACAGCA
AGATATGCACCGCCGGCGGGATGAGGGACCGCCGGATGCGCCTCTCCCTCGACGTCGCCC
GCAAGTTCTTCGCGCTCCAGGACATGCTCGGCTTCGACAAGGCCAGCAAGACGGTGCAAT
GGCTCCTCAACACGTCCAAGGCCGCCATCCAGGAGATCATGACAGACGACGCGTCGTCCG
AGTGCGTCGAGGACGGCTCCAGCAGCCTCTCCGTCGACGGCAAGCCCAACCAGGCGGAGC
TGGGACTGCTGGGAGGAGGAGATCAGCAGCCCAAGGGTAACGGGGGGAAGAAGCCGGCCA
AGCCAAGAAAGGCGGCAACCGCCCCAAAGCCGCCAAGAAAGTCCGGCAATGCTCACCCGG
TCCCCGACAAGGAGACGAGGGCCAAGGCGAGGGAGAGGGCGAGGGAGCGGACCAAGGAGA
AGCACCGGATGCGGTGGGTGAAGCTTGCATCAGCAATTGACGTGGAGGCGGCGGCTGCCT
CGGTGGCGAGGGACAGCAGGCCGAGCTCGATGCCGAACAATTTGAACCACCACTCATCGT
CAAACATGGCGGCGTGTGCTGCTGCTGAATTGGAGGAGAGGTGCTCATCAACCCTCAACA

ATGGAGGAAGGATGCAAGAAATCACAGGGGCGAGCGACGTGATCATAGCCTTCGGCGGCA
ATGGAGGATACGGCGGCGCCGGCAACTACTACTGCCAAGAACAGTGGGACCTTGGTGGAG
TCGTCTTTCAGCAGAACTCGCGCTTCTACTGAACACGGGCATGCACTAGGTACTAGACCG
CGTGATGTTGAACTTGGTTTGGTCATCGCATGATGTATTATAGCTAGCATGAACTGTGTT
GATTCATCAATGGAATCTAATCTATGTTGATTCTGTGAATTCCAAACTACTGAATCTCTC
TGATCGATGAGGGAGATCAAATAGTATCTGCTGATGGAACTTCATTATTTGGCAAGCAAG
TATAATTCAGCTGTTCATGAAAGAATGAAAGCAGCTGAAGGCTGAAGCTATAGCATGCTG
AAATTTATACCTGCTGGGTAGCAGTAGAACCCTGCTGGAGGTCAATTTCACTACCATGAT
GTGCATGCAGCTTTTATGCATGTTAAAATCTGCAGCTAAGTTAATTAAAAAAAACTTGCA
GCTAATAATAATGGCTTTATATATATAGTCTGATATATAGTTCATCGGATGTTCATAAAA
TAATTGCTTTTATCTCCCATCAATTTCTTTACGAATTGCAAATCTGATTCCAACTTCTTG
AGAATACTCCACAGTATTTCAGTCCCTCAAGTTAGATTGGCATCATACTCTTCTGCTCGA
GTTTCTCGGAGCCTTATGTATTTTTATATGCGACACACGCTAAAAAGCTATTTGTATATG
CTAGCTCATTTAATTTTTTTTTGAAAAGAAAATGCTTGCTCATATTCTAATTACATTAAC
CCCATACCTCCACATTGAAATATCATGCATAGTTTCAGTTCATATACCAAATTACGAATT
GCTAGTTGACTGGATATCATGCAGCTAAGTATCTATACTAACTAGCCGACTTCCATGCAT
ACTTAATTACACATTTTCTGTAGCATGCATAGAGTATATGCTGAAAATTATGTCTATCTA
TTAAGGTTCAGGAGGCACATCAATGTCCTCAGTTCAAATCTCTGGCCTTTATTGAAAATC
CAGTCATCAGCTTAAGTAACACCCATTACTTGCACAGCCGACACAAACCATAGGTCCAGT
GCGACAACATCATAATCAGGTTAAAGTG
>TE_*sg0cons_contig99597_contig278542_contig361965
ATCAGGAACATACCGAACAAGTACAGGTATCCTCGCCAGTCGCCAATCTCCACATTGCCG
CGACAATGCTATATTTCATGGGCGCCGCTCGCTTAGAATTGCAATTCTGCTGATGCTCAA
GGCCTCTGGGTCGCAGCCAGAAGCTGCTCCTCAACATGCTGGACAACCACTGCATCCACT
CCAACGAGCGGATCGCGGCGAGCGGCGAGGAAGGCGAGGGCCAGCCCTTCTCCTCCTACG
ATTTCGTCTACCTCCCCATCGATTTCAAGTGAGTACTTTTGGCAGATTTTACTTAGGCCC
ATTTTAGTTCCCACCCCCATAACCTCTTAAACGCAAAAAAAAAGTCACCTCGAATGTTTC
CACACATGGAGTACTAAATGAAGTCGTTTCCACACATGGAGTATTAAATGAAGTCTATTT
ATAGAACTTTTTGTATAGATGGGTTGTAAATCATGAGACAAATCTAATGAGACTACTTAA
TTCATGATTTGCAACAATGATGCTCCAGTAATCACTTGCTAATTATTAATTAATTATGGA
TTAATTAGCATCATTAGATTCGTCTCGCAATTTACAATTCATCTGTGTAAAAAGTTTTAT
AAATAGACTTCATTTATTTACTTCAAATTCGCAAGATTCCATTGCAAATTTTTTTTGCAA
AACATCTGAACACGGCCTTATATTTTATGGTGCCCGATGCAACAACACACGCTTGGCAGC
TTGAAAATTCGGTTGTTTTTCCTGGGAATTTGGTGCCCTGAAAATTGCTGATATGGAATG
GGAAATGTGCTCTGATGGGGTGGTGGTAAGGCGAGGAGAGGTTCGGCTGCTGACAGCGTG
AGAGCTTGTGGATCGAGGGATCTTATCTTTGGGTGGGTAGAGCGAGAGTATACACAGTGC
AGTGGAGCCGCAGGCATAGATGGCTCTGGCGTTACTAGCCCAAATGGAAATAAATTTGGC
AGCGGCCCAAGGGGGGGATCACGCCAGTTCCTTCTCGTTTTGATGCCATGGATTCTTTGG
TTGCATGCGGATTCAGCATCTGCTCCTGTTCAACCTGCCTTCCTTTCTAGCCAAAGCCGC
TGCTTCTGCAATCGCATCCATCCGCTGCTTTGCACAGCCCTGCACTTCCATAACAAGGCC
AACACAAAGGGGAGACGCACCGATGGGGGTGTAGACAGTAGAGACAGACGTATCTGTTGG
CTGTTTGTTGCATCTGTGCAAAAAGATAAATAATCGGTGTTTTTGACAATGCGAAAAATT
GAACTTTTGATCAAATAAACAAAAAATTGTAGTGTGCTTAAGCCCTATCTTCACACCAGC
AGCTTTTCTCTTGCACGAAGCAAGTTGAGAAAGGACCTATCAATGCCCAATGACGAGAGG

ACATTAGTGGCTGTCAGAAGGCATGCAGCTTCACATACACATAAAGCAGCATCATGCCGC
AAAGCTTTTTTACTCTGTTCTGTCTCAATTCCAAGGCTTTATAGTAGTAGTTTATACTGT
CGCCATGCCATTCTGAATTTCCATATCTACTAGTTGCACGTAATTCCCTCTGCTCATCAG
CTCCACCAGCTTCCAGCCCAACAGTGTTTTTCTCTCATATTGCCCCAGCATCAGACTCTA
AGCTACCAGCCAGCCAATAGTGTTTTTCTCTCACACCACTCCAGTTCCAGCCCAGCGAAC
CAAGGGAATGAGAATTTTGTCGTGTTTGACACAAGCAAATTAATTTTTGCAACCATAAAA
AAGTTCAGCAACAAGTGCAACGTGGGCTACGGCTTCGTCAACATGACCTCGCCGGAGGCC
GCCGTGCGGCTGTACAAGGCGTTCCACAAGCAGCCGTGGGAGGTGTACAACTCGCGCAAG
ATTTGCCAAGTCACATACGCGCGCGTACAAGTAAGGCAGCTGCTGCCTTTCTCCTAGGCC
AAAAAAATTTTCCTTCGTATCTGCGTGCTCACATTTATTTTTTCAATTGTGTGACTTGAC
ACGCTCACGTTGTGGTTGGCGCATCGCAGGGCCTGGAGGCGCTCAAGGAGCACTTCAAGA
ACTCCAAGTTCCCGTGCGACAGCGACGAGTACCTGCCCGTGGCGTTCT
>TE_*sg0contig04674_63-1916
ATCAGGAACATACCGAACAAGTACAGGTATCCTCGCCAATGCACTCTCCCCATCGCCGCG
ACAATGCTATATTTCATGGGCATCGCACGCTTAGAATTGCAATTGTGCTGATGCTCGAGG
CCTCTGGGTCGCAGCCAGAAGCTGCTCCTCAACATGCTGGACAACCACTGCATCCACTCC
AACGAGCGGATCGCGGCGAGCGGCGAGGAAGGCGAGGGCCAGCCCTTCTCCTCCTACGAT
TTCGTCTACCTCCCCATCGATTTCAAGTGAGTACTTTTGGCCGATTTTATTTATATTTTA
TGGTGCCCGATGCAAGAACAGACGCTTGGCAGCTTGAAAATTCGGTTGTTTTTTGTGGGA
ATTTGGTGCCCTGAAAATTGTCGATATGGTGGAGTACCTGGAATGGGAATGTGCTTGAAG
GCGAGGGGTTCGGCTGCTGACAGCGTGCGTGAGAGCTTGTGGATCTTTGGCCTTTGGGTG
GGTAGCGTGAGAGTACACAGTGCAGTGGAGCAGCAGGCATAGATGGCTATGGCGTTACTA
GCCCAAATGGAAATAAATTTGGCAGCGGCCCGAGGGGGATCACGCCAGGTCCATCTCGTT
TTGATGCCATGGATTCTTTGGTTGCATGCGGATTCAGCATATATATCTGCTGCCTGTTCA
ACCTGCCTGCCTTTCTAGCCAGCAGCTGCTTCTGCCCTCTGGGATCGCTTCCCTGCCTGC
CTTTCTTGCTTTGCACTTCCATAACAAGGCCAACACAAAGGGGAGACgCAGTGTTGGAGA
TGTAGAGAGAGTAGAGACTAGAGAGGCGTCAGTTGCGTCATGGCAATGTGAAAAAAATCG
AACCGTTTGGGAAAAAAACGAACCTTTTTGAAGATGTAAAAAGATGAACTTTCGATCGAA
TATGCTTTCTCCCTATTAAAATGTACTCTGGAGTTTTTAGAATAGAATAGATTATGACTG
GGTAGAGAAGACTCTTCTACCCCTAATTATTAAATATTAGGATTCTATTTGGGTCACTAA
ATATATGCTAATTAAAGCAGTATGCCTTTTTCCATGCATCTTCCTCACATGCACTTCTAT
ATGGTTGAATGCATGTCTTTCTGTTGGAAAAGATTCGAAAACGATGCACAATTAAGATAG
TTGGATCCACTCTCAATCCagaatgtcttatatttgtggacaaattttgaattctaaaat
gctctatatttcaggacggagcgagtaAAGTTTATGCAAACAAATGCTCTTTCTTTACAC
CAGCAGCTTTCCTCTTGCACCTATCAATGCCCAATGACGAGAGGACATTAGTGGCCTGCC
AAAAGGCATGCAGCAGCTTCACAGTTCACATACACATCATGCCACAAGCTTTTTTACTCT
GTTCTGTCTCAATTCTAAGGCTTTAGTAGTTTACTCGTTGCCATGCCATTCTGAATTTCC
ATTTCTACTAGCACGTAATGAGGATCTTTTTTTTACGTGTTTGACCCAAGCAAATTTCTT
TTTGCAATAATAAAAAATTCAGCAACAAGTGCAACGTGGGCTACGGCTTCGTCAACATGA
CCTCGCCGGAGGCCGCCGTGCGGCTGTACAAGGCGTTCCACAAGCAGCCGTGGGAGGTGT
ACAACTCACGCAAGATTTGCCAAGTCACATACGCGCGCGTACAAGTACGGCAGCTGCCTT
TCTCCTAGGCAAAAAATATTTTTGTACCGTATCTGCGTGCTCACATTTTTCAATCGTGTG
GCTTGACACGCTCACATTGTGGTGGCGCTGCGCAGGGCCTGGAGGCGCTCAAGGAGCACT
TCAAGAACTCCAAGTTCCCGTGCGACAGCGACGAGTACCTGCCCGTGGCGTTCT

>Vrn3_*sg0contig07490_4864-7121
AGAACACTGATTCAAAGAAAGTCTTCCCAAATAACAATAATAATTCAGGCCTTGCTGGTA
GCCATGCTCAGAAAAAGCAGCCAAGAAGGGGTGAGCATCTTGTTCAACTATGTGAAAAAG
GAATATCTCAAGACACAAAGCCTCCTAACATAAGGATATGCTTAAATTCAGCCTGCAAAG
CAGTCATGAACTTAGATGATGCATTTTGTAAGAGGTGTTCGTGTTGTATTTGTCATGAGT
TTGATGGCAACAAAGATCCTAGCCTTTGGCTGGTCTGCTCATCAGAGACTGGTGGTAGGG
ATTGTTGTGGGTTATCCTGCCATATTGAGTGTGCACTCCAGCATCAAAAGGTAGGATGCA
CAGAACTTGGACAATCTATACAGCTTGATGGTAACTACTGTTGTGCTGCATGCGGCAAGG
TTACTGGAATTCTTGGGTGAGTGATTTGCATCTACCTTGGACATCAATATTTATTTCAGT
TGAAAAACTCTAAGCATTGCTGGCCACTATCACATCTAGATGTTCGCTATCTGATCTAGA
CTTTAACATCATTGATTTATTTGGTTTTAAATTATTTGTTCTGGATGTTACTTGATATTG
GCGAACCTTTTGCACTCTAAATATTGGTTGAAGGATTCATCACAATGATATTTGTAGGTA
CTTTGAGCATTATGTGCAGATGAGCTCTGCTATGCATTATATTCATGTTTTTCTAAAAAA
AATGTGAACATTATAGTTTGTTGGATCTAAGGTACCTTTAAACTATTTGAGCAGGTTTTG
GAAAAGGCAGCTGATAGTTGCTAAAGATGCCCGTCGAGTTGATAGTTTCTGTTCGCGCAT
ATATCTGAGTCATAGACTTCTGGATGGCACAACCCGTTTTAAAGAGCTGCATCGGATTGT
AGAGGATGCAAAAGCAAAGTTGGAAACTGAAGTTGGGCCCCTTGATGGTACATCATCCAA
GATGACCCGTGGCATTGTTGGACGGCTGTCTGTTTCTGCTGATGTGCAGAAACTTTGCTC
TTTAGCAATTGAAAAGGCAGATGAGTGGCTGAGGTCAAACATTCCATCTGAAACAAAACA
AATTGGTAAACTATTTTAGTCATGTAAAGTTTACCACTTTCCAGTTCTTTTGTGCAAGTG
ATAGCTTTATGAACTTAGTGAACTTTTTTTTTTGCTGCAGATACACTTCCTGCTACCTGC
AGGTTTAAATTTGAAGATATTACAGAATCATCATTGTTGTTGGTTCTTAAGGAAGTTGTT
TCCTCAACGCACCATGTCATCAAAGGATATAAACTCTGGTACTGGAATAGCAGGGAACCT
CCATACACAGGAGAGCCTGCTGTTTTCCCCAAAGACCAAAGAAGGATACTGATATCCAAT
CTGCAGCCCTGCACAGAGTACTCCTTCCGTATTATTTCATTTACTGTGGATGGTGAACTG
GGCCTTTCTGAACACAAGATCTTTACCAAGAGTGTGGAGATCATTCGTAAGAACAAAGAG
AACAGGGCAGAAGGTTGGCCATCTTCTGCCAAGAGAACTGGTAAGAGTGATCATGGTACG
TTGTCAGGCTTCCAGGTCCGCCAGCTGGGTAATGTCTTGCGGAAGGCTGATGAGAATGGT
TACCCTAGTGCATTGTGTAAAGATGAGATTGAAGATTCATGCGACCAGAGTGATTCTGTA
ATACTAGAAAAGGACCAGGTTCCATGCAGTGCATCCCACAGGCTCGATCTTAATGAGACC
TCAGTCCCGGACCTGAATGCAGAGGTAGTCATGCCAACAGAGTGCTGCCAGGATGAGAAC
GGATGCAGCTCAGGAAAGAACACACTGGCACAGTCCAATGGTTGTGGTGACTCCCAGACT
TTTGCCGAGGGGTATTCTGGGGGGGCGATCATGGAATCCCAGTCACAGAGTCGCAAGCAG
ACATCTGACCTGGAGCAGGAAACCTGTGCTGATGGCAGCAACTTGGCAACCGCCTCAGCT
AGGCTCTTCTCCCATAGGTTAGGCCAGTTAGATGACAACTATGAATACTGTGTGAAGGTT
ATACGATGGCTAGAATGTTCTGGACACATAGAAAAGGATTTCAGGATGAAATTCCTAACC
TGGTTTAGCTTGAGATCAACACAGCAGGAGCGTAGAGTTGTGATCACCTTCATCCGCACC
CTCCTTGATGATCCCAGCAGTTTGGCCGGCCAGCTCCTGGATTCATTCCAGGAAATCGTT
GCTAGTAAGAAGCCAAGAACTGGTTTCTGTACTAAGCT
>Vrn3_*sg0contig16433_4192-6449
AGAACATTGATTCAAAGAAAGTCTTCTCAAATATCAATAATAATTCAGGCCTTGCTGGTA
GTCATGCTCAAAAAGAGCAGCCAAGAAAGGGTGAGCATCTTGTTCAACTATGTGAAAATG
GAATATCTCAAGACACAAAACCTCCTAACATAAGGATATGCATAAATTCAGCCTGCAAAG
CAGTCATGAACTTAGATGATGCATTTTGTAAGAGGTGTTCGTGTTGTATTTGTCATGAGT

TTGATGGCAACAAAGATCCTAGCCTTTGGCTGGTCTGCTCATCAGAGACTGGTGGTAGGG
ATTGTTGTGGGTTATCCTGCCATATTGAGTGTGCATTCCAGCATCAAAGAGTAGGATGCA
CAAAACTTGGACAATCTATACAGCTTGATGGTAACTACTGTTGTGCTGCATGCGGCAAGG
TTATTGGAATTCTTGAGTGAGTGATTTGCATCTACCTTGGACAGTAATATTTATTTCAGT
TGAAAAAACTCTAAGCATTGCTGGCCACTGCAGCTTCTAGATGTTCGCTATCTGATCCAG
ACCTGTTATCATTGATTTATTGGGTTTTAAATTATTTGTTCTGGATGTTCGTTGATATTA
GTGAACCTTTTGCACTTTAAATATTGGTTGAAGGATTCATCACAATGATATTTGTAGGTA
CTTCGAGCATTATGTGCAGATGAGCTCTGCTATGCATTGTATTCATTTTTTAAAAAAAAA
AATTGAACATTATATTTTGTTGGATCTAACGGCACCTTTAAACTATTTGAGCAGGTTCTG
GAAAAGGCAGCTGGTAATTGCTAAAGATGCCCGTCGAGTTGGTAGTTTCTGTTCACGCAT
ATATCTGAGTCATAGACTTCTGGATGGCACAACTCGTTTTAAAGAGCTGCATCGGATAGT
AGAGGATGCAAAAGCAAAGCTGGAAACTGAAGTTGGGCCCCTTGATGGCACATCATCCAA
GATGGTCCGTGGCATTGTTGGACGGCTGTCTGCAGCTGCTGATGTGCAGAAACTTTGTTC
TTTAGCAATTGAAAAGGCAGATGAGTGGCTGAGGTCAATCATTCCATCTGAAACAAAAAA
ATTGGTAAATTATTTCAGTCATGTAATGTTTACTACTTTCCAGTTCTTTTGTGCAAGTGA
TAACTTTATGAACTTAGTGAACTTTTTTTTGCTGCAGATACACTTCCTGCTGCCTGCAGG
TTTAAATTTGAAGATATTACACATTCATCATTGGTGTTGGTTCTTAAGGAATCTGTTTCC
TCGCCGCACCATGTCATCAAAGGCTATAAACTCTGGTACTGGAATAGCAGAGAACCTCCA
TACATAGGGGAGCCTGCTGTTTTCCCCAAAGACCAAAGAAGGATACTAATATCCAATCTG
CAGCCCTGCACAGAGTACTCCTTCCGTATTATTTCATTTACTGTGGATGATGAACTGGGC
CATTCTGAACACAAGATCTTTACCAAGAGTGTGCAGATTATTTGTAAGAAAAAAGAGAAC
AGAGCAGAAGGTTGGTCATCTTCTGCCAAGAGAGCTGGTAAGAGTGAAAATGGTACGTCA
TCAGGCTTCCAGGTCCGCCGGCTGGGTAATGTCTTGCGGAAGGCTGACGAGAATGGTTAC
CCCAGTGCATTGTGCAAAGATGAGATTGAAGATTCATGCGACCAGATTGATTCTGTAATA
CTAGAAAAGAACCAGGTTCCATGCAGTGCATCCCACAAGCTTGATCTTAATGAGACCTCA
GTCCCGGACCTGAATGCAGAGGCAGTCATGCCATCAGAGTGCTGCCAGGACGAGAACGGA
TGCAGCTCAGGAAAGAACACACTGACAAAGTCCAATGGTTGTGGCGACTCCCAGACTTTT
GCCAAGGGGCATGTAGGGGAGGCGTCTGTCATGGAATCCCAGTCACAGAGTCACAAGCAG
ACATCTGACTTGGAGCAGGAAACCTGTACTGATGGCAGCAACTTGGCAGCCGCCTCAGCT
AGGCTCTTCTCCCGTAGGTTAGGCCAGTTAGATGACAACTATGAATACTGTGTGAAGGTT
ATACGATGGCTGGAATGTTCTGGACACATAGAAAAGGATTTCAGGATGAAATTCCTAACC
TGGTTCAGCTTGAGATCAACACAGCTGGAGCGTAGAGTTGTGATCACCTTCATCCGCACC
CTCCTTGATGATCCCAGCAGTTTGGCCGGCCAGCTCCTGGATTCATTCCAGGAAATTGTT
GCTAGTAAGAAGCCAAGAACTGGTTTCTGTACTAAGCT
>Vrn3_sg0contig00021_43010-45413r
GGATCTGCAAAAATTTAGCCTGTAAAGCTGCTAGACCATCAGAAGATTCATTCTGCAAGA
GGTGCTCATGTTGTATTTGTCACAAGTTTGATGACAACAAGGATCCTAGTCTATGGTTAG
TTTGTTCATCTGAAAATGATAGCAAGAATTGCTGTGGCTCTTCTTGTCATATTGAATGTG
CTTTCCGACACAAGAGGGTAGGATGCTTTGATCTAGAGCAAATTATACATCTCGATGGGA
GTTATTCTTGTGCTTCATGTGGAAAGATTTCTGGAATACTAGGGTAAGCACAGTTTCTAG
TTAATTCTCTAAGTCTCAATATTCCTATTGTTATTTCGCTTCttTTTTTTTCATTTGTAT
GACACATGCTAATATTTAATTCTTTTGCATGAGAAGTGGCTTGCTCTTCTGCAGTGATGA
AATTATTGTGCATAAAGTCAAACCATTTTAGAGCTGTGGTGTAAtTTTTTTTTTACCTTT
GACAGAAATTTCACTCCCATTGGCAGTAGATAAATAATTGTGTGGAACCATAGACATGCA

ACATTCAAGTCAACTGTTACTAGGATCCGATTCATCTTTGCAGTGGACATACTGGTCCTG
ATGGGGTATTCCTCCAGTGTATATAACATTGTATTTGTAGATGCAACGGACTCTTTTTA
TTAAATGCTGTAAATGTACATCTTGAATGAATAAACCTGGAAGGATGAAATTATCTTTTC
AAATTTAATTTCTTTGGAGAATGGAATTGTTCGTTTTCCCTAACAGGTATTGTGTATCAA
ATGCCAGCTCTTCTAAATTTTGACATAGTAGTGGTTCTCTTAATCGAAATCAAGCATCCA
ATGTTAAAGTTGTGGTATTGATTTGCATATAGATACAGTATTTACTTGGGGCATTATTGG
AACACCTGCTGTGGCTGTTGGTCATTCATGGTAGACTTGATTAATTAACATTAACATATG
TATTAATCTTTGTAGCAGATATTGGAAAAGGCAATTAGTAATTGGAAAAGATGCTCGCCG
AGTTGATAATCTCTGCGAACGTATTTATTTGAGCTATAGGCTATTGGAGGGAACTAGCCA
TTTTAAAGAACTGCATGCCATTGTCGAAGATGCCAAAGCAAAATTGGAAAGTGAGGTTGG
CCCACTTGATGGAATGTCAGCAAAGAATGCACGTGGTATCGTAAGCAGGTTCTCAGCTGG
TATTGCCGCGCAGAAACTATGCTCTACAGCAATTCAAAGAGCTGATGAGCTGTTGAGTTC
TCCTGACCTGCATCTTCGAGGTAATGATCATCCTTGCATAAAATATTTAGCATCATGGTT
TTGGTGTTGCTGCAGAAGGCTTACACACTTCTTTGTTTGCAGATTCATTACCTGCTGCCT
GTAGATTCAAATTTGTAGACATAACGTCTTCTTCAGTTGTTGTCATTTTGAAAGAAACCT
CATCATCTGACATAATCAAAGGTTATAAACTATGGTACTGGAATAGCAGAGAGAGACCAA
GTGTGGAAAAGCCTGTGGTTCTGCCCAAAGATGAAAGaAAAATAtTAGTTTTTAACCTAT
CCCCATGCACAGAGTATTGTTTCAGAGCTATATCATTCACTGAGGATGGGGTACTTGGCC
ATTCAGAATCTAGGTGTCGTACCAATAGCAAGGAGATATTTTTCAAGCGTGCTACACAGA
ATTCAGGAGGCGCACATACACAAAAAAGAGACAGAAGTCAGTCTTTTAAGTCAACTGGAT
TCAGTATTGGGGGTCTTTGGAAGAGTATGCAGGAAACGTGGGGCGAAGAGGGCTGTTTTG
AAGGGTTTTGTGATGATACACATGAAGGTTCATGGAGCAGAAGTGGCCCAGACACAGAGT
TCTCTGGTGCTTGTCGCAAACTTcATTTCAACGCGCCTTCTGTTCCTGACCTAAACATTG
AGGTGCCTGTGGCCATGGACTACACTGAGGAGCATTATGATTCAAAGAAGAGACTTGTAA
GATCAAATGACAGTGGTGACTCTGAAACCTGCGCGGTTGGCCGGCGTGCAGAACCACCTG
CTGTTGAATCTCGGCCAGTAGGCAAGGTGAATGGCACACACATTGATAGATGTGAGCAGA
ATGGTGCTTCTGCTATTTGTCGGGAAAAACAGCTTTCTGGAACGACAAGGCAGTTGGATG
GGAGTTATGAGCATTGTGTGAAGGTAATTAGGCAGTTGGAGTGTGATGGACACATTGAGA
ACGGTTTCAGGATGAAGCTCTTGACTTGGTATAGTCTAAGATCGACAGACCAGGAGCGTT
GGGCTGTGAACACATTCATCAAGACACTAAGTGAAGAACCAAGCAGCCTGGCCGAGCAGC
TCATCGATTCTTTTGGAGAAATCATAAACTGCAAGAAGTCGAGAACCGGTTTCTGCAACA
AGCT
>Vrn3_sg0contig38175_2664-2882
TGAAGGTTATACCGTGGTTGGAATGTTCTGGACACATAGAAAAGGGTTTCAGGATGAAAT
TCCTAACCTGGTTCAGCTTGAGATCAACAGCATGGGCGTATAGTTGTAATCCCCTCCATC
CACACCCTCCTTGATGATCCCAGCAGTTTGGTCGGCCAGCTCCTGGATTCATTCCAGGAA
ATTGTTGCTCGTAAGAAGCCAAGAGCTGGTTTCcGTACT
>Vrn3_sg0contig49546_1490-3897
GGATCTGCAAAAATTTAGCCTGTAAAGCTGCTAGACCATCAGAAGATTCATTCTGCAAGA
GGTGCTCATGTTGTATTTGTCACAAGTTTGATGACAACAAGGATCCTAGTCTATGGTTAG
TTTGTTCATCTGAAAATGATAGCAAGAATTGCTGTGGCTCTTCTTGTCATATTGAATGTG
CTTTTCGACACAAGAGGGTAGGATGCTTTGATCTGGAGCAAATTATACATCTCGATGGGA
GTTATTCTTGTGCTTCATGTGGAAAGATTTCTGGAATACTAGGGTAAGCACAGTTTCTAG
TTAATATTCTCTAAGGCTCAATGTTCCTATTGTTGTTTTGcTTCTTTTCTTCATTTGTAT

GACTTGCAAATAGTTTAATTATTTTGCATGAGAAGTGGCTTGCTCGTCTGCAGTGATGAA
ATTACTGTGCATAGAGTCAGAAACCATTTTAGAACTGTGGCGTATTATTCTTTACCTTTG
ACAGAAATTTCATTCCCATTGGTAGTAGATAAATAATTATGTGAAACCATTTTAGACATG
CAACATTCAAGTCAACTGTTACTAGGATCCGATTCATCTTTGCAGTGGACATACTAGTCC
TCATGGGGTATTCCTCCAATGTATATAACATTGTAGTTTATAATTGCAACGgTCTCTTTT
TATTAAATGTTGTAAATGTACATCTTGAATGAATAAACCTAGAAGGATGAAATTATCTTC
TCAAAATTAAATTCTTTGGAGCATGGAATTGTTCGATTACCCCAAACAGGTACTGTGTAT
CAAATGCCAGCTCTTCTAATTTTGACATACTAGTGGTTTCTTAATTGAAATCAAGCATCC
AATGTTAAAGTTGTGGTATTGATTTGCATATAGATACAGTATTTACTTGGGGCATTATTG
GAACACCTGCTGTGGCTTTTGGTCATTCATGGTAGACTTGTTTAATTAACATTAACATGT
GTATTAATCTTTGGTGCAGATATTGGAAAAGGCAATTAGTAATTGGAAAAGATGCTCGCC
GAGTTGATAATCTCTGCCAACGTATTTATTTGAGCTATAGGCTATTGGAGGGAACTAGCC
ATTTTAAAGAACTGCATGCCATTGTCGAAGATGCCAAAGCAAAATTGGAAAGTGAGGTTG
GCCCACTTGATGGAATGTCAGCAAAGAATGCACGTGGTATCGTAAGCAGGTTCTCAGCTG
GTATTGATGTGCAGAAACTATGCTCTACAGCAATTCAAAGAGCTGATGAGTTGTTGAGTT
CTCCTGACCTGCATCTTCGAGGTAATGATCATCCTTACATAAAATATTTACCATGATGGT
TTTGGTGTTGCTGCAGAAGGCTTACACACTTCTTTGTTTGCAGATTCATTACCTGCTGCC
TGTAGATTCAAATTTCTAGACATAACGTCTTCTTCACTTGTTGTCATTTTGAAAGAAACC
TTGTCATCTGACACAATCAAAGGTTATAAGCTATGGTACTGGAATAGCAGAGAACAACCA
AGTGCGGAAAAGCCTGTGGTTCTGCCCAAAGATGAAaGAAAAaTATTAGTTTTTAACCTA
TCCCCATGCACAGAGTATTGTTTCAGAGTTATATCATTCACCGAAGATGGGGTACTTGGT
CATTCAGAATCTAGGTGTCGTACCAATAGCAAGGATATATTTTTCAAGCGTGCTACACAG
AATGCAGGAGGCACACATACACAAAAAAGAGACAGAAGTCATTCTTTAAAGTCAAGTGGA
TTCAAGATTGGAGGTCTTTGGAAGAGTATGCAGGAAACTTGGGGCGAAGAGGGCTGTTTT
GAAGGGGTTTGTGAAtATACACATGAAGGTTCATGGAGCAGAAGTGCCACAGACACAGAG
TTTTCTGGTGCTTGTCGCAAACTTCATTTCAACGCATCTTCTGTTCCTGACCTAAACATT
GAGGTGCCTGTGGCCATGGATTACACCACTGAGAAGCATTATCATTCAAAGAAGGGACTT
GTAAGATCAAATGACAGTGGTGACTCTGAAACCTGCGCAGTTGGCCGGAGTGCAGAACCA
CCTGCTGTTGAGTCTCGGCCAGTAGGCAAGGTGAATAGCACACACATTGATATATGTGAG
CAGAATGGTGCTTCTGCTATTTGTCGTGAAAAACAGTTTTCTGGAACGACAAGGCAGTTG
GATGGGAGTTATGAGCATTGTGTGAAAGTAATTAGGCAGTTGGAGTGTGATGGACACATT
GATAACAGTTTCAGGATGAAGTTCTTGACTTGGTATAGTCTAAGATCGACAGACCAGGAG
CGTAGGGCTGTGACCACATTCATCAAGACACTAAGTGAAGAACCAAGCAACCTGGCCGAG
CAGCTCACCGATTCTTTCGGAGAAATCATAAACTGCAAGAAGTCAAGAACCGGTTTCTGC
AACAAGCT
>Dw3_*sg0contig117938_2825-628
AGAAGCAGCGCATCGCCATCGCCCGCGCCATGCTCAAGAACCCGGCCATCCTGCTGCTGG
ACGAGGCCACCAGCGCGCTGGACTCGGAGTCGGAGAAGCTGGTGCAGGAGGCGCTGGACC
GCTTCATGATCGGGCGCACCACCCTGGTGATCGCCCACCGCCTCTCCACCATCCGCAAGG
CCGACCTGGTGGCCGTGCTGCAGGGCGGCGCCGTCTCCGAGATGGGCACCCACGACGAGC
TCATGGCCAAGGGGGAGCACGGCGGCACCTACGCCAAGCTCATCCGCATGCAGGAGCAGG
CGCACGAGGCCGCCCTCGTCAACGCCCGCCGGAGCAGCGCCAGGCCCTCCAGCGCCCGCA
ACTCCGTCAGCTCACCCATCATAACGCGCAACTCCTCCTACGGCCGCTCCCCCTACTCGC
GCCGCCTCTCCGACTTCTCCACTGCCGACTTCTCCATCCACGACCACCACCACCAGCATC

AGCCGCCGGTGGTGGCCTTCCGCGCCGGCGCCAGCTCCTTCCTCCGCCTCGCCAGGATGA
ACTCGCCGGAGTGGGGCTACGCGCTGCTCGGCTCCCTGGGCTCCATGGTCTGCGGCTCCT
TCAGCGCCATCTTCGCCTACGTCCTCAGCGCCGTGCTCAGCGTCTACTACGCGCCGGACC
CGCGCTACATGGAGCGCCAGATCGCCAAGTACTGCTACCTCCTCATCGGCATGTCGTCGG
CCGCGCTGGTGTTCAACACGGTGCAGCATGTGTTCTGGGACGCCGTCGGCGAGAACCTCA
CCAAGCGCGTGCGCGAGAAGATGTTCGCCGCCGTGCTCCGCAACGAGATCGCCTGGTTCG
ACGCCGACGAGAACACCAGCGCGCGTATCGCCGCCCGGCTCGCGCTCGACGCCCAGAACG
TGCGCTCCGCAATCGGGGACCGCATCTCCGTCATCGTCCAGAACTCGGCGCTGATGCTCG
TCGCCTGCACCGCGGGCTTCGTCCTCCAGTGGCGCCTCGTCCTGGTGCTCCTGGCTGTGT
TCCCGCTCGTGGTCGGCGCCACCGTGCTGCAGAAGATGTTCATGAAGGGCTTCTCGGGGG
ACCTGGAGGCCGCGCACGCCAGGGCCACGCAGATCGCCGGCGAGGCCGTCGCCAACCTGC
GCACCGTGGCGGCCTTCAACGCGGAGCGCAAGATCACGGGTCTCTTCGAGGCCAACCTGC
GCGGCCCGCTGCGGCGGTGCCTGTGGAAGGGGCAGATCGCCGGCAGCGGCTACGGCGTGG
CGCAGTTCCTGCTGTACGCGTCGTACGCGCTGGGGCTCTGGTACGCGGCGTGGCTGGTGA
AGCACGGCGTCTCCGACTTCTCGCGCACCATCCGCGTCTTCATGGTGCTCATGGTGTCCG
CCAATGGCGCCGCCGAGACGCTGACGCTGGCCCCCGACTTCGTCAAGGGCGGCCGCGCCA
TGCGCTCCGTGTTCGAGACCATCGACCGCAAGACGGAGGTGGAGCCGGACGACGTGGACG
CGGCGCCCGTGCCTGAGCGGCCGCGCGGCGAGGTGGAGCTCAAGCACGTGGACTTCGCCT
ACCCGTCGCGGCCGGAGGTGCAGGTGTTCCGGGACCTGAGCCTCCGTGCGCGCGCCGGCA
AGACGCTGGCGCTGGTGGGGCCGAGCGGGTGCGGCAAGAGCTCGGTGCTGGCGCTGGTGC
AGCGCTTGTACGAGCCCAGCTCCGGGCGCGTGCTCCTGGACGGCAAGGACGTGCGCAAGT
ACAACCTGCGGGCGCTGCGGCGCGCCATGGCGGTGGTGCCGCAGGAGCCCTTCCTGTTCG
CGGCGAGCATCCACGACAACATCGCCTACGGGCGCGAGGGCGCGACGGAGGCGGAGGTGC
TGGAGGCGGCGGCGCAGGCCAACGCCCACAAGTTCATCTCGGCGCTGCCGGAGGGGTACC
GGACGCAGGTCGGGGAGCGCGGGGTGCAGCTGTCGGGCGGGCAGCGGCAGCGGATCGCCG
TGGCGCGCGCGCTGGTGAAGCAGGCGCCCATCCTGCTGCTGGACGAGGCCACCAGCGCGC
TGGACGCCGAGTCGGAGCGGTGCGTGCAGGAGGCGCTGGAGCGCGCGGGGGCCGGCCGCA
CCACCATCGTGGTGGCGCACCGCCTCGCCACCGTGCGCAACGCCCTCACCATCGCCGTCA
TCGACGACGGCAAGGTGGTGGAGCAGGGGTCGCACTCG
>Dw3_*sg0contig26301_4059-6265
AGAAGCAGCGCATCGCCATCGCGCGCGCCATGCTCAAGAACCCGGCCATCCTGCTGCTGG
ACGAGGCGACCAGCGCGCTGGACTCGGAGTCGGAGAAGCTGGTGCAGGAGGCGCTGGACC
GCTTCATGATCGGGCGCACCACCCTGGTGATCGCCCACCGGCTGTCCACCATCCGCAAGG
CCGACCTGGTGGCCGTGCTGCAGGGCGGCGCCGTCTCCGAGATGGGCACCCACGACGAGC
TCATGCAGGAACACACCACCTACGCCAAGCTCATCCGCATGCAGGAGCAGGCGCACGAGG
CCGCCCTCGTCAACGCCCGCCGGAGCAGCGCCCGGCCCTCCAGCGCCCGCAACTCCGTCA
GCTCCCCCATCATGACGCGCAACTCCTCCTACGGCCGCTCCCCCTACTCGCGCCGCCTCT
CCGACTTCTCCACCGCCGACCTGTCCATCCACGACCACCACCAGCATCATCAGCCGGCGA
TGGCCTTCCGCGCCGGCGCCAGCTCCTTCCTCCGCCTCGCCAGGATGAACTCGCCCGAGT
GGGGCTATGCGCTGCTCGGCTCCCTGGGCTCCATGGTCTGCGGCTCCTTCAGCGCCATCT
TCGCCTACGTGCTCAGCGCCGTGCTCAGCGTCTACTACGCGCCGGACCCGCGCTACATGG
AGCGCCAGATCGCCCGGTACTGCTACCTCCTCATCGGCATGTCGTCTGCGGCGCTGGTGT
TCAACACAGCGCAGCACGTGTTCTGGGACGCTGTCGGCGAGAACCTCACCAAGCGCGTGC
GCGAGAGGATGTTCGCCGCCGTGCTCCGCAACGAGATGGCCTGGTTCGACGCCGACGAGA

ACGCCAGCGCGCGTCGCCGCCCGCCTCGCGCTCGACGCCCAGAACGTGCGCTCCGCCA
TCGGGGACCGCATCTCCATCATCGTCCAGAACTCGGCGCTGCTGCTCGTCGCCTGCACCG
CCGGCTTCGTCCTCCAGTGGCGCCTCGCGCTGGTGCTCCTGGCCGTCTTCCCGCTCGTCG
TCGCTGCCACAGTACTGCAGAAGATGTTCCTCCAGGGCTTCTCGGGGGACCTCGAGGCCG
CGCACTCCAGGGCCACGCAGATCGCCGGCGAGGCCGTTGCCAACCTGCGCACCGTGGCGG
CCTTCAACGCGGAGCGCAAGATCACGGCGCTGTTCGCGGCCAACCTGCGCGGCCCGCTGC
GGCGGTGCGTGTGGAAGGGGCAGATCGCCGGCAGCGGCTACGGCGTCGCGCAGTTCCTGC
TCTACGCGTCCTATGCGCTGGGGCTCTGGTACGCGGCGTGGCTGGTGAAGCACGGCGCCG
CTGACTTCTCGCGCGCCATCCGCGTCTTCATGGTGCTCATGGTGTCCGCCAACGGCGCCG
CCGAGACGCTCACGCTGGCCCCGGACTTCGTCAAGGGCGGCCGCGCCATGCGCTCCGTGT
TCGAGACCATCGACCGGCGCACGGAGGTGGAGCCCGACGACGCGGACGCGGCGCCCGTGC
CGGAGCGGCCGCGCGGCGAGGTGGAGCTCCGGCACGTGGACTTCGCCTACCCGTTGCGGC
CGGAGGTGCCGGTGCTCCGGGACCTGAGCCTCCGGGCGCGCGCCGGCAGGACGCTGGCGC
TGGTGGGGCCGAGCGGGTGCGGCAAGAGCTCCGTGCTGGCGCTGGTGCAGCGCTTCTACG
AGCCCACCTCCGGGCGCGTGCTCCTGGACGGCAGGGACGTGCGCAAGTACAACCTGCGGG
CGCTGCGGCGCGCCATGGCGGTGGTGCCGCAGGAGCCCTGCCTGTTCGCGGCGAGCATCC
ACGACAACATCGCCTACGGGCGTGAGGGCGCGACGGAGGCGGAGGTGCTGGAGGCGGCGG
CGCAGGCCAACGCCCACAGTTTCATCTCGGCGCTGCCGGAGGGGTACCGGACGCAGGTCG
GGGAGCGCGGGGTGCAGCTGTCGGGCGGCCAGCGGCAGCGGATCGCCCTCGCGCGCGCGC
TGGTGAGGCAGGCGCCCATCCTGCTGCTGGACGAGGCCACCAGCGCGCTGGACGCAGAGT
CGGAGCGCTGCGTGCAGGAGGCGCTGGAgcgcgcgggggccggggccggggccggcggcc
gcggcACCACCACCATCGTCGTCGCGCACCGCCTCACGACCGTGCGCAACGCCCACACCA
TCGCCGTCATCGACGACGGCAAGGTGATGGAGCAGGGCTCGCACGCG
>Dw3_sg0contig03796_3522-6032
AGAAGCAGCGCATCGCCATCTCGAGAGCGATTCTGAAGAACCCGTCGATACTGCTCCTCG
ACGAGGCGACCAGCGCGCTGGACGCCGAGTCCGAGAAGAGCGTCCAGGAGGCGCTGGACC
GCGTCATGGTCGGCCGCACCACCGTGGTGATCGCGCACCGCCTGTCCACCATCCGGAACG
CCGACACGATCGCCGTCGTGGACGGTGGCCGGATCGTCGAGACTGGCACGCACGAGCAGC
TCATGGCGAACCCGCGCGGCGCGTACTCCTCGCTGATCCAGCTGCAGGAGGCTGCCCAGC
TTCAGCAGAAGCCGTCTTTCTCTGACAGCGCAAGCATCACCAGGCCCGTAAGGTATCACT
CCACTCGATCATCAAATGGTGAACACTGTTGTCCGGGAGTCAACATGATCTGATGAACTA
AAACTTGTTTGCTTGTAGTTTGAAGTATTCAAGGGAGTTGTCCGGGAGGACGAGCATGGG
TGCCAGCTTCCGTTCCGACAAGGACTCCATCAGCCGGTACGGCGCCGCCGAGGCGCACGA
GGAAGCGCGCAAGGGGAAGCCCGTGTCCATGAGGAAGCTCTACTCCATGGTGCGGCCGGA
TTGGTTCTTCGGCGTGTCGGGCACCCTCAGCGCCTTCGCGGCGGGCTCCCAGATGCCGCT
CTTCGCGCTGGGCGTCACGCAGGCGCTGGTGTCCTATTACATGGGGTGGGAGACCACCAA
GCAGGAGGTCCGCAAGATCTCCGTGCTCTTCTGCTGCGGCGCCGTGCTGACGCTGGTGTT
CCACGTCGTCGAGCACCTCAGCTTCGGCATCATGGGCGAGCGGCTCACGCTGCGGGTCCG
GGAGAGGATGTTCTCGGCGATCCTGCGGAACGAGATCGGGTGGTTCGACGACACCAGCAA
CACCAGCGCCATGCTGTCGTCGCGGCTCGAGGCGGACGCCACGCTCGTGCGCACCATCGT
CGTCGACCGCTCCACCATCCTGCTGCAGAACGTCGGCATGATCGTGACCTCGCTCATCAT
CGCCTTCATACTCAACTGGAGGATCACGCTGGTCGTGCTCGCAACCTACCCCCTCATGGT
CAGCGGTCACATCAGTGAGGTGAACTAACACAGTAACACACTGTCTTCTCAGTAGCAAAT
TGACGAGTCGATCATTGATTTCTCCTCAAATTCACGATCTCCTGTGCTTCTTTTGTTCTG

AAACAGAAAATGTTCATGAAAGGTTACGGCGGCAACcTCGGCAAGTCGTATCTGAAGGCC
AACATGCTCGCCGCGGAGGCAGTGAGCAACATCCGGACGGTGGCAGCCTTCTGCTCGGAG
GAGAAGGTGATAAAGCTCTACGCGGACGAGCTGAAGGAGCCATCGAAGAGGTCCTTCCGG
CGAGGCCAGGGCGCCGGTCTGTTCTATGGAGTTTCTCAGTTCTTCCTCTTCTCTTCATAC
GCACTGGCGTTATGGTAACTGCCACTTCTTACGCAGACTGAATTCAATCTTTTTtACTTT
TtAGTTGAAAAGTAAAaTAAAATGTTACGAATTTCAGGTATGGTTCGCATCTGATGAGCA
AGGAGCTGGCCACCTTCAAGTCCGTGATGAAGTCCTTCATGGTGCTCATAGTGACGGCGC
TGGCCATGGGGGAGACGCTGGCGATGGCGCCGGACATCATCAAAGGCAACCAGATGGTGT
CCTCGGTGTTCGATATCCTGGACCGCAAGACCGACGTCCAGATCGACGCAGGCGAGGACA
TCAAGAGGGTGGAGGGGCTAATCGAGCTGCGCGGCGTCGAGTTCCGGTACCCGTCGAGGT
CCGACGTGACCGTGTTCAAGGGCCTCGACCTCCTGATGAAGGCCGGCAGGAGCATGGCGC
TCGTCGGAATGAGCGGCTCCGGCAAGAGCACCGTGCTGTCGCTCATCCTCCGGTTCTACG
ACCCAGTCGCCGGAAGAATCTTGATCGACGGTACCTCTTGCTCAAAGACAATAGCTCCAT
TTTTTTCCAGAAAAATTCTTCTGCTAAGGCCACTGTACGTTGATCCAATTTGCAGGGAAG
GACATCAGGAAGCTTAAGCTGAAGTCGCTGAGGAAGCACATCGGCCTGGTCCAGCAAGAG
CCGGCGCTGTTCGCGACGACGATCTACGAGAACATCCTGTACGGCAAGGACGGGGCGACG
GAGGCCGAGGTGATCGAGGCGGCGACGCTGGCGAACGCGCACTCGTTCATCAGCTCGCTG
CCGGAGGGGTACCAGACCAAGGTCGGGGAGCGCGGCGTGCAGCTGTCCGGCGGGCAGAAG
CAGCGCATCGCGATCGCGCGCGCCATCGTCAAGGACCCGGCCATCCTGCTCCTGGACGAG
GCGACGAGCGCGCTGGACGTGGAGTCGGAGCGCGTCGTGCAGCAGGCGCTGGACCGGGTG
ATGAAGAACCGGACCACCGTGATGGTGGCGCACCGGCTGTCGACGATCAAGAACGCCGAC
GTCATCTCGGTGCTGCAGGACGGCAAGATCATCGAGCAGGGGGCACACCAG
>Dw3_sg0contig18476_9040-7900
GCAGCAACTGTCGATGAAGGGCTTCGCGGGGGACACCGCCAAGGCGCACGCCAAGACGAG
CATGATCGCCgGGGAGGGCGTGAGCAACATCCGCACGGTgGCGGCCTTCAACGCGCAGGA
CAAGATCCTGTCCCTCTTCTGCGGCGAGCTGCGCGTCCCGCAGGCGCACAGCCTGCGCCG
CAGCCAGGTCTCgGGCGCGCTCTTCGGCCTCTCCCAGCTCTCCCTCTACGCCTCCGAGGC
GCTCATCCTCTGGTTCGGCGCGCACCTCGTCCGCGCCCGCGCCTCCACCTTCTCCAAGGT
CATCAAGGTCTTCGTCGTCCTGGTCATCACCGCCAACTCCGTCGCCGAGACCGTCAGCCT
CGCGCCGGAGATCGTGCGCGGCGGGGAGTCCATCCGCTCCGTCTTCGCCATCCTCAACAG
CCGGACGCGCATCGACCCGGACGACCCGGACGCCGAGCAGGTGGAGTCGGTGCGCGGCGA
GATCGACTTCCGCCACGTCGACTTCGCGTACCCGACCCGCCCCGACGTGATGGTCTTCAA
GGACCTGAGCCTGCGGATCCGGGCCGGGCAGAGCCAGGCcCTGGTCGGCGCGAGCGGGTC
CGGGAAGAGCACCGTCATCGCCCTCATCGAGCGCTTCTACGACCCGCTCGCCGGCAAGGT
GATGCTCGACGGCAAGGACATCCGCCGGCTCAACCTCAAGTCCCTGCGCCGCCGCATCGG
GCTGGTGCAGCAGGAGCCCGTGCTGTTCGCCACCAGCATCCTGGAGAACATCGCCTACGG
CAGGGACGGCGGCGCGACGGAGGAGGAGGTCGTGGAGGCCGCCAAGGCGGCCAACGTGCA
CGGCTTCGTCAGCGCGCTCCCCGACGGGTACCGCACCCCCGTCGGCGAGCGCGGcGTGCA
GCTGTCCGGCGGGCAGAAGCAGCGCATCGCCATCGCGCGCGCGGTGCTCAAGGaCCCCGc
CGTGCTGCTGCTGGACGAGGCCACCAGCGCGCTGGACGCCGAGTCCGAGTGCGTGCTGCA
GGaGGCGCTGGAgCGCATCATGAAgaGCCGCACCGCCGTGCTGGTGGCGCACCGGCTCTC
CAcCATCCGGGGCgTCGACTCCATCGCCGTCGTGCAGGACGGCCGCGTCGTCGAGCAGGG
G
>Dw3_sg0contig26408_1683-2829r

GCAGCAACTGTCGATGAAGGGCTTCGCGGGcgACACGGCCAAGGCGCACGCCAAGaCGAG
CATGATCgCCgGGGAGGGCGTGAGCAACATCCGCACGGTGGCGGCCTTCAACGCGCAGGA
CAAGGTCCTGTCCCTCTTCTGCgGCGAGCTGCGCGTCCCGCAGGCGCACAGCCTCCGCCG
CAGCCAGGTCTCGGGCGCGCTCTTCGGCCTCTCCCAGCTCGCCCTCTACGCCTCCGAGGC
GCTCATCCTCTGGTTCGGCGCGCACCTCGTCCGCGCCCGCGCCTCCACCTTCTCCCGGGT
CATCAAGGTCTTCGTCGTCCTCGTCATCACCGCCAACTCCGTCGCCGAGACCGTCAGCCT
CGCGCCGGAGATCGCGCGCGGCGGGGAGTCCATCCGCTCCGTCTTCGCCATCCTCAACAG
CCGGACGCGCATCGACCCGGACGACCCGGACGCGGAGCCGGTGGAgGCaGTGCGCGGCGA
CATCGACCTCCGGCACGTCGACTTCGCGTACCCGACGCGCCCCGACGTGATGGTGTTCAA
GGATTTCAGCCTGCGGATCCGGGCCGGaCAGAGCCAGGCGCTGGTCGGCGCGAGCGGGTC
CGGGAAGAGCACCGTCATCGCCCTCATCGAGCGCTTCTACGACCCGCTCGCCGGCAAGGT
GATGCTCGACGGCAAGGACATCCGGCGGCTGAACCTCCGGTCCCTGcGCCGCCGCATCGG
GCTGGTGCAGCAGGAGCCCGTGCTGTTCGCCGCCAGCATCATGGAGAACATCGCCTACGG
caGGGAGGACGGCGCGACGGAGGAGGAGGTCGTGGAGGCCGCCAAGGCCGCCAACGTGCA
CGGCTTCGTCAGCGCGCTGCCCGACGGGTACCGCACCCCCGTCGGGGAGCGCGGCGTGCA
GCtgTCCGGCGGGCAGAAGCAGCGCATCGCCATCGCGCGCGCGGTGCTCAAGGACCCCGc
CGTGCTGCTGCTGGACGAGGCCACCAGCGCGCTGGACGCCGAGTCCGAGTGCGTGCTGCA
AGAGGCGCTGGAGCGCATCATGAAGGGCCGCACCGCCGTGCTGGTGGCGCACCGGCTCTC
CACCATCCGCGGCGTCGACTCCATCGCCGTCGTGCAGGACgGcCGCGTGGTGGAGCAGGG
GACGCAC
>Dw3_sg0contig43647_2891-5451
GCGGGCAGAAGCAGCGCATCGCCATCTCGAGGGCCATCCTGAAGAACCCGTCGGTGCTGC
TGCTCGACGAGGCGACCAGCGCGCTGGACGCCGAGTCCGAGAAGAGCGTCCAGGAGGCGC
TGGACCGCGTCATGGTCGGCCGCACCACCGTGGTGAtcGCGCACCGCCTGTCCACCATCC
GGAACGCCGACACGATCGCCGTCGTGGACGGTGGCCGGATCGTCGAGACGGGCACGCACG
AGCAGCTCATGGcGAACCCGTGCGGCGCCTACTCCTCGCTGATCCAGCTGCAGGAGGCTG
CCCAGCTTCAGCAGAAGCCGTCTTTGtCTGACAGCGCAAGCATCACCAGGCCACTAAGGT
ATCACTCTGCTCTCCACTGGATGGATCGTCAAATGGCGAACACTGTCCAAGATTTCAAAT
GTGTCATTGTGATCTGATGAACTGAGCTTGTTTTTtCGTAGTTTCAAGTATTCGAGGGAG
TTGTCCGGGAGGACGAGCATGGGTGCCAGCTTCCGTTCCGACAAGGACTCCATCAGCCGG
TACGGCGCCGCCGAGGCGCACGAGGAAGCCCGGAAGGGGAAGCCCGTGTCCATGGCGAAG
CTCTACTCCATGGTGCGGCCGGACTGGTTCTTCGGCGTGTCGGGCACCCTCAGCGCCTTC
GTGGCGGGCTCCCAGATGCCGCTCTTCGCGCTAGGCGTCACGCAGGCGCTGGTGTCCTAC
TACATGGGGTGGGAGACCACCAAGCGGGAGGTCCGCAAGATCTCCGTGCTCTTCTGCTGC
GGCGccGTGCTCACGCTGGTGTTCCACGTCGTCGAGCACCTCAGCTTCGGCATCATGGGC
GAGCGGCTCACGCTGCGGGTCCGGGAGAGGATGTTCTCGGCGATCCTGCGGAACGAGATC
GCGTGGTTCGACGACACCAGCAGCACCAGCGCGATGCTGTCGTCGCGGCTCGAGGCGGAC
GCCACGCTCGTGCGCACCATCGTCGTCGACCGCTCCACCATCCTGCTGCAGAACGTCGGC
ATGATCGTGACCTCGCTCGTCATCGCCTTCATACTCAACTGGAGGATCACGCTGGTCGTG
CTCGCAACCTACCCCCTCATGGTCAGCGGCCACATCAGCGAGGTGAATTAACAAAGCAAC
GTCTCCTGCTCACCCTGTTATCAGTAGCAAATTGACAAGTCGATCATTGTTTTTCTCCTC
AAATTCACGATCTCCTGTTGTGCTTGTTCTGTTCTGAAACAGAAAATGTTCATGAAAGGT
TACGGCGGcAACCTCGGCAAGTCGTATCTGAAGGCCAACATGCTCGCGGCGGAGGCGGTG
AGCAACATCCGGACGGTGGCAGCCTTCTGCTCGGAGGAGAAGGTGATAAAGCTCTACGCG

GACGAGcTGAAGGAGCCATCGAAGAGGTCCTTCCGGCGAGGCCAGGGCGCCGGCCTGTTC
TATGGAGTTTCTCAGTTCTTCCTCTTCTCTTCATACGCACTGGCTTTATGGTAACTGCCA
CTTCTTATGCACACTGGATTCAATACTTTTACTACCTTTTAGTTGAAAAGTAAAaTAAAA
TGTTACAAATTTCAGGTATGGTTCGCATCTGATGAGCAAGGAGCTGGCCACCTTCAAGTC
CGTGATGAAGTCCTTCATGGTGCTCATAGTGACGGCGCTGGCCATGGGTGAGACGCTGGC
GATGGCGCCGGACATCATCAAGGGGAACCAGATGGTGTCCTCGGTGTTCGATATCCTGGA
CCGCAAGACCGACGTCCAGATCGACGCCGGCGAGGACATCAAGAGGGTCGAGGGGCTGAT
CGAGCTGCGCGGCGTCGAGTTCCGGTACCCATCGAGGCCCGACGTGACCGTGTTCAAGGG
CCTCGACCTCCTGATGAAGGCCGGCAGGAGCATGGCGCTCGTCGGAATGAGCGGCTCCGG
CAAGAGCACCGTGCTGTCGCTCATCCTCCGGTTCTACGACCCGGTCGCCGGAAGAATCTT
GATCGACGGTACCTCTTGCTCAAAGACGACAATAGCGCCATTTTTTTTtGTTCCAGATAA
ATTCTTCTACTAAGGGCAaTGTACGCTGATCCAATCAATTCGCAGGGAAGGACATCAGGA
AGCTCAAGCTGAAGTCGCTGAGGAAGCACATCGGCCTGGTCCAGCAAGAGCCGGCGCTGT
TCGCGACGACGATCTACGACAACATCCTGTACGGCAAGGACGGGGCGACGGAGGCCGAGG
TGATCGAGGCGGCGACGCTGGCGAACGCGCACGCGTTCATCAGCTCGCTGCCGGAGGGGT
ACCAGACCAAGGTCGGGGAGCGCGGCGTGCAGCTGTCCGGCGGGCAGAAGCAGCGCATCG
CGATCGCGCGCGCCATCGTCAAGGACCCGGCCATCCTGCTCCTGGACGAGGCGACGAGCG
CGCTGGACGTGGAGTCGGAGCGCGTCGTGCAGCAGGCGCTGGACCGGGTGATGAGGAGCC
GGACCACCGTCATGGTGGCGCACCGGCTGTCGACGATCAAGAACGCCGACGTCATCTCGG
TGCTGCAGGACGGCAAGATCGTCGAGCAGGGGGCGCACCAG
>FLD_*sg0contig01920_13106-15435
CCTTTCCTGCCAACTAATTTCACCCCCATTTTTTCACAGAGGTAATGCAACCTCATGTCA
TCACCTCCTCTAACTTCTCTTAACTCCATAGCTTGCTGTCGTGATAGCAATGTGTAAACA
TAAAGTTGTTGTTGCTGGTTAAAATGTGACTGGAGCTGCTGGAACAACAATTTGTTTGAG
TGGTTCTGTTCTGTCTTCGCACCTTCAGTTGCATTCTTCTTTCGCTGGCCACCTAATTCC
ACCTTTAGAATAGCTGGGGATTTTGGGTTGGAAGCTTTTCCTCCGAAAATAACAGAGAAG
CTCCCAAACTCCAAATCCGGTTGCCTAAACAAGTCCGTTAGTATAGTAGCACAAGCCTGT
GTGTTTGTTGACGAGCTTCTGTCCACTTTTGTCTTCGCTACTCGGGCATTGGCATGGAGT
GTGATATTAGCAGCCTCCCGCAAACCACTGATAAAAGCACCATGCATTGTTGCTGGGTAA
CGTCTCGTGGTAGCCTCGCCAGCAAAGAAAAGTCGTCCATCACCAACACTTTCAGCTAAT
ATGTCATAGTCATCTCCAGATGCTCCGACAGCAACATGAGAATATGAACCTAGACTGAAG
GAGTCAGTGCCCCATCTAGTGCAAACACTCTGTAGAGGATCTGGAACCTCAATCCCTTGT
GGTTCATAGATACCTGTGGACACAAGGTGCATATTTTAGAGGAAAATCCAGGAGTTTAAG
CAGTCTATCCAGTTATATGACACGGACATAACTGTATATACTTCAGGATACTGGAACTGA
ATAGAATAAAGACCAAGTTTTGAAAGTAACTCCTGGTTTAGTGAGAGGTGTTAAAATACT
ACATTATGCAGAGTCGCTTCTATAATGACGCAAGGTTTGATTTTATTTATTTTTTTGGAA
AATCAAACTTTGATTGTAGAAATTATATTCTCACCTCGTAGTATCTGAAGAACTGAGCTG
ACAGCATCAGTTGGCGGTGTGGTTTCAAAATTGTGAGCAGCTTCACCAGCAACCAAGGCC
ATCAACAATGGGCCACTAGCCACTGTCGCATAGCTATAGAACAGAAAGAACTCCCCACGT
CGCCTTGGATCTTCAACCAGATGCCCAAATGTATCAAGGTCAGTGCTCCAGAAAACATGC
GGAAACAGCATGGCTACCTTGTTCAATAACCCGAATCCAAGCCTCTTAATACTATCAAGC
TTCCTCTGTGGCAACTCAGGTACAAACTTGATGCCTCCATTTTTCAGAACCCCAAGTGGA
ACTGTGCACAGCGCCATGTCCCCTTCATACACCTGCCCTCCATTGACAACCACCTGCACC
CCATCCCCTCCATATCGTACAGTGTGCACCGTCCTCTCGTAAACAATCGGCACATTCTCT

GCCAGGGCTTGCACAAGTCTCCCATTGCCACCAGGCAAGAAGCAGTGATCCCCACCCATG
TCATAGGGATCATCCTGGTCCCAGAACGCAAGGCTCAACTTCGACAACAATCCCGCATTG
GCATACTCGAGATTTGCTAGATGCCAATTGAACAAGTTCTTCTCCTCCTGTGTGGATATG
CCTCCGTCTGTCTGGCGTAATGTCTCAAGAGCAGCCCCGAGCGAAACATCTACTGCCACC
TCCCCCATGGATGACCGCAGGTTGCTTGACTTATCAAGAAGCTTGTTAAATGTAATCTCT
ACTTTCTTATCCACCTCCGGGTCGACTGGTGATCCATCCGGACGGTAGAGTGGGCACTTG
TCCCTAATCTTGTGCATTGGCAAGCCGAGCTGCTTCGCCACAATTCCCAGTGGGTTGCCA
AACGTCCCGGTAAGCACGCTGCCCCCAAGATCACCAGCAGCTGAACGCCCACTGCCTTCC
ATCTTCTTCGTGTACACACGGCCACCGCAGCGCTTGCGGCCCTCCAAGACTATAACCTTA
AACCCGAACGCCACAAGCTGCCGTGCCGCGGCGAGCCCGGCAAGGCCTGCTCCAATGACG
ACGATGGTGGTAGGCCTCGTGGGCTCCTTGGGGATGCGCTCCTTGATGGCCGGCGCGACA
CCGAAGTTAACGTAGCTGTGGGACACAAGGAAGGAGTAGGCGGCGTTCAGGAGGTGCTCG
CAATGGGGAGGGATGAGCGTCGCGAAGGGCTCCTTGGCGAGCCAGGAGTTGTAGGTCTCG
CGCCACCGGCAGAGGAGGTGGTTGCGGATGAGGATGTAGTTGACCTGCTCGATGCCGCCC
ACGTCGGAGACCACGCCAGCCTCGATCTCCTCGTCGGTGAGAGAGTCCGCCGGGAAGCCC
GCGGTGAGCGCGGTGACGGCCTCCGCGGTAGGCTCCCGGTTGATCACTAT
>FLD_*sg0contig102960_2518-190
CCTTTCCTGCCAACTAATTTCACCCCCATTTTTTCACAGAGGTGATGCAACCTCATGTCA
TCACCTCCTCTAACTTCTCTTAACTCCATAGCTTGCTGTCGTGATAGCAACGTGTAAACA
AAAAGTTGCTGTTGCTGGTTAAAATGTGACTGGAGCTGCTGGAACAACAATTTGTTTGAG
TGGTTCTGTTCTGTCTTTGCACCTTCAGTTGCATTCTTCTTTCGCTGGCCACCTAATTCC
ACCTTTAGAATAGCTGGGGATTTTGGGTCAGAAGCTTTTCCTCCGAAAATAACAGAGAAG
CTCCCAAACTCCAAATCTGGTTGCCTAAACAAGTCCGTTAGTATAGTAGCACAAGCCTGT
GTGTTTGTTGACAAGCTTCTGTCCACTTTAGTCTTTGCTACTCGGGCATTGGCATGGACT
GTGATGTTAGCAGCCTCCCGCAATCCACTGATAAAAGCACCATGCATTGTTGCTGGGTAA
CGTCTCGTGGTAGCCTCGCCAGCAAAGAAAAGTCGTCCATCCCCAACACTTTCAGCTAAT
ATGTCATAGTCATCTCCAGATGCTCCGACAGCAACATGAGAATATGAACCTAGACTGAAG
GAGTCAGTGCCCCATCTAGTGCAAACACTCTGTAGAGGATCTGGAACCTCAATCCCTTGT
GGTTCATAGATACCTGTGGACACAAGGTGCATATTTTAGAGAAAAATCCAGGACTTTAAG
CAATCTATCCAGTTATATGACACGGACATAACTGTATATACTTCAGGATACCTGTGGAAG
TGAATAGAATACAGAACAAGTTTTGAAAGTAACTCCTGGTTTAGTGAGAGGTGTTAAAAT
ACTACATTATGCAGAGTCGCTTCTATAATGATGCAAGGTTCAATTTTATTTTTGAATAAA
ATCAAACTTTTATTGTAGAAATTATATTCTCACCTCTTAGTATCTGAAGAACTGAGCTGA
CAGCACCAGTTGGCGGTGTGGTTTCAAAATCGTGAGCAGCTTCACCAGCAACCAAGGCCA
TCAACAATGGGCCACCAGCCACTGTCGCATAGCTATAGAACAGAAAGAACTCCCCACGTC
GCCTTGGATCTTCAACCAGATGCCCAAATGTATCAAGGTCAGTGCTCCAAAAAACATGTG
GAAACAGCATGGCTACCTTGTTCAATAACCCGAATCCAAGCCTCTTAATACTATCAAGCT
TCCTCTGTGGCAACTCAGGTACAAACTTGATGCCTCCATTTTTCAGAACCCCAAGTGGAA
CTGTGCACAGTGCCATGTCCCCTTCATACACCTGCCCTCCATTGACAACCACCTGCACCC
CATCCCCTCCATATCGTACAGTGTGCACCGTCCTCTCGTAAACAATCGGCACATTCTCTG
CCAGGGCTTGCACAAGTCTCCCATTGCCACCAGGCAAGAAGCAGTGATCCCCACCCATGT
CATATGGATCATCCTGGTCCCAGAACGCAAGGCTCAACCTCGACAACAATCCGGCATTGG
CATACTCGAGATTTGCTAGATGCCAATTGAACAAGTTCTTCTCCTCCTGTGTGGATATGC
CTCCATCTGTCTGGCGTAATGTCTCGAGGGCAGCCCCGAGCGAAACATCCACTGCCACCT

CCCCCATGGATGACCGCAGGTTGCTTGACTTATCAAGAAGCATGTTAAATGTAATCTCTA
CTTTCTTATCCACCTCTGGGTCAACTGGTGATCCATCCGGACGGTAGAGTGGGCACTTGT
CTCTAATTTTGTGCATTGGCAAGCCGAGCTGCTTCGCCACAATTCCCAGCGGGTTGCCGA
ACGTCCCGGTAAGCACGCTGCCCCCGAGATCACCAGCAGCTGAACGCCCACTGCCTTCCA
TCTTCTTCGTGTACACGCGGCCACCGCAGCGCTTGCGGCCCTCCAAGACTATGACCTTAA
ACCCGAACGCCACCAGCTGCCGTGCCGCGGCGAGCCCGGCAAGGCCCGCACCGATGACGA
CGACGGTGGTAGGCCTCGTGGGCTCCTTGGGGATGCGCTCCTTGATGGCCGGCGCGACAC
CGAAGTTAACGTAGCTGTGGGACACGAGGAAGGAGTAGGCGGCGTTCAGGAGGTGCTCGC
AGTGGGGAGGGATGAGCGTCGCGAAGGGCTCCTTGGCGAGCCAGGAGTTGTAGGTCTCGC
GCCACCGGCAGAGGAGGTGGTTGCGGATGAGGATGTAGTTGACCTGCTCGATGCCGCCCA
CGTCGGAGACCACGCCAGCCTCGATCTCCTCGTCGGTGAGAGAGTCCGCCGGGAAGCCCG
CGGTGAGCGCGGTGACGGCCTCCGCGGTAGGCTCCCGGTTGATCACTAT
>FLD_sg0contig16503_1111-2769r
ATGTTAGCAGCCTCCCTATACCCACTAAGCAGGGCTCCATGCATTGTAGCGGGGTATCGC
CTATTTGTTGCCTCCCCAGCAAAGAAAACTCTATCATGCACACTCTCAGCCAAAATGTCA
TAGTCATCACCAGAAGCGCCAATAGCCACATATGAGTATGATCCATAAGTAAACCTGTCG
GTACCCCATCGAGAGCATATTGCTTGCAATGGGTTTGGAACTTCAATCCCCTTGGGAGAA
AAGATTTTCCTAAGTGTTGCTAGCACCTTCTCAACATTTTCCATTGGTGAAGCCTGCTCA
AACTTGACTGCAGATTCCCCAGCAACAAGGGCAATGAGCAATGGCCCTCCTGAGACAGAA
GAATAGCTGTAGAACAGGAAGAACTCGCCACGCTGACAAGAATCTTCTGTCAAGTGACCA
AATGTATCAATCCTACCATCCCAGAAATCATAAGGGAACAGCATCATAACCTTGTTAAGC
AGACCAAAACCCAATCTCTGGATGGCCTCTTTCTTCTGAGCAGGTAGCTCAGGCACAAAC
TTGATGTCACCCTTCTTGAGCACGCCAAGAGGAACTGTACAGAGAACCATATCACCACGA
AACGACTGCTTATCAGTGTGCACCATCACACCATCACGCCCATACTGTATCTTTTGCACA
TTCTGCCCATAGAATATCGGGATGCCATCAGCAAGTGCACGGACAAACTGAGAATTTCCA
CCAGGAATAAAGCAGTGGTCTCCGCCCATTTCATACGGATCATCCTGGTCCCAGAAGGCC
ATGGAGAGATCAGCAAGAGGGGCAGCATTAGCATACTCCAGGTTCGCCAAATGCCAGTCC
AGAAGCATCCTTTCTTCATGCTCAGCTGCAACACCATGCGCTGCTCGGAATGCCTCAAGT
GCCATGCCCAGTGACAAATCAACACCATGTGGCACACCATCTGCAATCACCTGCCTCAAC
TGGCAAACCTTGTCCAGAAGCTGATTAAAAGCAGCCTCAACACGAGCATCCATGTCGGGG
TCAACTGGGCGGCCATCTGGAAGATACAGTGGGCATTTGTCCCGCACTTTGTGGAGCGGG
AATCCAAGCTGCCGTGCAATGACACCAAGGGGATTCCCATTGATACCGGTGAGCACGCTG
CCTCCAAGGTCAGCAGCAGCAACTGTATCAGGATACTCTGCAGCAGATGAACACATTGTC
TTGGTAAACaCACGGCCGCCGGGTCGTAGTCGACCTTCGATGATGGCCACCTTGAAGCCT
AGTGCTATAAGGTGGCGCGCGGCGCCCAGACCAGCAAGGCCAGCGCCAATAAtgAGGACC
GATGGGGGAGGAAAGGATGGAGGAGACCGTgGGGGGAGCGAGAGGACGGCgGGGGCGAGC
CCGAAATTGATGTACGCGTGCTCGGACAgGAAGGAATGCGCCGCGGCAACAAGGGGCGCG
TGCTCAGCGCGAATaGAAGCAAGCGCGGCGTTGGGTGCGACTGGGGAAAgGGGATTAGAG
CGCCAGAGCGCGACGATGTGGTTGCGAACGACGAGGTAGTTGGCCTGCTCGGCGCCGCCA
ATGCGTGGGAGCACAGCGGCAACGATCTCGTCCTCGGAGAGGGAGTCCGCGGGGAACCCC
GCGGCGAGAGCGATGAGGGCCTCGGAGTCGAGCTCCCGG
>FLD_sg0contig114184_653-1198r
TTTCAAACTCCAAAGCAGCCTCTCCAGCCACAAGTGCAATAAGCACTGCTCCGCCAGAGA
CAGTGTGGTAGCTATAGAACAGAAAGTACTCCCCACGCTTGCTACTCTCCTTGTTCAAAC

ACCCAAATGTATCGATATCCTCATCCCAGAACATGTGAGGAAAgACCATGGCCACCTTGT
TCAGCAACCCAAACCCCAACCTCTGTATTGCCCCAAGCTTATGCTCAGGCAACTCTGGGT
CGAACACGATGCTACCACTCTTGAGCACCCCAAGGGGTACAGTGCACAAAACCATGTCAG
CCTGGAAAACCTGCCCTCCTTCCACTGTGACGCTAACCCCATCGGATCCATGCTCAATTC
GCATCACCGTCTTCTCATACaACACgGGCACACCATCACaCAAtgCATGTATGAgCCgGG
AATTcCCTCCagCTAGGAAGCAATGATcTCCCCCCATCTCATAAGGATCGTCTTGGTCCC
AATGTGCGAGTGAGAGCTCTGACAGGCAACCAGCATTGGAGAATTCCAGATTCGCCAAAT
GCCAAT
>FLD_sg0contig20763_3886-5544r
ATGTTAGCAGCCTCCCTATACCCACTAAGcAGGGCTCCATGCATTGTAGCGGGGTATCGC
CTATTTGTTGCCTCCCCAGCAAAGAAAACTCTATCATGCACACTCTCAGCCAAAATGTCA
TAGTCATCACCAGAAGCGCCAATAGCCACATATGAGTATGATCCATAAGTAAACCTGTCG
GTACCCCATCGAGTGCATATTGCCTGCAATGGGTTTGGAACTTCAATCCCCTTGGGAGAA
AAGATTTTCCTAAGTGTTGCCAGCACCTTCTCAACaTTTTCCATTGGTGAAGCCTGCTCA
AACTTGACTGCaGATTCCCCAGCAACAAGGGCAATGAGCAATGGCCCTCCTGAGACAGAA
GAATAGCTGTAGAACAGGAAGAACTCGCCACGCTGACTAGAGTCTTCTGTCAAGTGACCA
AATGTATCAATCCTACCATCCCAGAAATCATAAGGGAACAGCATCACAACCTTGTTAAGC
AGACCAAAACCCAATCTCTGGATGGCCTCTTTCTTCTGAGCGGGTAGCTCAGGCACAAAC
TTGATGTCACCCTTCTTGAGCACGCCAAGAGGAACTGTACAGAgAACCATATCACCACGA
AACGACTGCTTATCAGTGTGCACCATCACACCATCACGCCCATACTGTATCCTTTGCACA
TTCTGTCCATAGAATATCGGGATGCCATCAGCAAGTGCGCGGACAAACTGAGAATTTCCA
CCAGGAATAAAGCAGTGGTCTCCACCCATTTCATACGGATCATCCTGGTCCCAGAAGGCC
ATGGAGAGATCAGCAAGAGGGGCAGCATTAGCATACTCCAGGTTCGCCAGATGCCAGTCC
AGAAGCATCCTTTCTTCATGCTCAGCTGCAACACCATGCGCTGCTCGAAATGCCTCAAGC
GCCATGCCCAGTGACAAATCAACACCATGTGGCACACCATCTGCAATCACCTGCCTCAGC
TGGCAAACCTTGTCCAGAAGCTGGTTAAAAGCAGCCTCAACACGAGCATCCATGTCGGGA
TCAACTGGACGGCCATCTGGAAGATACAGTGGGCATTTGTCCCGCACTTTGTGGAGCGGG
AATCCAAGCTGCCGTGCAATGACACCAAGGGGGTTCCCATTGATACCGGTGAGCACGCTG
CCTCCAAGGTCAGCAGCAGCAACTGTATCGGGATACTCTGCAGCAGATGAACGCATTGTC
TTGGTAAACACACGGCCGCCGGGTCGTAGTCGACCTTCGACGATGGCCACCTTGAAGCCT
AGTGCTATAAGGTGGCGCGCGGCGcCCAGACCAGCAAGGCCAGCGCCAATAATTAGGACC
GAAGGCGGAGGATAGGATGGAGGAGACCGTgGGGGGAGCGAGAGGACGGCgGGGGCGAGC
CCGAAATTGATGTACGCGTGCTCGGACAAGAAGGAATGAGCCGCGGCAACAAGGGGCGCG
TGCTCAGCGCGAATGGAAGCAAGCGCGGCGTTGGGTGCGATTGGGGAAAGGGGATTAGAG
CGCCAGAGCGCGACGATGTGATTGCGAACGACGAGGTAGTTGGCCTGCTCGGCGCCGCCA
ATGCGTGGGAGCACGGCGGCAACGATCTCGTCCTCGGAGAGGGAGTCCGCGGGGAACCCC
GCGGCGAGAGCGATGAGGGCCTCGGAGTCGAGCTCCCGG
>FLD_sg0contig269427_753-1298r
TTTCAAACTCCAAAGCAGCCTCTCCAGCCACAAGTGCAATAAGCACTGCTCCGCCAGAGA
CAGTGTGGTAGCTATAGAACAGAAAGTACTCCCCACGCTTGCTACTCTCCTTATTCAGAC
ACCCAAATGTATCGATATCCTCATCCCAGAACATGTGGGGAAACACCATGGCCACCTTGT
TCAGCAACCCAAACCCCAACCTCTGTATTGCCCCAAGCTTATGCTCAGGCAACTCTGGGT
CGAACACAACGCTCCCACTCTTGAGCACCCCAAGGGGGACAGTGCACAAAACCAAGTCAG
CCTGGAAAACCTGCCCTCCTTCCACTGTGACACTAACCCCATCGGCTCCATGCTCAATTC

GCTTCACCGTCTTCTCATACAGCACagGCACACCATCACACAATGCATGTATGAGCCGGG
AATTCCCTCCAGCTAGGAAGCAATGATCTCCCCCCATCTCATAAGGATCGTCTTGGTCCC
AATGTGCGAGCGAGAGCTCTGACAGGCAACCAGCATTGGAGAATTCCAGATTCGCCAAAT
GCCAAT
>FLT_*sg0contig08422_1606-3786
CCGCAGACATATTCTTGTTATGATAGAAAGATTAGGACATTGTATTTCGCAAATCATATT
CTATCAGAAAGCTGGAAGGACGGTCTGCAGTCTCGACAGATATGCCAAAGAGCCAATGTG
CCTCTGTGAGACTTGTTCAGAAGCTACACTAAGAGCTGACCGTCAAGAGATCATATTCCA
TCAGACAAACTAGGAGGGACGTACCGCGTACGCAGTGAGATTGGATTTGCGTAGAAAATT
CACTCTGTTCGTTGTGCTGGTGCTGGTGGCTAGTGCTGGAGtggtatgagagaaaaatac
tgttggctggtatgctgcaataacttctACGTCACAATCCGCACTAACTGCCTCTTGAGG
GCCTGTTGCATAGTAGCATCTCTGATGCTCCAAGTGAATCTTCCAGTGTGCTATTGGGGT
TTCAGTCAGGACTGTTAACCCTAGTGAAGTAAAAGTGCAGCCTAGTGATAGTATGACAGT
AAAAGGTCTACTATTGTCCATATTACTCAGGTGACTATAATGCCACAGACGAGGGGATTA
AGCAGCTTAATTGATAAGGAAACTACATTTAGTTTCAATTTTTCCCGATAACTGAAGAAC
AGAACTTACAGGTTTAAATTACATTATTAAATTTCTAAATAAAGGACAGAAACAGCCATG
GAGAGATGTGTTTGGATAGAAAGATGTGCACAGATAAGAATAGACATACCAGAATAATAT
AAGAAGAAATATATGTCAATATATCACCTTGTCATCATTATCTAAACATAAACACTATTT
TATAATCTTTGGAGGATGAAGAAGACTTGATTCTGAGTTCTAGATGTCGGCATGCACTGA
AATAAGCCATACAGCTGTACCACTAATTTGCTTCCCAAGCATCTACCTTAGTAAAAGTCC
ACTACCAATGAAGAAAGAAATTATTATGTTGGGGGAAGACAGAACAAGTATCAAGAAAAT
GCCTTGACGCCTTTTATGGTTGAAGCCTAATATTCCCAGCAAACAGCGTTTCAGTCAAAG
GAAGTTTTCCACCATTTGTACTGAGAAGGTGATATTCACTACAAGAATGCTATGATGAAT
CTTTAATATGTATGCTCCAGTAAAAAGAAGGCCATGCATTCCTGCATTCACTGAAGGTGG
AACTGGGCACCTGAGCTGATGAGGTTAAACAGTCCTCCCCACCACCCCtgttatggtaga
taaaaccagggcaagataaaccagttcgccctccctcggaggctctaaactatttagggt
ttatattcttgcttgacctcaactgttacaatggtgtctcttttatagagacgactgact
tgacccctaagcaatatatcctaaccgaatcaatctaaCTTGCCATAACACCCCAGGTCA
CTAGCTCATTCCCAAGAATGTATGTTCTAGCCTGTACAAAACAAAGAAATAGAAGAATAC
CAAATACTAAGGGACTTGATGCTTTGGCAGCCCCATTCGCAAAAAACTTTTATTAATGAA
GGGGTGAAATAATGTTCATGCCGCAAACAAAGAAGATGATGTGTCAATCTGATAGACAAG
TCAAAAGGCAACCCACTTTTCACGAGGAACGTATCATGTCTAGGGTTCAAGATATAGGGA
ATAAGTGAGTGTGGTCATCAGATTTTATTCATCAATATGGAATTGCAATGGACACTCCTA
TTTTTGTAAGATCAGATAGTGGACAGGCCTACATATTTCCAGTGATGACGACCAATATCA
TTATATACATTTTAAAGTTGAGATTCTAGGGAATATGGAGCACAACTCCTGATATTATGA
ATGCCTTCAGCACTTCAGGTTGCAAGTATAAATAGGGCATGAACCTGCGCCCATCAGTTC
ACCCAAGTTCACATTTGAGGCCAAGCTAGCTTCTTTGTACCTTATAGCCCAAAGCCTGTA
AGAAAGACTCTAGAGCACAATCTGTTTTCCACCTTGCAACCTCAAATTTATCATTCCTCA
CCAACCATGTCAAGGGACCCACTAGTAGTAGGACATGTCGTTGGTGACATTTTGGATCCA
TTTATCAAATCAGCCTCACTTAGGGTTATGTACAACAATAAGGAACTGACCAATGGATCT
GAGCTCAAGCCGTCACAAGCAGCAAATGAACCAAGGGTCGAAATTGCTGGGCACGACATG
AGGAACCTTTACACTCTGGTA
>FLT_*sg0contig09545_9355-11180
CCGCAGACATATTCTTGTTATGATAGAAAGATTAGGGCATTGTATTTCACAAGTCATATT

CTATCAGAAAGCTGGAAGGACTGTCTGGAGTCTCGACAGATATGCCAATGCGCCTCTGTG
AGAGTTGTTCAGAAGCTACACTAAGAGCTGACCGTCGAGAGATCATATTCCATCAGACGA
ACTAGGAGGGACGTAAGCGGTGAGATTGAATTTGCGTAGAAAATTTACTAGCACCTTATG
CTGCAATAACTTCTACGTCACAATCCGCACTACCTACCTGTTGAGGACCTGCCGCATAGG
AGCATCAGAGATGCTCCAAGTGGTCTTCCAGTGTGCTATTGGAGTTTCAGTCAGGAAGTT
ACCCTAGTGAAGTAAAAGTGCAGCCTAGTGATAGTTATAATTGATAAGGAAACTACATTT
AGTTTCAGATTTTTCTGAAAACTGAAGAACAGAACTTACATGTTTAAATTGCATTATTAG
ATTTCTAAATAAAGGACAGAAACAGCCATGGAGAGATGTGTTTGGATAGAAAGATTTGCA
CAGATAAGAATAGACATACCAGAATAATATAAGAAGAAATATATGTCAATATATCACCTT
GTCACCATTATCTAAACAGAAGCACTATATTTTATAATCGTTGGAGGATGAACAAGACTT
GATTCTGAGTTCTAGATGTCGGCATGCACTGAAATAAGCCACACAGCTGTACCACTAATT
TGCTTCCCAAGCATCTACCTTAGTAAAAAAAGTCCACTACCAATGAAGAAAGAAATTATT
ATGTTGGGGGAAGACAACGAACACGTGTCAAGAAAATCCCTTGACACCTTTTATGGTTGA
AGCCTAATATTCCCAGCAAACAGCGTTTCAGTCAAGGAACTTTTCCGCCATTACTGAGAA
GGTGATATTCACTACAAGAATGCTATAATAAATCTTTAATATATATGCTCCAGTAAAAAG
AAGGCCATGCATTCCTGTATTCACTGAAGGTGGAACTTGGCACCTGAGCTGAGGAGGTTA
AACAGTGAATGTATGTTCCCAAGAATGTATGTTCTAGCCTGTACAAAGTAAAGAAATAGA
AGAATATCAAATACTAAGGGACTTGATGCTTGCCAGCCCCATTCGCAAAAACAACTTTTA
TTAAGGAAGGGCTGAAAGAATGTTCATGCCGCAAACAAAGAAGATGAGGTGTCAATCTGA
TAGACAAGTCAAAATGCAAGCCACTTTTCACGAGGAACGTATCATGTCTAGGGTTCAAGA
TATAGGGAATAAGTGCGTGGTCATCAGATTTTATTCATCAATATGGAATTGCAATGGACA
CTCCTGTTTTTGTAAGCTCAGATAGTGGACAGGCCTACATATTTCCCGTGATGACGACCA
ATATCATTATATACATTTCAAAGTTGGATTCTAGGGAATCTGGAGCACGACTCCTGATAT
CATGAATCCCTTCAGCACTTCAGGTTGCAAGTATAAATAGGGCATGAACCTGCGGCCATC
AGTTCACCCAAGTTCACATTTGAGGCCAAGCTAGCTTCTTTGTACCTTACAGCCCAACGC
CTGTAAGAAAGACTCTAGAGCACAATCTGTTTTTCCCCTTGCAACCTCAAATTTATCATT
CCTCACCAACCATGTCAAGGGACCCACTAGTAGTAGGACATGTCGTTGGCGACATTTTGG
ATCCATTTATCAAATCAGCCTCACTTAGAGTTCTGTACAACAATAAGGAACTGACCAATG
GATCTGAGCTCAAGCCGTCACAAGTAGCAAATGAACCAAGGGTCGAAATTGCTGGGCGCG
ACATGAGGAACCTTTACACTCTGGTA
>FLT_sg0contig18299_5751-6535r
AAATGAAGAAAATAAGGCGTTAGTCTAGGAGATTACAGACAAGTCAAAAGGCAATCTACT
TTTTGGCCATGAACAACTATTTAATAGGTCCTAGATATAGGGAATAAGTGAATGTGATAC
ATCAGATTTTATTCACCTACGTGGAATTGCAGTGAGCACCTATATAACCCCGATACTTCA
ACACGACCCCGTATCACGTATCCGATACGTATCCGCGTATCCGTATCCGATACTCCGATA
CACCTTTGTGCCTTTAACTTTTGCAGAAATTTGACGTATTCACGTATCCGTATCCTTCCG
ATACCGATATGCGTATCCGTATCCGTGCTGCATAGGTGAGCACTCCTTCCATTGGTGTAG
GTGCAGAGGGTGGACAACTCGAAATATTTTCAGTGATGGCAACCCATATCATTCTTTGCA
GTATAAAGCAAGATACCATGGCGTCCAGAGCTAATTTTCAGTCATCACGAATGGTGGCTC
ATTGGGGACCTGCAGTATATATAGAGCACCAGCCTGCACCCTTCACTTCACCCAAGCTCA
TACTTCAGGCCAAGTTAGCTCGTCTTCAAGATTGCCAATCTTAGCTAACCATGTCAAGGG
ACCCACTTGTCGTTGGCAACGTAGTTGGAGATATCTTGGACCCATTTATCAAATCAGCAT
CACTCAGAGTCCTATACaacaaTAGGGAACTCACTAATGGATCTGAGCTCAAGCCATCAC
AAGTAGCCAATGAACCAAGGATTGAGATAGCTGGGCGTGACATGAGGACCCTTTACACTT

TGGTG
>FLT_sg0contig31169_2577-2711
TTGGCCcTCCCTTGGAGGTTCTAAACTATTTAGTGGTTATACTCTTTCTTGCCATCAACT
GATACATGGGTGTCTCTTTTATGGTGATGACTAACTTAACCCCTATTCAAATATCCTAAC
TGACTCATCTAACTT
>FLT_sg0contig34686_1257-1858r
AAACGAAGAAAATAAGGCGTCAGTCTGGGAGATTACAGACAAGTCAAAAGGCAATCTACT
TTTTGGCTATGAACAACTATTTAATAGGTCCTAGATATAGGGAATAAGTGAATGTGATAC
ATCAGATTTTATTCACCTATGTGGAAATGCAGTGAGCACTCCTTCCATTGGTGTAGGTGC
AGAGGGTGGACAACTCGAAATATTTTCAGTGATGGCAACGCATATCATTCTTTGCAGTAT
AAAGCAAGATACCATGGCGTCCAGAGCTAATATTTTCAGTCATCGTGAATGATGCCTCAT
TGGGGACCTGCAGTATATATAGAGCACCAGCCTGCACCCTTCACTTCACCCAAGCTCATA
CGACAGGCAAGTTAGCTCGTGTTCAAGATTGCCAATCTTAGCTAACCATGTCAAGGGACC
CACTTGTCGTTGGCAACGTAGTTGGCGATATCTTGGACCCATTTATCAAATCAGCATCAC
TCAGAGTCCTATACAACAATAGGGAACTCACTAATGGATCTGAGCTCAAGCCGTCACAAG
TAGCCAATGAACCAAGGATTGAGATAGCTGGGCATGACATGAGGACCCTTTACACTTTGG
TG
>FLT_sg0contig241270_448-642r
ATGTCTAGGGACCCATTGGTCGTTGGCCATGTTGTGGGGGATATCGTGGATCCCTTCATC
ACAACAGCATCACTGAGGGTCTTCTACAACAATAAGGAAATGACAAATGGATCTGAGCTT
AAACCATCTCAAGTGATGAGTGAACCAAGGGTCCATATCAGTGGACGTGACATGAGGACC
CTTTACACACTTGTA
>GI1_*sg0contig01489_2837-3966
GGCCATCAGCAACTGATAAACCTGAATCTCCCCATTCAATGATGAGATGGACTGCTCGAG
CGGTTACTTCCAGAAGCTGCATGTATTCATCGGAAGAACGATAGTCAGCAGGCATATGGA
AAATGTAAGTCGATTGGTGTTTAATGACAGCAGAACAGAACAGGAGATTTTGTTAATAGG
ATTCTCCAGTGAAGAAATCTGATGACTGAAAGAGATGTCACCTCAAGTTGTGGCAAAGTA
CAAGCTTCACCGTCAACAAGCATTCCATCAGTAGCACGAAGTAGAAGGTCTGAAGCACTA
GCAAGAATCACCAATGCTTCTGGGCTGTCATGGTTCCTCATAAGCTCAGCTATAAGCTTC
ACTATTCGCTGGTTGACTCTCCACATCTTCTGACCTTGCTCGTCATCTCTAGCAATCCAA
GGCTGAAAGTCCTTCTCGGCCTGCAAAACAAATATGCGTCAAGAAAGATAACAGAGGATA
CGGTACATATTCATGCAGGCGTCATGGTAAAGAAAATGCCTGTTTTGAAATATATTAATT
GAGAAAAAAGAAAACCCAAGTTTTGAACAAAATTTAGTTTTGATCAAACATGCGAATACA
GCAATATCTTGACCTGGAGAACAATAGCAGTTGATGCCTTGGTTGGTGAGGCTGAAACGA
CATCACAAAGCGCATCTACAACCTACACATTAGAAACGAGGATTGTCATTCATCCACAAT
GCTAAGAACAATATAGATAAATTCAGACCGCTATTTACTTATTTAGCTATGTTTATAACT
TATAAGCACACATACATACCTTTCTCCATCCTTGGTGAGCTGACGTACTTTCTGCAGACA
TCTGCATTTCAGGAGAAGCTATGAGCTTCTGCCAGAGCAGTGAGACAACAGAGAAGCACA
GTTCTTGTTTCTCTGACAATACAGATCTTAGGAGAGTTTGAGAACCTCGGTAACCCCCAT
TCCGATCCATGGTAAGGAAGTTTGCCAAATCAGATGCTTCCACTTGTAAGCTTGCAATGG
CTTTCCCAGATGTACTTGCAACATCTCCATTTAGTAGAACCTCCTCTGCACATTTCAAGA
GTGGTCTTGAGAAACCACTTTTCTTATGTGAGGTTAAACCATTCTTGTTC
>GI1_*sg0contig15400_1539-2670
GGCCATCAGCAACTGATAAACCTGAATCTCCCCATTCAATGATGAGATGGACTGCTCGAG

CGGTTACTTCCAGAAGCTGCATGAATTCATTGGAAGAATGATAGTCAGCAGGCATATGGA
AAATGTAAGTCGATTCTGTTCAATGACAGCGGAGCAGAATAGGCGATTTTGTTAATAGGA
TTTTCCAGTGAAGAAATCTGATGACTGAAAGAGATGTCACCTCAAGTTGTGGCAAAGTAC
AAGCTTCACCATCAACAAGCATTCCATCAGTAGCACGAAGTAGAAGGTCTGAAGCACTAG
CAAGAATCACCAATGCTTCTGGGCTGTCATGGTTCCTCATAACCTCAGCTATAAGCTTCA
CTATTCGCTGGTTGACTCTCCACATCTTCTGACCTTGCTCGTCATCTCTAGCAATCCAAG
GCTGCAAGTCCTTCTCGGCCTGTGAAACAAATATGCGTCAAGAAAGATGACAGGATATGG
TACATATTCATGCAGGCGTCATGGTAAAGAAAACGCCTGTCTTGAAGTAAATTAATTGAG
AAAAAATAAAAGAAAAACCAAGTTTTGAACAAAATTTAGTTTTGATCAAACATGCGAATA
CAGCAATATCTGGACCTGGAGAACAATAGCAGTTGAAGCCTTGGTTGGTGAGGCTGAAAC
AACATCACAAAGCGCATCTACAACCTACACATTAGAAACAAGGATTGTCATTCATCCACG
ATGCTAACAACAACATAGATGACTTCATACCGCTATTTACTTATCTACCTATGTTTATAA
CTTATAAGCACACATACATACCTTTCTCCATCCTTGGTGAGCTGACGTACTCTCTGCAGA
CATCTGCATTTCAGGAGAAGCTATGAGCTTCTGCCAGAGCAGTGAGACAACAGAGAAGCA
CAGTTCTTGTTTCTCTGACAGTACAGATCGTAGGAGAGTTTGAGAACCTCGGTAACCCCC
ATTCCGATCCATGGTAAGGAAGTTTGCCAAATCAGATGCTTCCACTTGTAAGCTTGCAAT
GGCTTTCCCAGATGTACTTGCAACATCTCCATTTAGTAGAACCTCCTCTGCACATTTCAA
GAGTGGTCTTGAGAAACCATTTTTCTTATGTGAGGTTGAACCATTCTTGTTC
>GI2_*sg0contig01489_4031-4979
TAACTGGTGTAAGGGTCAGGTGAGCTTCAAGAGGCTCAGCTTTGTTCACAATGGAGGACA
CTGTTTTACCATGCAAATCAATCAAATGGTAAAGGGACGATGCCCTGGTAGAAATCTCAG
TATCCCACTTGCATCGCATCAGTGCAGAGAGTGCATTTAGGCAAGGCCTGGATCTACGAA
ATAACTCAGAAACATGAGCAGCAACCATAGCTGCTGCAACTATTTCATTTGAGCTATAGC
TCCACGAGGTGCCAACTGATGATGGCTTCAGGGAGAAAAGAGCTTCCAAGATGCCAAGTA
TTCTGCGAGTATGAAGAATTGCTGAGTTGATGCTATTTTGTAGCTCGGTATTGATCCCAT
TGGTTTTTCCAGGTACGATAATCTTCATGGAATCCTTTATATTTGAATTTGAACCGTTCT
TAGAAATGAAAGGGAACAGCTGAAGCTCACAAGACAAGGCACAAACTGCAGCTAAAACAT
ATGAATCAAATGTAGCAACAGGTCCCTGTCTCTTCTTACATCTGTTTCTTCCATTTGTTA
ATCTTGAATTCTCAGTGACTTCCTCAGAAGAATGGTTATCACTACCAGTTGGTCTTTTGC
TGCCCCCTGGTAAAGCTTGATGACTGACACACACAGTTAACACCACAAATAGTAACCGGG
AGGCAAGATCCATTGAGGCACAGGATTCAACGAAAAGTGAATGTATCATTGTGCGGAGTT
CAGCGACAGCAAGGTTCTTAGATTGACTTCTTGGTTTTCTGGATTGTTCTGAGGTTTCGG
AAGGAAAAGTTCTCCTGAGTATAGCTTCAACTGTTGCCACAAATATCCTCATCAAGCATG
CTTCAGATGGACTTCCACGAGGTAAGTACTCAAATACTTTTAATAATGGCAAGTACAGGC
TCCATGACAGTGTAGGTGGTTGAAGCGGGGCAGCTACGACAATTTCAGG
>GI2_*sg0contig15400_2735-3683
TAACTGGTGTAAGGGTCAGGTGAGCTTCAAGAGGCTCAGCCTTGTTCACAATGGAGGACA
CTGTTTTACCGTGCAAATCAATCAAATGGTAAAGGGACGATGCCCTGGTAGAAATCTCAG
TATCCCACTTGCATCGCATCAGTGCAGAGAGTGCATTTAGGCACGGCCTGGATCTACGGA
ATAACTCAGAAACATGAGCAGCGACCATAGCTGCTGCAACTATTTCATTTGAGCTATAGC
CCCACGAGGTGCCAACTGATGATGGCTTCAGGGAGAAAAGAGCTTCCAAGATGCCAAGAA
TTCTGCGAGTATGAAGAATTGCTGAGCTGATGCTATTGTGTAGCTCAGTATTGATCCCAT
TGGTTTTTCCAGGTACAATAATCTTCATGGAATCCTTTATACTTGAATGTGAACCGTTCT
TAGAAATGAGAGGGAACAGCTGAAGCTCACAAGACAAGGCACAAACTGCAGCTAAAACAT

ATGAATCAAATGTAGCAACAGGCCCTTGTCTCTTCTTACATCTGTTTCTTCCATTTGTTA
ATCTTGAATTCTCAGCGACCTCCTCAGAAGAATGGTTATCACTACCAGTTGGTCTTTTGC
TGCCCCCTGGTAAAGCTTGATGACTGACACAAACAGTTAGCACCACAAATAGTAACCGGG
AGGCAAGATCCATTGAGGCACAGGATTCAACGAAAAGTGAATGTATCATTGTGCGGAGTT
CAGCGACAGCAAGGTTCTTAGATTGACTTCTTGGTTTTCTGGATTGTTCTGAGGTTTCAG
AAGGAAAAGTTCTCCTGAGTATAGCTTCAACTGTTGCCACAAATATCCTCATCAGGCATG
CTTCAGATGGACTTCCACGAGGTAGGTACTCAAATACTTTTAATAATGGCAAGTACAGGC
TCCATGACAGTGTAGGTGGTTGAAGCGGGGCAGCTACGACAATTTCAGG
>GI3_*sg0contig01489_5801-8135
TGCAGATGCATCCAATTCTTTGGAAGCTAAGAAAAACAAATTGCCACAGTTAATGAAGAT
TTAAACCAAAAAATGATGGTTGGTCATTTTTCTATCATGAGGCAACTTGATAATTCTGCA
CTCAAAAGATAACCTATTAAATATGCCATACCCGCATGCCAGTAGCATAATCTTCTGCTG
CTCTAAGTAGTTCAACTAGTTGCACCGCTGCATCCAGTGCATCTGGAGCCCATGACGGAG
GTGCTTCGAGAAGACCAAAAAGCAGCCTCTGTGTAGCACTTGGAGTAGCAATTGCATAGT
ATCTGACAGGAAAATAACCCATCAATAAATTGTCTGGGAAGTGGATTAAGCACAATAGTG
GTTTGGAGCCTGCCTATGGAACAAACGAGCATAGGGTTCGAGAGGGGGCAGGCCAGCCAC
CAAATGCTCATCCAATGGTGTTGTCGGTGGAGGCAGCAGAAGTGCAGGAACAGCTGCTGC
GGTTAAATTTGCTGTCTCATAACGAGCTACCTCCTCATCACAGACACTTAATATAACACC
AGCTCCATTTGCAACAGCCCATCTGGGGGTGGATGGCATAAGTTGCGGGTGCTTCCCAGA
TCCTCGGCTGCAAGCTAAATAAACCCGAACACATGTTACTCTAGCTTTTTTGCATACATA
ACAAACACTAGCATCACTTGTAAGAAAAAAATGTGCTCCACATTCTAGAGTAAGGAAAGT
ATGCCCACGTGATTTGTTAGCAAAGCACAGAATCAGGAACATGTTGTCACTTAAAGTTCC
AAACATATAAAAGGATCAGGGTTATGATAAGGCGTCGATCTTGTAAAAAATATTTCTCAG
ATAAACAAGAAAGAGAAGAAAAAAAAGTAACGTCTTTCTTTGAATCTATTGATTGTTGAT
CTGTCTATAGTTTATCCTTGTGAGATTTCAGAATACAAAAGTTGTGCCGCAGAATTTCAG
AAACCAAATTTCCAGTTAGATCACAGTAGGGGTACCCCTACTGTTTTCGCTCAAAAAAAA
TCGTCAGATATGGTATATCCACATAAATGACAATGATAAGGCAATAACTACTATAATACT
CCAAATCCGCAACTCAAAGATAGAATACTTGGTTATTGGTTTTACTAACCAGTTGTTGGA
GGCTTCAATTCTCCACCAGCAGCATATTTTCCCATTACACCACCACACCTGAATTGAAAT
GAAATGGAGAGATTAGCCTCAACTACAAAGTAGACATGTAATATAGCAAATTCTTAATTT
TCAGCAGCAAATACATATATTCCAACAAGCTTAAAAGCATTTTGCAGTAGAGAACTAAAA
GTCCCAATCCCATCATTGTATCTATATATACATCACGGTCACCATTTATTTCAGAAGTTT
GTGTCTTGCATAGTTCTGGTTTTTCTTTGTTTCCTCTTTTGCATTGTACAAATATATTTC
TAATAAATATAACATAATCAGGATTGCATTCTCCAAGGAAGTTCAGCAAACAGAAAATGT
CCAATATAAATATTATTACTCACCATCTAAAGTAGTCGCTTCTAATTCCCAAGGGTGCAG
CGAGCAATATGTCAGTGATCCATGGAGTTAGTGGCCTCAATGGCTTCCGATCAGGTTCAT
TTCCTAGGGAATCATCAGATTTCTTATCCGTGGAATCACTGGACGTTGCCTGATCGGATG
TGCTGCTACATTCAGCTTCACAACGTTGGCGCTCAACTTTGAATATTGGCCTATTGTAAT
GAGTCAAGACTCTAAGAATTTCGCCACATGCCAAGGCCCACTGCTCTGAGTACTCTTTCT
GCAGTGAAAATAATGTTAATGAGACAGTATAAAAGTAAAAGTCAAATGCACTTGAACCTA
TCAGGATAGTAAGGGCCAGACCTCAGAAGTATGGCTAAACAAAGAGATGAAGGAGCTGAA
TGGGGGACCATGTCTATCATAGCACAGTGTCCCATCTATGATACGTGAAAGAACTGGATG
TACAACTGCATGACCATGCTCGGGATGATGAAGAACAAAAGTTGCTGTACAAATGTAAGG
AAAAATAATATGTCAGACACTTTGTTGAATTTTTCATGTTCAGTGACATTTTGTATCAGA

ACTCAGAAGTTTGTATTACCCAACACTTCATCTACCAAGCGTTTTTCTTTCCATGGGTAG
CAACTTTGAATGAGCTGCATAAAAGCAAAAGGGAAAGATACACAAGTTAAACACATTTGG
CATATATTTGCCACAACATATACTAAGTTAGTGTACAAGATGTGGTACCTGTGCTACATC
TTCAGGGAATTGCTCGCTGTCAGCTGTAAACTGGCCAAAGTACTCAACATAGGCC
>GI3_*sg0contig15400_4622-6945
TGTAGATGCATCCAATTCTTTGGAAGCTAGGAAAAACAAATTGCCACAGTTAATAAGATT
TAAACCAAAGATGATGGTTGGACAATTTTCTATCATGAGGCAACTTGATAATTCTGCACT
GAGGCAACTTGATAATTCTGCACTCAAAAGATAACCTATTAAATATGCCATACCCGCATG
CCAGTAGCATAATCTTCTGCTGCTCTAAGTAGTTCAACTAGTTGCACCGCTGCATCCAGT
GCATCTGGAGCCCATGATGGAGGTGCTTCGAGAAGACCAAAAAGCAGCCTCTGTGTAGCA
CTTGGAGTAGCAATTGCATAGTATCTGATAGGAAAATAACCCATCAATAAATTATCTGGG
AAGTGGATTAAGCACAATAGTGGATTGGAGCTTGCCTATGGAACAAACGAGCATATGGTT
CGAGAGGGGGCAGGCCAGCCACCAAATGCTCATCCAATGGTGTTGTCGGTGGAGGTAGCA
GAAGCGCAGGAACAGCTGCTGCGGTTAAATTTGCTGTCTCATAACGAGCTACCTCCTCAT
CACAGACACTTAATATAACACCAGCTCCATTGGCAACAGCCCATCTGGGGGTGGATGGCA
TAAGTTGCGGGTGCTTCCCAGATCCTCGGCTGCAAGCTAAATAACCCAAACACATGTTAC
TCTAGAGTTTTTGCATACATAACAAACATTTGCATCACTTATAAGAAAAAAAAAGTGCTC
CACATTCTAGAGTAAGGAAAGTATGCCCACGTGATTTGTTAGCAAAGCACAGAATCAGGA
ACATGTTTGTCACTTAAAGTTCCAAACATATAAAAGGATCAGGGTTATGATTAAGGGGTC
GATGTTGTAAAAAAATTTCTCAGATAAACAAGAAAGAGAAGAAAAAAAGTCTTCCTTTGT
ATCTACTGATTATTAATCTGTCTATAGTTTATCCTTGTGAGATTTCAGAATACAAAAGTT
GTGCCGCAGAATTTCAGAAACCAAAATTCCAGTTAAATCACAGTAGGGGTAACCCAACTG
CTTTCGCTAAAAAATATCAAGTTAGATACGGTATATCCACATAACTGACAATGATAAGGC
AATAATCTGCAACTCAAAGATAGAATATTTGGTTATTGGTTTTACTAACCAGTTGTTGGA
GGCTTCAATTCTCCACCAGCAGCATATTTTCCCATTACACCACCACACCTGAATTGAAAG
GAACGGAGTGATTAGCCTCAATTACAAAGTAGACATGTAATATAGCAAATACTTCATTTT
CAGCAGCAAGTACATTATATTCCAACAAGCTTAAAAGCATTTCACAGCAGAGAACTAAAA
CTCCCATCATTGTATCTATATATACATCACGGTCACCATTTATTTCAGAAGTTTGTGTCT
TGCATAGTCCTGGGTTTTTCTTTGTTTCTTCTTTTGCATTGTGCAAATATATTTCTAATA
AATATAACATAATCAGGATCACATTCTCCAAGGAAGTTCAGCAAACAGAAAATGTCAAAT
ATAAATATTACTACTCACCATCTAAAGTAGTCGCTTCTAATTCCCAAAGGTGCAGCGAGC
AATATGTCAGTGATCCATGGAGTTAATGGCCTCAATGGCTTCCGATCAGGTTCATTTCCT
GGGGAACCATCAGATTTCTCATCCGTGGAATCACTGGATGTTGCCTGATCAGATGTGCTG
CTACATTCAGCTTCACAACGTTGGCGCTCAGCTTTGAATATTGGCCTATTGTAGTGAGTC
AAGACTCTGAGAATTTCACCACATGCCAAGGCCCACTGCTCTGAGTACTCTTTCTGCAGT
GAAAATAATGTTCATGAGACAGTATAAAAGTAAAAATGAAATGCAGTTGAACCTGTCAGG
ATAGTAAGGGCCTGACCTCAGAAGTATGACTAAACAAAGAGATGAAGGAGCTGAATGGGG
GACCATGTCTATCATAGCACAGTGTCCCATCTATGATACGTGAAAGAATTGGATGTACAA
CTGCATGACCATGCTCGGGATGATGAAGAACAAAAGTTGCTGTACAAATGTAAGGAAAAA
TAATATGTCAGACACTTTGTTGAATTTTTCATATTCAGTGACATTTTGTATCAGAACTCA
GAAGTTTGTATTACCCAACACTTCATCTACCAAGCGTTTTTCTTTCGATGGGTAGCAACT
TTGAATGAGCTGCATAGAAGCAAAAGGAGATACACAAGTTAAATACATTTGGCATATATT
TTGCCACAACATATACTAAGTGTACAGGATGTGGTACCTGTGCTACATCTTCAGGGAATT
GCTCGCTGTCAGCTGTAAACTGGCCAAAGTACTCAACATAGGCC

Table S4. Number of amplicon reads mapped to each of the 56 reference switchgrass contigs

Gene	Contig	Chromosome: start..end	# mapped reads
DW3	Dw3_*sg0contig117938_2825-628	Chr06N:79134518..79136715	2,391,876
DW3	Dw3_*sg0contig26301_4059-6265	Chr06K:72381688..72383894	2,779,831
DW3	Dw3_sg0contig03796_3522-6032	Chr01N:83034824..83032314	724
DW3	Dw3_sg0contig18476_9040-7900	Chr07N:47182836..47181696	11
DW3	Dw3_sg0contig26408_1683-2829r	Chr07K:49068347..49067201	12
DW3	Dw3_sg0contig43647_2891-5451	Chr01K: 71351478..71348918	2,426
FLD	FLD_*sg0contig01920_13106-15435	Chr07N:58360301..58362630	5,801,211
FLD	FLD_*sg0contig102960_2518-190	Chr07K:60034595..60036923	6,338,176
FLD	FLD_sg0contig114184_653-1198r	Chr06N:11456689..114572234	6
FLD	FLD_sg0contig16503_1111-2769r	Chr09N:96460773..96459115	6
FLD	FLD_sg0contig20763_3886-5544r	Chr01N:93893678..93895336	4
FLD	FLD_sg0contig269427_753-1298r	Chr06K:3637699..3638244	5
FLT	FLT_*sg0contig08422_1606-3786	Chr07N:49971808..49973988	3,294,618
FLT	FLT_*sg0contig09545_9355-11180	Chr07K:52187071..52188897	8,718,922
FLT	FLT_sg0contig18299_5751-6535r	scaffold_794:19166..18382	23
FLT	FLT_sg0contig241270_448-642r	Chr08N:50869292..50869098	11
FLT	FLT_sg0contig31169_2577-2711	scaffold_6524:4191..4325	-
FLT	FLT_sg0contig34686_1257-1858r	Chr01K:61589681..61589080	54
GI	GI1_*sg0contig01489_2837-3966	Chr05N:7183267..7182138	3,085,799
GI	GI1_*sg0contig15400_1539-2670	Chr05K:8134094..8132963	4,686,512
GI	GI2_*sg0contig01489_4031-4979	Chr05N:7182073..7181125	2,723,749
GI	GI2_*sg0contig15400_2735-3683	Chr05K:8132898..8131950	3,811,089
GI	GI3_*sg0contig01489_5801-8135	Chr05N:7180303..7177968	3,501,266
GI	GI3_*sg0contig15400_4622-6945	Chr05K:8131011..8128688	3,396,847
HD1	Hd1_*sg0contig03275_4535-6757	Chr04K:22322250..22320027	1,989,152
HD1	Hd1_*sg0contig05584_7137-9276	Chr04N:29069974..29072113	5,365,447
PHYB	Ma3_*sg0contig13571_3958-761	Chr09N:98235530..98238727	10,030,920
PHYB	Ma3_*sg0contig21054_5003-1810	Chr09K:69988732..69990632	14,101,927
PHYB	Ma3_sg0contig00216_6133-7266r	Chr09N:12253888..12252755	-
PHYB	Ma3_sg0contig00846_10377-11312	Chr03K:34831072..34832007	8
PHYB	Ma3_sg0contig03093_10032-9091	Chr09N:9715266..9716207	39
PHYB	Ma3_sg0contig06603_6712-7842	Chr05K:51115975..51117105	4
PGM	PGM_sg0contig17299_6038-9157	Chr09K:68930489..68927308	5,073,487
PGM	PGM_sg0contig181405_20-2260		227,133

PGM	PGM_sg0contig191126_72-2290		123,749	
PGM	PGM_sg0contig200892_1560-2255r	Chr09K:68928015..68927319	1,036,271	Chr09N[#]
PGM	PGM_sg0contig320997_sg0contig338773_1-293_1-1319r	scaffold_23804:1319..1	1,434,062	
PGM	PGM_sg0contig338921_1-488r	Chr09N:94519823..94519336	686,524	
PGM	PGM_sg0contig341962_1-438r	Chr09N:94519823..94519381	66,869	
PHYC	PhytC1_*sg0contig00846_6259-9792	Chr03K:34826954..34830487	3,876,544	Chr09K[#]
PHYC	PhytC1_*sg0contig03093_13282-10617	Chr09N:9712016..9714681	8,370,255	
PHYC	PhytC1_sg0contig00216_7872-8174r	Chr09N:12254796..12254494	-	
PHYC	PhytC1_sg0contig06603_5819-6121	Chr09K:51115082..51115384	-	
PHYC	PhytC2_*sg0contig00846_9819-10376	Chr03K:34830514..34831071	1,914	Chr09K[#]
PHYC	PhytC2_*sg0contig03093_10590-10033	Chr09N:9714708..9715265	3,226,168	
RHT1	Rht1_*sg0contig15005_4739-6608	Chr09N:13422817..13424686	1,723,506	
RHT1	Rht1_*sg0contig35590_2125-3984r	Chr09K:2359585..2361445	1,974,882	
TB1	Tb1_*sg0contig06045_3865-6556nogap	Chr09K:2412986..2410173	3,761,810	
TB1	Tb1_*sg0contig76312_641-42329nogap	Chr09N:13498215..13495780	19,810,738	
TE	TE_*sg0cons_contig99597_contig278542_contig361965		3,612,201	
TE	TE_*sg0contig04674_63-1916	Chr09N:28516531..28518384	2,784,231	Chr05N[#]
VRN3	Vrn3_*sg0contig07490_4864-7121	Chr05N:47934661..47936918	16,315,087	Chr03N[#]
VRN3	Vrn3_*sg0contig16433_4192-6449	Chr03K:60686632..60684373	11,117,408	
VRN3	Vrn3_sg0contig00021_43010-45413r	Chr03K:13799482..13797079	80	
VRN3	Vrn3_sg0contig38175_2664-2882	Chr03N:35362288..35362506	10	
VRN3	Vrn3_sg0contig49546_1490-3897	Chr03K:13597796..13595389	17,592	
Total			167,261,196	

[#] Corrected based on mapping data, P Qi and KM Devos, unpublished data

Table S5. Summary statistics for the non-synonymous SNPs analyzed in 12 biomass genes.

							SNP allele			Amino Acid				Allele frequencies %			Frequency of wild-type allele in each genetic subpopulation %				SNP characteristics					
Gene	Contig	Chromosome	SNP position in contig	Amino Acid position in *Setaria Reference* sequence	SNP region	*Setaria italica* reference sequence	Ref	Alt1	Alt2	Ref	Alt1	Alt2	Setaria italica	Wild-type allele	Mutant 1	Mutant 2	C1	C2	C3	Admix	Conservative (Con) vs. non conservative (Non Con)	Common vs. Rare	Population for which SNP (corresponding amion acid is in parenthesis) is prevalent	Population for which SNP (corresponding amion acid is in parenthesis) is diagnostic	Domain	Mixed accessions:genetic group Amino Acid
GI	contig15400	Chr05K	1694	155	Exon7	Si000107m	G	A		**E**	K		E	91.59	8.41		77.78	100.00	100.00	98.04	Con	rare	C1(K)-C2C3(**E**)			
GI	contig15400	Chr05K	1633	175	Exon7	Si000107m	C	A		**S**	Y		S	80.32	19.68		48.31	100.00	100.00	100.00	Non Con	rare	C1(Y)-C2C3(**S**)	C1(Y)		
GI	contig15400	Chr05K	1627	177	Exon7	Si000107m	G	A		G	**D**		D	32.80	67.20		79.67	0.00	4.48	2.13	Non Con	Common	C1(**D**)-C2C3(G)	C1(**D**)		PI 431575:C1+G
GI	contig15400	Chr05K	1619	180	Exon7	Si000107m	G	A		**G**	R		G	92.79	7.21		81.25	100.00	100.00	98.04	Non Con	rare	C1(R)-C2C3(**G**)			
GI	contig15400	Chr05K	633	234	Exon9	Si000107m	G	T	A	**C**	F	Y	C	79.82	19.59	0.58	92.54	68.42	43.14	74.47	Non Con	rare				
GI	contig01489	Chr05N	2152	63	Exon5	Si000107m	G	C		W	**S**		S	37.50	62.50		94.06	0.00	5.00	9.52	Non Con	rare	C1(**S**)-C2C3(W)	C1(**S**)		
GI	contig01489	Chr05N	1608	185	Exon7	Si000107m	G	A		**R**	Q		R	65.84	34.16		13.86	100.00	100.00	80.85	Con	Common	C1(Q)-C2C3(**R**)	C1(Q)		PI 431575:C1+R
GI	contig01489	Chr05N	426	960	Exon14	Si000107m	T	G		F	**L**		L	35.83	64.17		90.72	0.00	1.47	15.22	Non Con	Common	C1(**L**)-C2C3(F)	C1(**L**)		PI 431575:C1+F
PHYB	contig13571	Chr09N	1443	661	Exon2	Si033968m	A	T		**S**	C		S	63.64	36.36		8.20	100.00	100.00	92.16	Non Con	Common	C1(C)-C2C3(**S**)	C1(C)		PI 414067:C1+S
PHYB	contig13571	Chr09N	1320	702	Exon2	Si033968m	G	A		**V**	I		V	79.13	20.87		100.00	17.33	95.65	86.27	Con	rare	C2(I)-C1C3(**V**)	C2(I)	PAS domain	
PHYB	contig13571	Chr09N	1287	713	Exon2	Si033968m	T	G		Y	**D**		D	50.30	49.70		97.99	4.00	15.15	21.74	Non Con	balanced	C1(**D**)-C2C3(Y)	C2C3(Y)	PAS domain	PI 315727:C3+D
HD1	contig03275	Chr04K	1868	11	Exon1	Seita.4G122700.1	G	A		**E**	M		E	85.98	14.02		60.00	100.00	98.39	97.92	Non Con	rare				
HD1	contig03275	Chr04K	1805	32	Exon1	Seita.4G122700.1	G	A		**A**	T		A	94.56	5.44		99.19	76.56	100.00	100.00	Non Con	rare			Zinc binding domain	
HD1	contig03275	Chr04K	1796	35	Exon1	Seita.4G122700.1	G	A		G	**S**		S	60.63	39.37		96.67	0.00	81.13	38.30	Non Con	Common	C2(G)-C1C3(**S**)	C1C3(**S**)	Zinc binding domain	PI 422016, SPBluff:C3+G
PGM	contig17299	Chr09K	2004	351	Exon6	Si034948m	C	T		**A**	V		A	73.21	26.79		31.82	100.00	86.89	86.27	Non Con	common			Alkaline-phosphatase-like, core domain	None
TB1	contig06045	Chr09K	478	57	Exon1	Si038692m	G	A		**G**	D		G	91.61	8.39		100.00	100.00	58.33	100.00	Non Con	rare	C3(D)-C1C2(**G**)			
TB1	contig06045	Chr09K	937	213	Exon1	Si038692m	G	D		**G**	D		G	89.76	10.24		100.00	44.68	100.00	96.77	Non Con	rare	C2(D)-C1C3(**G**)	C2(D)	Transcription factor, TCP	
TB1	contig06045	Chr09K	1305	337	Exon1	Si038692m	A	G		**I**	V		L	56.64	43.36		9.35	98.31	98.31	71.43	Con	balanced	C1(V)-C2C3(**I**)	C1(V)		
TB1	contig76312	Chr09N	571	51	Exon1	Si038692m	C	T		**H**	Y		H	88.06	11.94		72.32	100.00	95.31	94.44	Con	rare				
TB1	contig76312	Chr09N	688	89	Exon1	Si038692m	G	C		**A**	P		A	92.42	7.58		100.00	100.00	57.63	100.00	Non Con	rare	C3(P)-C1C2(**A**)			
TB1	contig76312	Chr09N	826	137	Exon1	Si038692m	T	C		**S**	P		S	92.77	7.23		100.00	100.00	58.18	100.00	Non Con	rare	C3(P)-C1C2(**S**)		Transcription factor, TCP	
TB1	contig76312	Chr09N	994	193	Exon1	Si038692m	A	G		I	**V**		V	60.26	39.74		98.50	1.41	63.46	36.96	Con	Common		C1C3(V)	Transcription factor, TCP	
TB1	contig76312	Chr09N	1385	321	Exon1	Si038692m	A	G		**N**	S		N	91.87	8.13		99.28	100.00	55.17	100.00	Con	rare				
PHYC	contig03093	Chr09N	365	422	Exon1	Si034030m	G	C		V	**L**		L	45.71	54.29		48.53	65.33	57.14	50.00	Con	balanced			Phytochrome central region, GAF domain like	
PHYC	contig03093	Chr09N	1048	966	Exon3	Si034030m	A	T		E	**V**		V	42.90	57.10		95.00	1.28	4.69	10.20	Non Con	balanced	C1(**V**)-C2C3(E)	C1(**V**)-C2C3(E)	Histidine kinase-like ATPase, ATP-binding domain	None
PHYC	contig03093	Chr09N	860	1029	Exon3	Si034030m	C	G		**P**	A		P	81.03	18.97		93.75	86.67	46.00	69.05	Non Con	rare			Histidine kinase-like ATPase, ATP-binding domain	
PHYC	contig03093	Chr09N	854	1031	Exon3	Si034030m	A	G		**K**	E		K	76.66	23.34		45.08	97.47	96.83	94.34	Con	rare		C1(E)	Histidine kinase-like ATPase, ATP-binding domain	
PHYC	contig03093	Chr09N	822	1041	Exon3	Si034030m	G	T		**K**	N		K	83.44	16.56		59.20	100.00	100.00	95.92	Non Con	rare	C1(N)-C2C3(**K**)		Histidine kinase-like ATPase, ATP-binding domain	
PHYC	contig03093	Chr09N	178	1069	Exon1	Si034030m	C	T		**L**	W		L	94.26	5.74		100.00	98.77	77.19	89.13	Non Con	rare			Histidine kinase-like ATPase, ATP-binding domain	
PHYC	contig03093	Chr09N	72	1104	Exon1	Si034030m	T	A		**L**	H		L	67.70	32.30		31.50	94.81	90.77	86.79	Non Con	common			Histidine kinase-like ATPase, ATP-binding domain	PI 414068, PI 421520, PI 642191, PI 337553:C1+L

Gene	Contig	Chromosome	SNP position in contig	Amino Acid position in *Setaria Reference* sequence	SNP region	*Setaria italica* reference sequence	SNP allele			Amino Acid				Allele frequencies %			Frequency of wild-type allele in each genetic subpopulation %				SNP characteristics					
							Ref	Alt1	Alt2	Ref	Alt1	Alt2	Setaria italica	Wild-type allele	Mutant 1	Mutant 2	C1	C2	C3	Admix	Conservative (Con) vs. non conservative (Non Con)	Common vs. Rare	Population for which SNP (corresponding amion acid is in parenthesis) is prevalent	Population for which SNP (corresponding amion acid is in parenthesis) is diagnostic	Domain	Mixed accessions:genetic group Amino Acid
VRN3	contig07490	Chr03N*	365	238	Exon2	Si021330m	A	T		**E**	D		E	89.78	10.22		100.00	68.57	93.85	85.00	Con	rare			The plant homeodomain (PHD) finger domain	
VRN3	contig07490	Chr03N*	845	286	Exon3	Si021330m	C	A		**L**	M		L	93.11	6.89		100.00	80.00	98.48	85.71	Con	rare				
VRN3	contig07490	Chr03N*	872	295	Exon3	Si021330m	A	G		**T**	A		T	90.10	9.90		76.86	100.00	98.46	95.92	Non Con	rare				
VRN3	contig07490	Chr03N*	1319	412	Exon4	Si021330m	C	G		**P**	R		P	91.53	8.47		79.13	100.00	100.00	95.92	Non Con	rare	C1(**R**)-C2C3(**P**)		Fibronectin type III	
VRN3	contig07490	Chr03N*	1562	493	Exon4	Si021330m	T	C		L	**S**		S	84.26	15.74		100.00	47.37	98.46	78.72	Non Con	rare		C2(L)		
VRN3	contig07490	Chr03N*	1617	511	Exon4	Si021330m	T	G		**N**	K		N	93.63	6.37		96.12	89.47	100.00	84.09	Non Con	rare				
VRN3	contig07490	Chr03N*	1883	600	Exon4	Si021330m	G	T		**G**	V		E	88.59	11.41		90.37	90.41	92.16	74.36	Non Con	rare				
VRN3	contig16433	Chr03K	81	144	Exon2	Si021330m	C	G		**P**	A		P	90.45	9.55		78.52	100.00	98.55	96.08	Non Con	rare				
VRN3	contig16433	Chr03K	1273	398	Exon4	Si021330m	G	A		**V**	I		A	93.98	6.02		97.93	94.59	85.71	92.00	Con	rare			Fibronectin type III	
VRN3	contig16433	Chr03K	1307	409	Exon4	Si021330m	G	A		**S**	N		S	89.42	10.58		100.00	97.33	54.72	81.58	Con	rare			Fibronectin type III	
VRN3	contig16433	Chr03K	1430	450	Exon4	Si021330m	A	G		D	**G**		G	43.61	56.39		28.68	9.09	95.38	68.00	Non Con	balanced		C1C2(D)	Fibronectin type III	PI 642191, Pasco Co-FL:C1+G
VRN3	contig16433	Chr03K	1786	569	Exon4	Si021330m	C	G		**Q**	E		R	88.36	11.64		77.86	100.00	95.65	87.27	Con	rare				
VRN3	contig16433	Chr03K	2126	682	Exon4	Si021330m	T	A		L	**Q**		Q	28.61	71.39		2.72	17.95	86.15	46.94	Non Con	Common	C3(**Q**)-C1C2(L)			None
DW3	contig26301	Chr06K	250	672	Exon3	Si013123m	A	G		**E**	G		E	94.29	5.71		100.00	70.18	100.00	97.83	Non Con	rare	C2(G)-C1C3(**E**)	C2(G)-C1C3(**E**)	ABC transporter-like, P-loop containing nucleoside triphosphate	
DW3	contig26301	Chr06K	480	751	Exon3	Si013123m	A	G		**M**	V		M	21.22	78.78		0.66	78.79	5.77	24.39	Con	rare			AAA+ ATPase domain	
DW3	contig117938	Chr06N	383	713	Exon3	Si013123m	A	G		I	**M**		M	87.84	12.16		99.35	43.55	100.00	90.00	Con	rare		C2(I)	AAA+ ATPase domain	
DW3	contig117938	Chr06N	918	897	Exon3	Si013123m	A	G		I	**V**		V	82.96	17.04		100.00	24.59	96.15	88.37	Con	rare	C2(I)-C1C3(**V**)	C2(I)	ABC transporter, transmembrane domain	
DW3	contig117938	Chr06N	855	872	Exon3	Si013123m	A	G	T	T	**A**	S	A	78.04	21.96	0.30	92.41	63.93	90.20	91.67	Non Con	rare			ABC transporter, transmembrane domain	
FLD	contig01920	Chr07N	1037	511	Exon1	Si009376	A	G		S	**G**		G	76.70	23.30		100.00	9.72	96.92	74.47	Non Con	rare	C2(S)-C1C3(**G**)	C2(S)	FAD-linked reductases, C-terminal domain superfamily, FAD/NAD(P)-binding domain	

* Corrected based on mapping data, P Qi and KM Devos, unpublished data

Table S6. Genic regions for which the SNP distribution is different in the K and N subgenomes. The percentage of SNPs and the region in which they are located are given for each subgenome.

Gene	Chromosome	Region	Subgenome N	Subgenome K
FLT	Chr07	5'UTR	67% : first 1 Kb	82% : last 1 Kb
PhyB	Chr09	Exon2-Exon3	67%: Exon2	100%: Intron2-Exon3
PGM	Chr09	Exon7-Exon9	67%: Intron8-Exon9	100%: Exon7-Exon8
TE	Chr05	Intron4	69%: first 600 bp	72%: first 600 bp

Table S7. Tajima's, and Fu and Li's tests on a per gene basis within each subpopulation

			Tajima's D			Fu and Li's F		
**Gene**	**Contig**	**Chromosome**	**C1**	**C2**	**C3**	**C1**	**C2**	**C3**
PHYC	contig03093	Chr09N	0.32485	-0.97689	0.78247	0.20117	0.36972	1.50727
TE	contig99597	Chr05K	-0.4587	-0.24132	-1.49999	-0.13062	-0.10201	-2.01579
TE	contig04674	Chr05N#	-1.1022	-0.32402	-0.66677	-0.4166	1.26766	0.62395
VRN3	contig07490	Chr03N#	0.17628	0.20942	0.15019	1.0621	0.93086	0.36214
VRN3	contig16433	Chr03K	0.15973	1.12296	-1.75121	1.05476	1.54981	-0.27781
DW3	contig26301	Chr06K	-0.75669	0.68766	1.48067	0.06957	0.17489	1.2962
DW3	contig117938	Chr06N	-0.39647	1.74821	-0.509	0.43529	0.59643	0.34411
FLD	contig102960	Chr07K	-0.31194	1.73634	-0.90666	0.37221	1.00306	0.05931
FLD	contig01920	Chr07N	-0.90549	-0.229	-0.74552	-2.20553	0.29502	0.11693
GI	contig15400	Chr05K	2.04149	1.40334	-1.03192	1.26945	-0.01727	-0.12792
GI	contig01489	Chr05N	0.09673	0.08522	-1.38756	0.42227	-0.82988	-0.9117
FLT	contig09545	Chr07K	-0.72004	-0.34434	-1.41089	-0.42848	-0.52887	-1.57932
FLT	contig08422	Chr07N	-0.83985	2.32075*	-0.31554	0.00575	1.85456*	0.23638
PHYB	contig13571	Chr09N	0.05747	-1.31321*	0.70423	0.92685	-2.91067*	1.07262
PHYB	contig21054	Chr09K	-0.50911	0.14053	0.26555	0.39101	0.68333	0.73891
HD1	contig03275	Chr04K	-0.33588	1.26989	-0.39367	0.83467	0.22737	0.61529
HD1	contig05584	Chr04N	-0.94075	0.84736	-0.74309	-0.43491	0.95239	0.50158
PGM	contig17299	Chr09K	-0.18887	0.74958	0.03886	0.66441	1.25983	0.87128
PGM	contig200892	Chr09N#	1.31569	1.73749	0.66907	1.28187	1.47586	1.23338
TB1	contig06045	Chr09K	-0.51331	-0.931	1.75946	0.68969	-1.08443	1.22513
TB1	contig76312	Chr09N	-0.36842	0.06178	0.59901	0.29627	-0.41796	1.67188

*Significant value, P < 0.05

Corrected based on mapping data, P Qi and KM Devos, unpublished data

Figure S1. Distances between SNPs

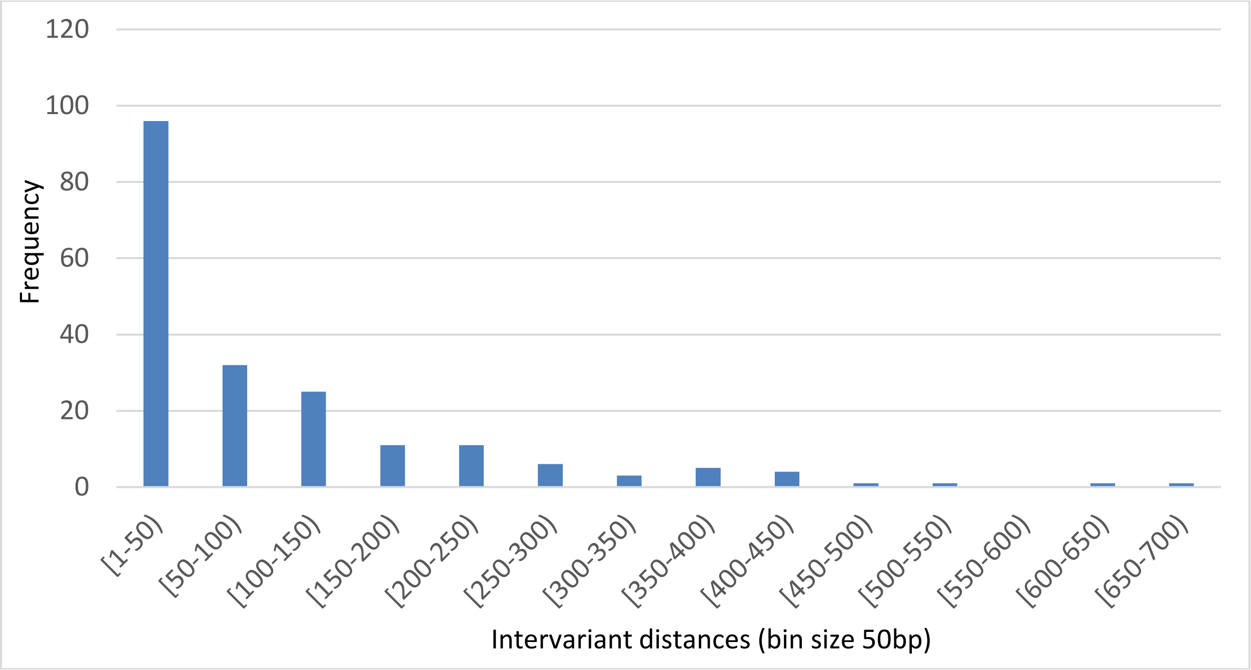

Figure S2. Log probability of data as a function of K. STRUCTURE was run for K ranging from 1 to 10, and 10 repetitions were performed with 100000 burn-ins and 100000 runs. K=3 clusters were retained as the most likely number of genetic clusters among the switchgrass population.

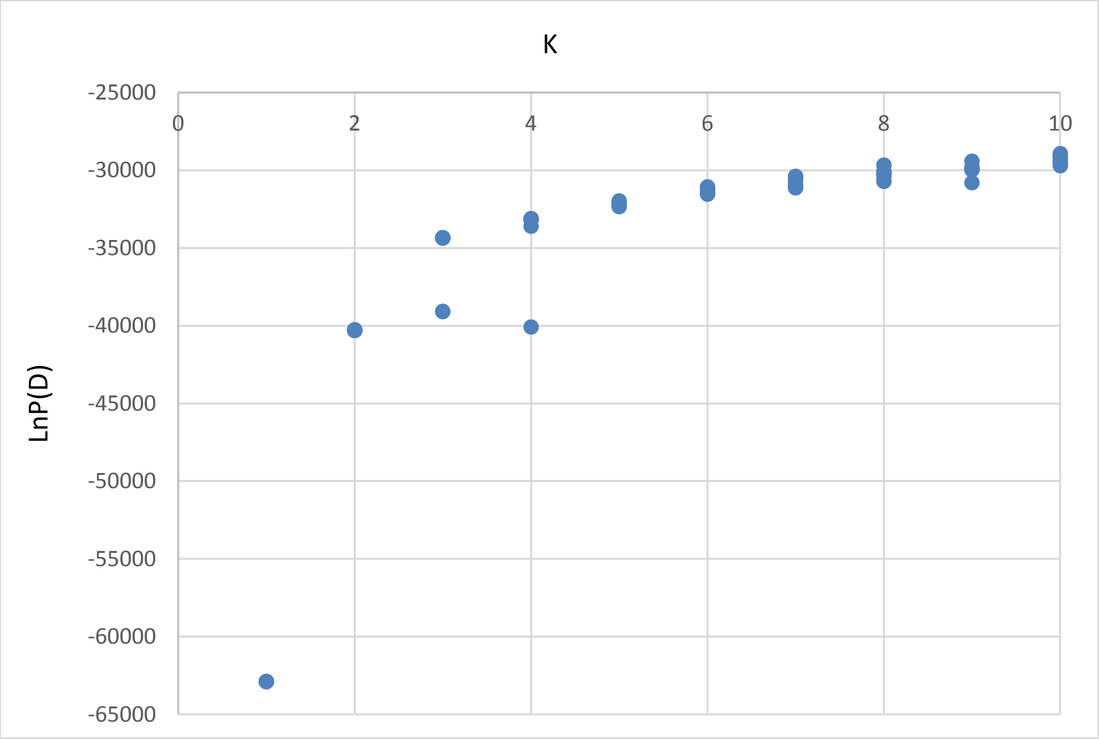

Figure S3. UPGMA tree performed on the 251 SNPs across the 372 genotypes with a 500 replicates bootstrap test using Mega 6[60] based on the maximum composite likelihood method. C1, C2 and C3 clusters are colored in blue, green and red respectively; admixed individuals are in gray.

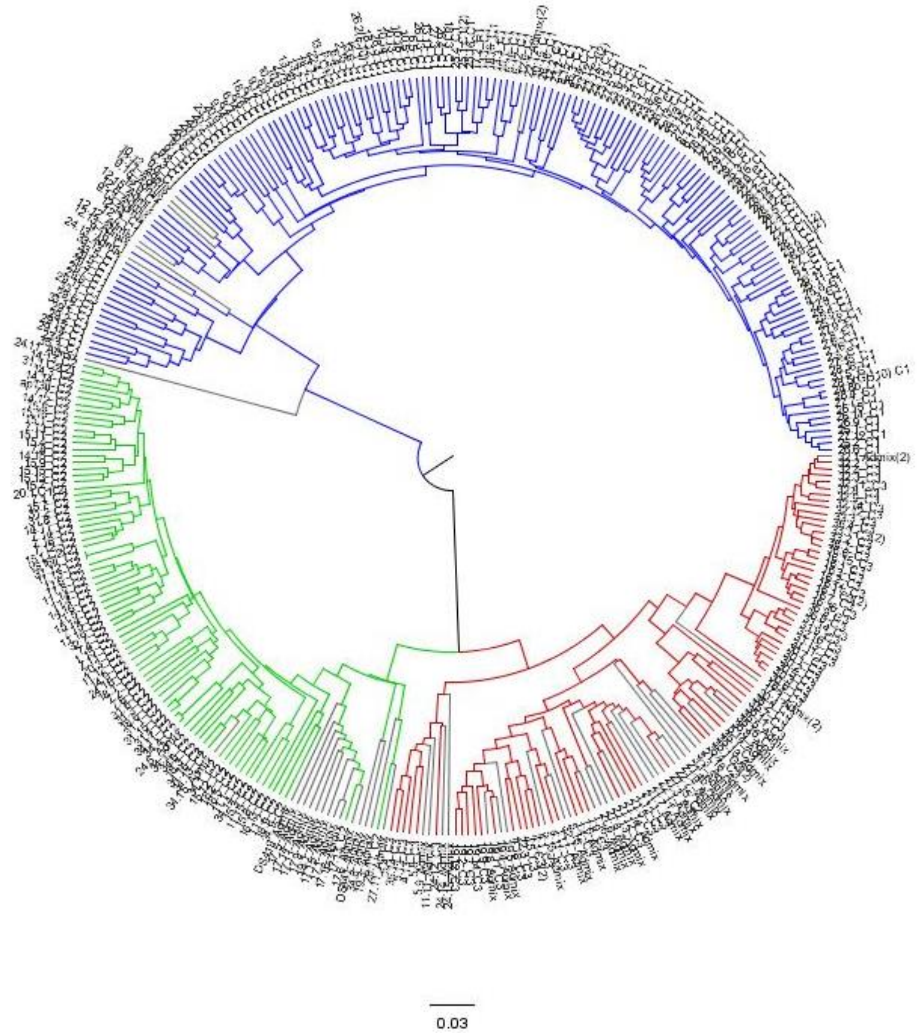

Figure S4. Local Indicator of Spatial Autocorrelation Analysis (2D-LSA) on 372 genotypes. Individuals that are consistently significantly more related to their 7 to 14 nearest neighbors than to random individuals are represented as plain blue dots. The number of genotypes is given in parenthesis. Accessions with significant P values for more than 90% of the genotypes are listed; their subpopulation and number of genotypes are indicated. USA Map source: https://upload.wikimedia.org/wikipedia/commons/c/ca/Blank_US_map_borders.svg.

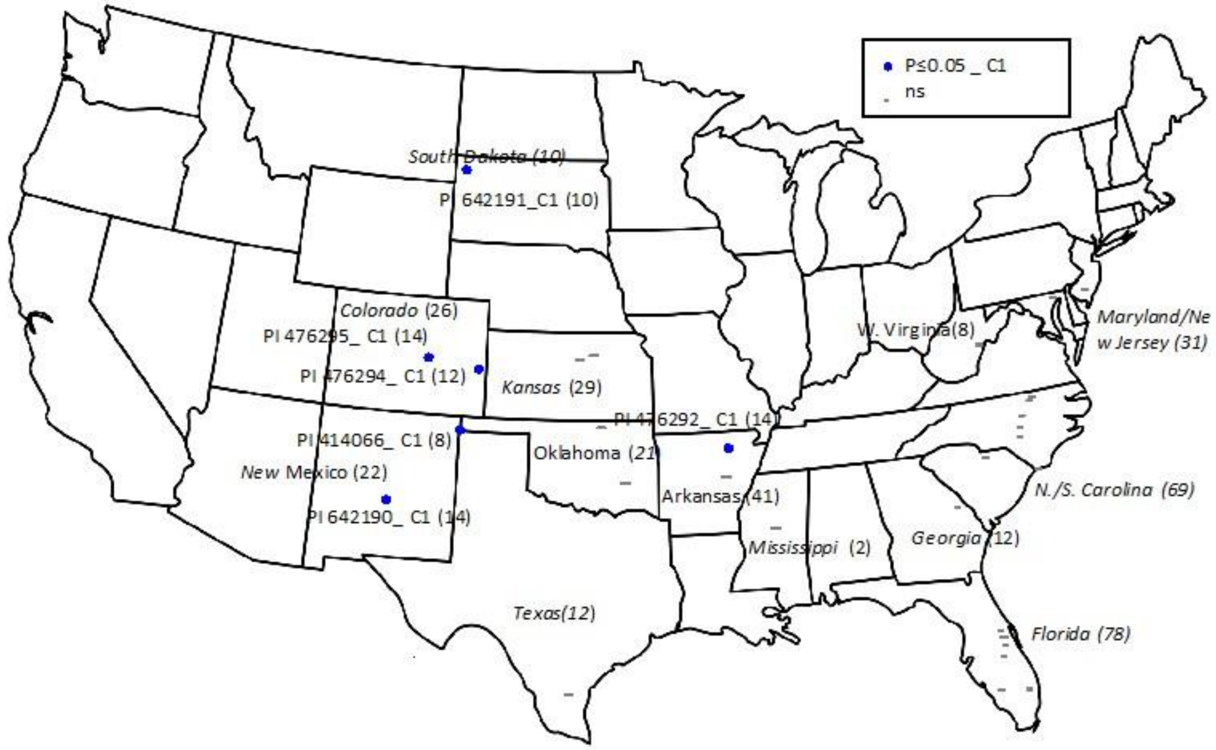

Figure S5. Regression analysis of the percentage of polymorphic loci and latitude bins across the switchgrass accessions.

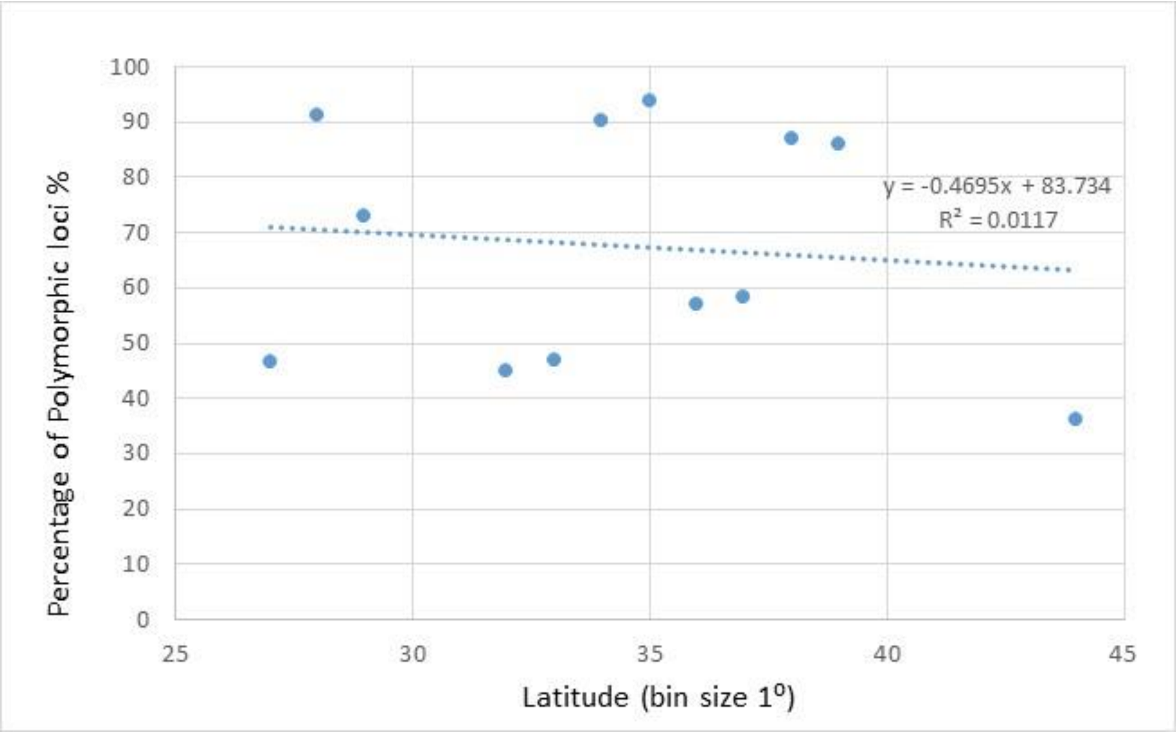

Figure S6. Protein structure modelling of an amino acid substitution in the PAS domain of 1D06, a protein with similar PAS domain as PHYB (A) Original structure of protein 1D06; (B) modified structure after 2 amino-acid changes in the PAS domain (Yellow): one conservative substitution (Val -> Ile; in green) and one non-conservative substitution (Asp -> Tyr; in pink). Swiss-Pdb Viewer 4.1.0 [70] was used to visualize the crystal structure.

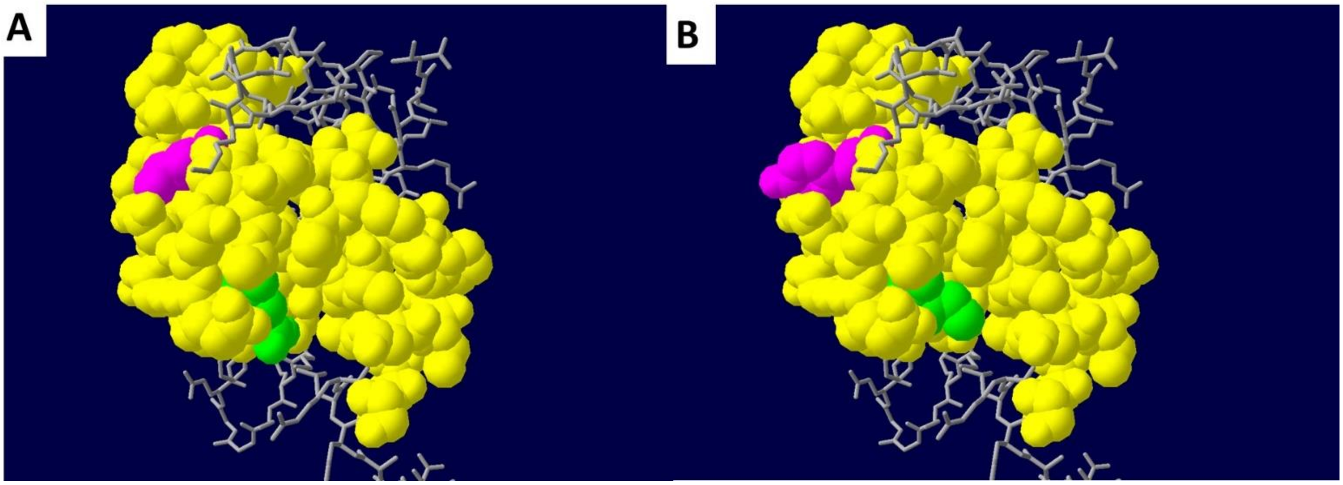

Supplement: Supplementary file 1 — Table S1. List of switchgrass accessions used in the study with their ID and name, number of genotypes, ecotype identification, ploidy level, state of origin, and GPS coordinates [114–116]. Table S2. Sequences and annealing temperatures of the 33 primer pairs used for PCR amplification of the selected 12 genes. Conserved regions in orthologous exons in Oryza sativa (rice), Sorghum bicolor (sorghum), Zea mays (maize) and Setaria italica (foxtail millet) were used for primer design. Table S3. Sequences of 56 regions of AP13 extracted from the Phytozome database (http://www.phytozome.net/), and used as reference for read mapping and SNP identification. Table S4. Number of amplicon reads mapped to each of the 56 reference switchgrass contigs. Table S5. Summary statistics for the non-synonymous SNPs analyzed in 12 biomass genes. Table S6. Genic regions for which the SNP distribution is different in the K and N subgenomes. The percentage of SNPs and the region in which they are located are given for each subgenome. Table S7. Tajima’s, and Fu and Li’s tests on a per gene basis within each subpopulation. Figure S1. Distance between SNPs. Figure S2. Log probability of data as a function of K. STRUCTURE was run for K ranging from 1 to 10, and 10 repetitions were performed with 100,000 burn-ins and 100,000 runs. K = 3 clusters were retained as the most likely number of genetic clusters in the switchgrass panel analyzed. Figure S3. UPGMA tree performed on the 251 SNPs across the 372 genotypes with a 500 replicates bootstrap test using Mega 6 [60] based on the maximum composite likelihood method. C1, C2 and C3 clusters are colored in blue, green and red respectively; admixed individuals are in gray. Figure S4. Local Indicator of Spatial Autocorrelation Analysis (2D-LSA) on 372 genotypes. Individuals that are consistently significantly more related to their 7 to 14 nearest neighbors than to random individuals are represented as plain blue dots. The number of genotypes is given in [file 12862_2018_1193_MOESM1_ESM.pdf]
